# Radiogenomics Pilot Study: Association Between Radiomics and Single Nucleotide Polymorphism-Based Microarray Copy Number Variation in Diagnosing Renal Oncocytoma and Chromophobe Renal Cell Carcinoma

**DOI:** 10.3390/ijms252312512

**Published:** 2024-11-21

**Authors:** Abeer J. Alhussaini, Abirami Veluchamy, Adel Jawli, Neil Kernohan, Benjie Tang, Colin N. A. Palmer, J. Douglas Steele, Ghulam Nabi

**Affiliations:** 1 Division of Imaging Sciences and Technology, School of Medicine, Ninewells Hospital, University of Dundee, Dundee DD1 9SY, UK; a.j.a.h.m.alhussaini@dundee.ac.uk (A.J.A.); ajawli@dundee.ac.uk (A.J.); d.steele@dundee.ac.uk (J.D.S.); g.nabi@dundee.ac.uk (G.N.); 2 Division of Neuroscience, School of Medicine, Ninewells Hospital, University of Dundee, Dundee DD1 9SY, UK; 3 Department of Medical Imaging, Al-Amiri Hospital, Ministry of Health, Sulaibikhat, Kuwait City 13001, Kuwait; 4 Tayside Centre for Genomic Analysis, School of Medicine, University of Dundee, Dundee DD1 9SY, UK; a.z.veluchamy@dundee.ac.uk; 5 Department of Clinical Radiology, Sheikh Jaber Al-Ahmad Al-Sabah Hospital, Ministry of Health, Sulaibikhat, Kuwait City 13001, Kuwait; 6 Department of Pathology, Ninewells Hospital, Dundee DD9 1SY, UK; neil.kernohan@nhs.scot; 7 Surgical Skills Centre, Dundee Institute for Healthcare Simulation Respiratory Medicine and Gastroenterology, School of Medicine, Ninewells Hospital and Medical School, University of Dundee, Dundee DD1 9SY, UK; 8 Division of Population Pharmacogenetics, Population Health and Genomics, Biomedical Research Centre, Ninewells Hospital and Medical School, University of Dundee, Dundee DD1 9SY, UK; c.n.a.palmer@dundee.ac.uk; 9 Division of Cancer Research, School of Medicine, Ninewells Hospital, University of Dundee, Dundee DD1 9SY, UK

**Keywords:** chromophobe, oncocytoma, radiogenomics, computed tomography, renal masses

## Abstract

RO and ChRCC are kidney tumours with overlapping characteristics, making differentiation between them challenging. The objective of this research is to create a radiogenomics map by correlating radiomic features to molecular phenotypes in ChRCC and RO, using resection as the gold standard. Fourteen patients (6 RO and 8 ChRCC) were included in the prospective study. A total of 1,875 radiomic features were extracted from CT scans, alongside 632 cytobands containing 16,303 genes from the genomic data. Feature selection algorithms applied to the radiomic features resulted in 13 key features. From the genomic data, 24 cytobands highly correlated with histology were selected and cross-correlated with the radiomic features. The analysis identified four radiomic features that were strongly associated with seven genomic features. These findings demonstrate the potential of integrating radiomic and genomic data to enhance the differential diagnosis of RO and ChRCC, paving the way for more precise and non-invasive diagnostic tools in clinical practice.

## 1. Introduction

Renal oncocytomas (ROs) and chromophobe renal cell carcinomas (ChRCCs) are two types of renal neoplasms that often present a significant diagnostic challenge due to their overlapping clinical and radiological characteristics [1]. Accurate differentiation between these tumours is essential for appropriate patient management and therapeutic decision-making. However, traditional diagnostic methods, including imaging and histopathology, sometimes fail to reliably distinguish between these entities [2,3]. This highlights the need for more precise and advanced diagnostic techniques.

Radiogenomics, an emerging field that combines radiomics and genomics, holds promise for enhancing the precision of tumour characterisation by correlating imaging features with genetic data [4]. Radiomics involves the extraction of a large number of quantitative features from medical images, transforming them into high-dimensional data that can reveal subtle imaging characteristics not discernible to the naked eye [5]. These data can provide valuable insights into tumour biology and behaviour.

Simultaneously, genomic technologies, particularly SNP-based microarray analysis, have advanced our understanding of the genetic landscape of various cancers. Single Nucleotide Polymorphisms are the most common type of genetic variation and their analysis can uncover critical information about tumour genetics. Specifically, chromosomal copy number variation analysis through SNP-based microarrays can provide a comprehensive genomic profile of tumours, revealing gains and losses in chromosomal regions that are associated with different tumour types [6]. SNP-based CNVs offers a high-resolution view of copy number variations, which are well documented [7,8] in cancer pathogenesis and are often critical in distinguishing benign from malignant tumour behaviours. SNP-based microarray technology enables the detection of CNVs at a much finer scale than traditional karyotyping and FISH [9], allowing identification of smaller genetic changes that might be missed by older methods. This method also provides genome-wide coverage, analysing the entire genome at once and offering a comprehensive view of CNVs across all chromosomes. Unlike whole-genome sequencing (WGS), SNP-based CNV analysis focuses on known variant regions, making it a targeted and clinically relevant approach that is also more accessible in many clinical settings [10,11]. Furthermore, SNP arrays have the unique ability to detect both CNVs and single nucleotide polymorphisms, yielding valuable genetic information relevant to a variety of clinical applications.

Copy number variation represents losses of material when an individual has less than two copies and gains of material when an individual has more than the two expected copies. Additionally, CNVs can involve the heterozygous deletion of one allele or duplication of a maternal or paternal chromosome or a chromosomal region and concurrent loss of the other allele as shown in Figure S1. The genotyping of CNVs from SNP arrays is based on the analysis of the B allele frequency (BAF), which is a measure of heterozygosity, and the log R Ratio (LRR) value, which is a normalised measure of DNA content. LRR is the logged ratio of observed probe intensity to expected intensity, with any deviations from zero in this metric indicating evidence for copy number change. BAF represents the proportion of a hybridised sample that carries the B allele as designated by the Infinium assay. In a normal sample, discrete BAFs of 0.0, 0.5, and 1.0 are expected for each locus, representing AA, AB, and BB genotypes. BAF values are typically used to normalise signal intensity, cleaning poor signals and representing either 0 or 1 for homozygous probes and 0.5 for heterozygous probes. The log R Ratio value is used to detect CNV regions and is generally averaged at zero [12,13].

The goal of radiogenomics is to establish a robust connection between tumour imaging phenotypes and molecular markers, offering a non-invasive alternative to traditional genomic analysis. By using imaging signatures in place of genomic signatures, which typically require invasive tissue sampling, this research aims to provide a less invasive means of assessing genomic characteristics. Additionally, these relationships could help identify patient groups who may benefit from further genomic analysis. Whereas there is a plethora of research on radiogenomics [14,15,16], none of it has investigated the application of radiogenomics in the distinction of ChRCC and RO. Integrating radiomics with SNP-based microarray CNV analysis, this study seeks to develop a comprehensive diagnostic approach that improves the accuracy in differentiating these two clinically challenging renal neoplasms.

In this study, we explored the promise of radiogenomics as a non-invasive, cost-effective diagnostic approach, especially valuable in distinguishing between renal oncocytoma and chromophobe renal cell carcinoma—tumour types that are challenging to differentiate through imaging alone. By integrating radiomic features with SNP-based CNV data, our goal is to identify imaging biomarkers that correlate with specific genetic variations, potentially enhancing diagnostic accuracy while reducing the need for invasive biopsies. This approach could streamline patient management, particularly in cases where biopsy results are inconclusive or inaccessible, thus supporting more accurate, non-invasive diagnosis and more personalised treatment strategies.

## 2. Results

### 2.1. Statistical Analysis

Statistical analysis was conducted on patients’ age, tumour size, and gender. A statistically significant difference was considered at a 0.05 significance level. From the analysis, it was found that there was no significant difference between tumour size (*p* = 0.791), gender (*p* = 1), or age (*p* = 0.653) and histopathology. Detailed results are shown in Table 1 below. The inter-reader and intra-reader [17] agreement for the segmentations was assessed using the Dice similarity coefficient, resulting in scores of 0.93 and 0.89, respectively, indicating strong agreement for tumour segmentation.

### 2.2. Radiomics Feature Extraction and Selection

**Figure 1:**
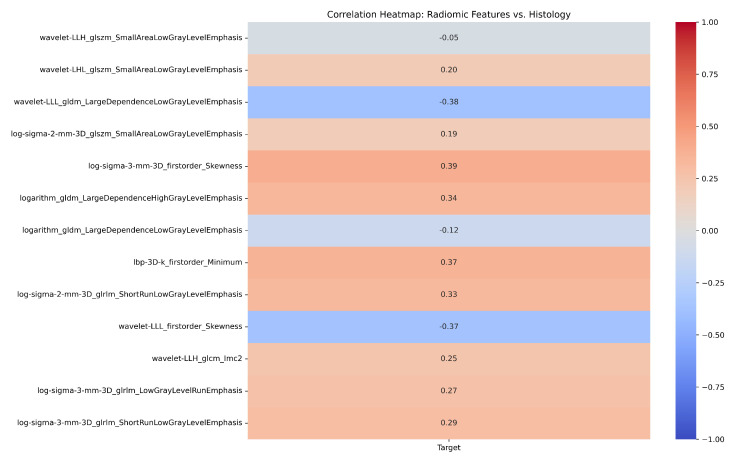
Representation of the correlation between the 13 radiomic features and the histological target.

**Figure 2:**
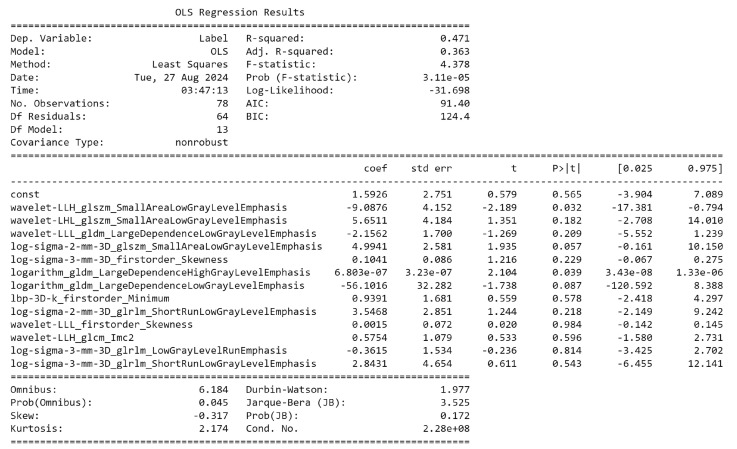
Representation of the OLS regression analysis of radiomic features from 78 patients.

A total of 1875 features were extracted from the CT scan images. LASSO, RFE, XGBoost, and RF each selected the top 14 features from the original feature set as shown in Figures S4–S7. Thirteen features were found to be shared by at least two feature selection algorithms as shown in Figure S2 and Table S3 and were therefore included in the final feature set. Figure 1 and Table S3 highlight the correlation heat map of these 13 features with the histology target. Additionally, Figure S8 presents a heat map showing the correlation between the features and histopathology target per patient. Figure S9 displays a radar plot showing the radiomics feature profiles for each patient, highlighting patterns that correlate with specific histopathological findings. Figure 2 represents OLS regression analysis of radiomic features from 78 patients [17], showing the coefficients, standard errors, t-values, and *p*-values for 13 selected features. Several features, including ‘Wavelet LLH GLSZM Small Area Low Gray-Level Emphasis’ (*p* = 0.032), ‘Logarithm GLDM Large Dependence High Gray-Level Emphasis’ (*p* = 0.039), and ‘Log Sigma 2 mm 3D GLSZM Small Area Low Gray-Level Emphasis’ were found to be statistically significant p≤0.05, suggesting their potential predictive value in the analysis.

### 2.3. Genomic Feature Extraction and Selection

**Figure 3:**
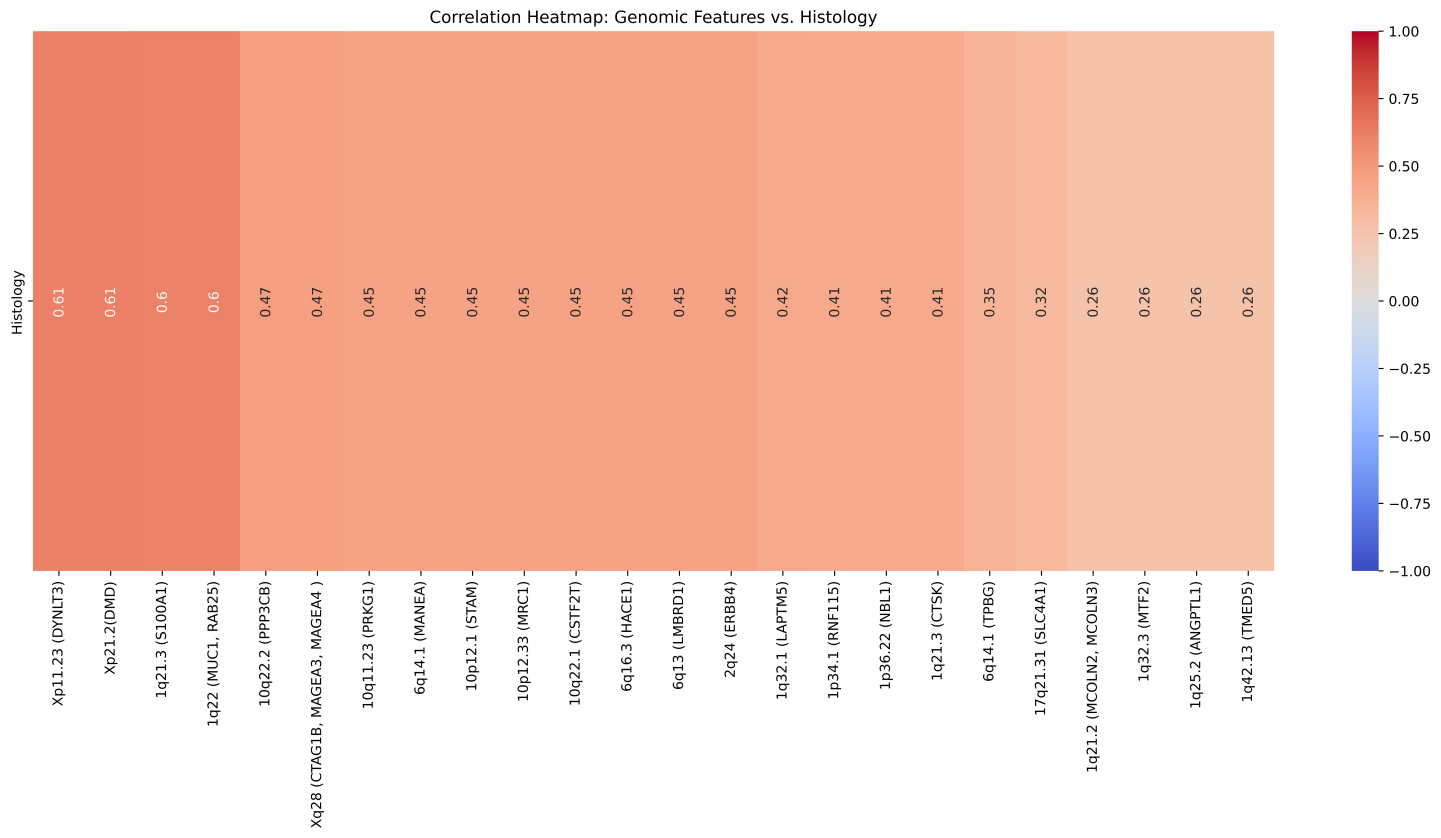
Representation of the correlation between the 24 genomic features and the histological target.

ChXp21.2 (DMD) and ChXp11.23 (DYNLT3) were found to be the most correlated cytobands to histopathology with a correlation of 0.61. Moreover, 22 cytobands were found to be highly correlated with histopathology as highlighted in the heat map in Figure 3 and Table S4.

### 2.4. CNV Analysis

The CNV partition CNV analysis plugin version 3.2.1 and PennCNV version 1.0.5 were used to detect CNVs from the data. The results were compared. The CNV region report generates three separate CNV reports: it lists each CNV and LOH region, estimates allele-specific copy numbers for each probe entry, and creates PLINK CNV input files.

- Standard Report: Lists each copy number variation and loss of heterozygosity (LOH) region for each sample.
- Allele-Specific Copy Number Report: Reports copy number-informed genotypes, such as A- and ABB.
- PLINK CNV Input Report: Creates input files for PLINK CNV Analysis Software plugin version 2.1.4.

**Figure 4:**
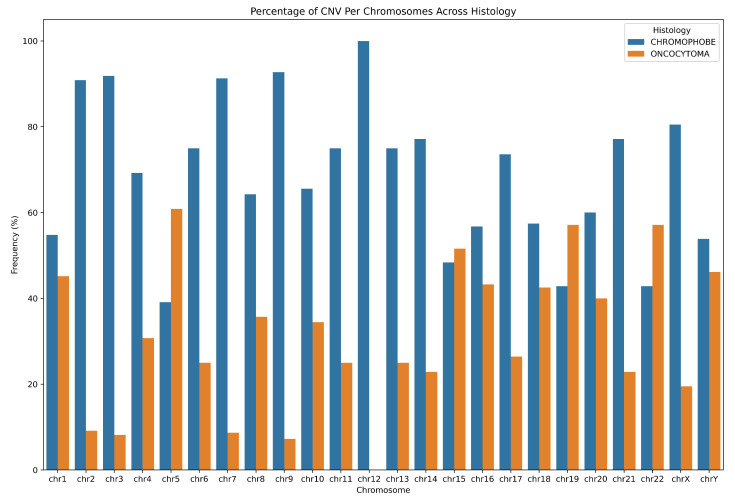
Percentage of CNV per chromosomes across histology.

As mentioned in the Methods section, CNV value and CNV confidence values are presented in the standard report file. For each sample, the type of CNVs as loss (L) or gain (G) or LOH on different cytogenic locations were determined based on the CNV values; refer to Table S2. Based on the quality control criteria and sample types, we selected only CNV data for 14 patients. Table S6 presents the number of amplified, deleted, and LOH segments for each patient, along with the percentage of the genome affected by copy number amplification, loss, and LOH. The percentage of genome copy number variation was calculated using the following equation: CNV% (Duplication (DUP), Deletion (DEL), or Loss of Heterozygosity (LOH)) = (number of segments of a specific type for the patient/total number of segments of that type across all patients) × 100. Additionally, the table provides the mean size of the segments affected by amplification, deletion, and loss of heterozygosity. Figure 4 illustrates the percentage of the CNV value per chromosome across histopathology. Table S7 presents the statistical evidence of chromosomal differences between ChRCC and RO.

### 2.5. Visualisation of Results in Illumina Genome Viewer

**Figure 5:**
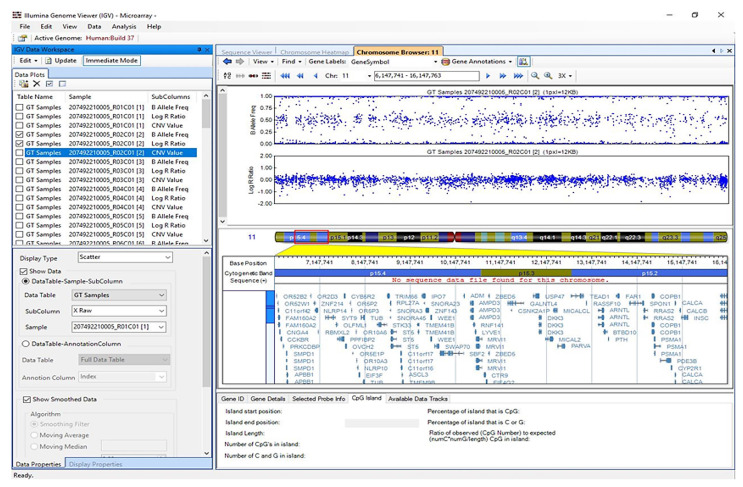
Representation of the result visualisation of the CNV analysis using Illumina Genome Viewer.

**Figure 6:**
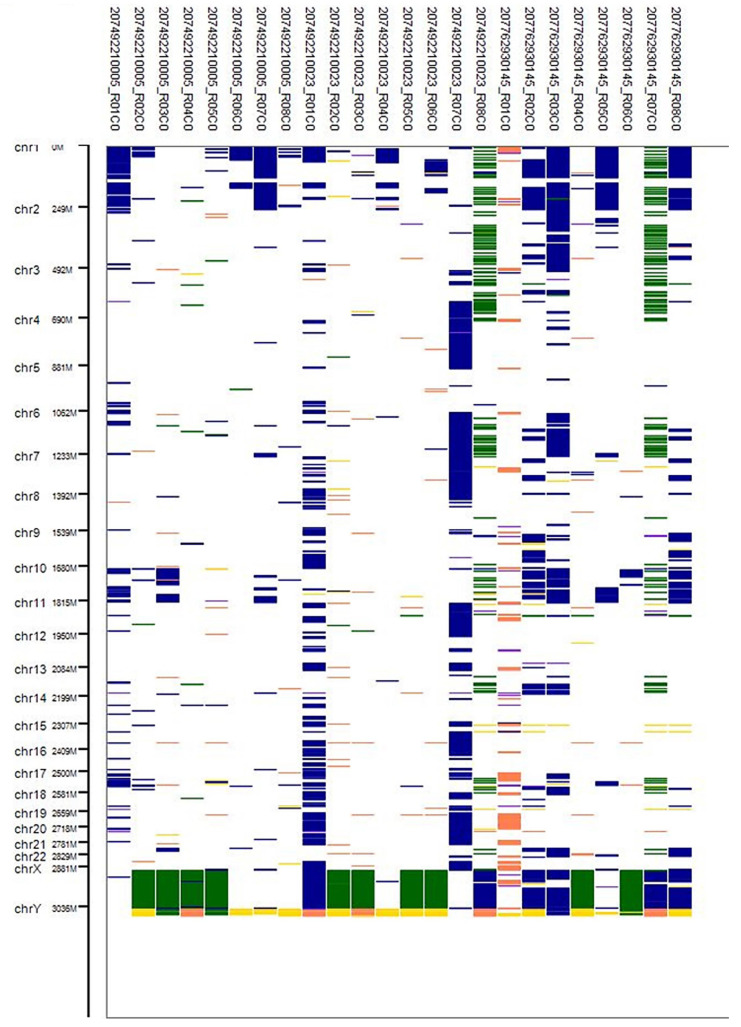
Representation of the CNV regions across all chromosomes in the 24 tissue samples. Dark green for CNV LOH, dark blue and blue violet for gain/duplication, gold and coral for CNV deletion/loss.

Illumina Genome Viewer was used to visualise CNV analysis results. Figure 5 illustrates the B-allele frequency and the normalised intensity, as represented by the Log R ratio, displayed in the context of copy number on the top right panel. Cytogenetic location and gene annotations of a specific sample are displayed on the bottom right panel. In Figure 5, the left panel shows the graphic display of the detected CNV regions across all samples selected for the analysis. Figure 6 represents the CNV regions across all chromosomes.

### 2.6. Classification of CNVs

The numeric pathogenicity score [18], calculated by ClassifyCNV, is converted to pathogenicity classification using the following cut-offs:

- Benign Variant: Scores less than or equal to −0.99.
- Likely Benign Variant: Scores between −0.90 and −0.98.
- Variant of Uncertain Significance: Scores between −0.89 and 0.89.
- Likely Pathogenic Variant: Scores between 0.90 and 0.98.
- Pathogenic Variant: Scores greater than or equal to 0.99.

This classification [18] helps in understanding the potential impact of genetic variants on health, guiding further medical investigation or action. It also includes dosage-sensitive genes contained within the CNV and a list of all protein-coding genes in the CNV. The classification is based on the size of the CNV, gene content, the inheritance pattern, and information in the medical literature and public databases. Table S8 and Figure S10 represent CNV classifications and types identified in ChRCC and RO subtypes.

### 2.7. Radiogenomics Analysis

**Figure 7:**
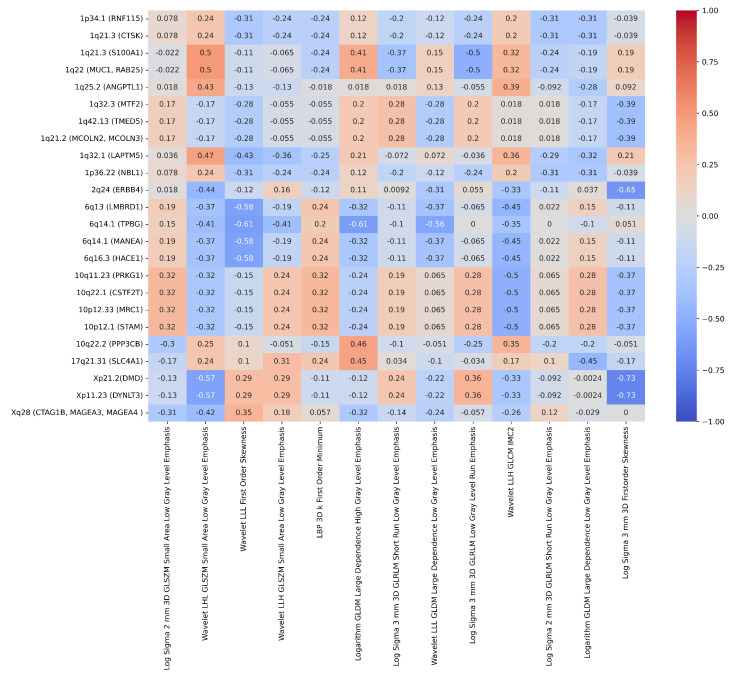
Analysis of the correlation between 13 radiomic and 24 genomic features.

Figure 7 displays the correlation heat map between the 13 radiomic imaging metrics and the 24 molecular genomic features that were highly correlated with the histology target. Table S5 identifies the radiomic and genomic features with a correlation greater than 0.4.

### 2.8. Model Construction

**Figure 8:**
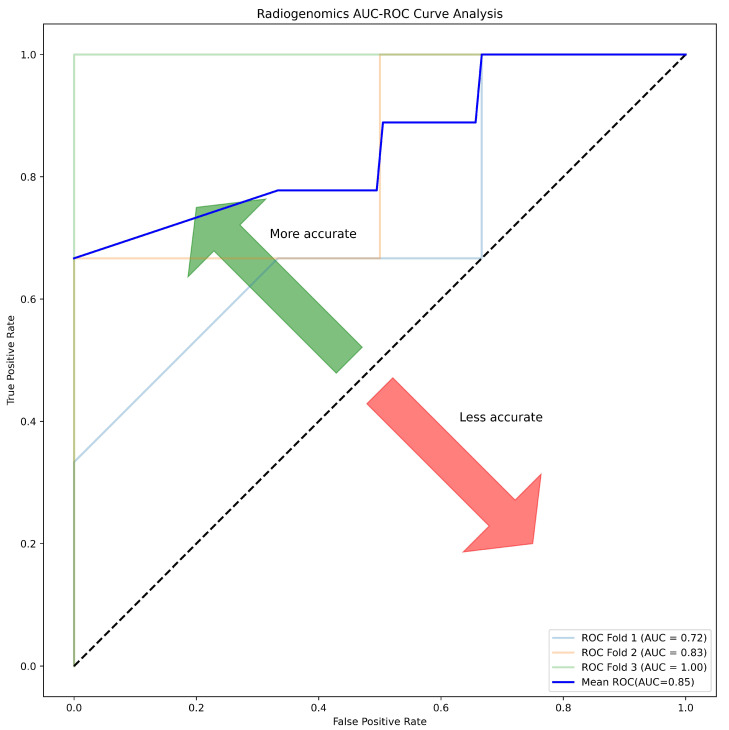
The AUC-ROC for the radiogenomics model with a Pearson’s correlation coefficient (*r*) greater than 0.55 was obtained using the following features: ‘Log Sigma 3 mm 3D Firstorder Skewness’, ‘Logarithm GLDM Large Dependence High Gray-Level Emphasis’, ‘Wavelet LLL Firstorder Skewness’, and ‘Wavelet LHL GLSZM Small Area Low Gray-Level Emphasis’.

The Random Forest (RF) algorithm was constructed using 201 estimators (trees) and different cut-offs for Pearson’s correlation coefficient (*r*), with the results presented in Table S9. The receiver operating characteristic (ROC) curve, illustrating the area under the curve (AUC-ROC) for the radiogenomics model with a correlation (*r*) greater than 0.55, is depicted in Figure 8.

## 3. Discussion

Renal tumours are highly heterogeneous, encompassing at least 16 distinct subtypes, with 4 of these being the most prevalent. Clear cell renal cell carcinoma, which arises from the proximal tubular epithelial cells, is the most prevalent subtype of RCC, representing 70–80% of cases. The next most common subtypes are papillary RCC (10–15%), chromophobe RCC (5%), and collecting duct RCC, which accounts for less than 1% of cases. Renal oncocytomas (ROs) account for approximately 3–7% of all adult renal neoplasms. These tumours are most commonly detected in individuals in their seventh decade of life. In contrast, the incidence of chromophobe renal cell carcinomas (ChRCCs) peaks in the sixth decade. Men are affected by ROs about twice as often as women, whereas ChRCCs generally affect men and women equally [19].

Distinguishing between ROs and ChRCCs is challenging due to their overlapping clinical features. Both types of tumours originate from the intercalated cells of the collecting ducts, which accounts for their histomorphologic, immunophenotypical, ultrastructural, and molecular similarities [20]. Typically, these tumours present as asymptomatic renal masses, often discovered incidentally during imaging performed for unrelated conditions. When symptoms do manifest, they may include weight loss, anorexia, flank pain, palpable masses, haematuria, and non-specific constitutional symptoms [21]. However, these symptoms are more commonly associated with ChRCCs than ROs [19].

The distinction between ChRCC and RO is crucial due to the significant differences in their prognosis and treatment. RO, being a benign tumour with no risk of metastasis, should be identified in advance to avoid unnecessary treatments. In contrast, ChRCC is a malignant tumour with the potential to spread, requiring more intensive treatment, such as surgical resection and constant monitoring. Misdiagnosing these conditions can lead to overtreatment in the case of RO or inadequate treatment in the case of ChRCC, emphasising the importance of accurate differentiation for effective therapeutic decision-making and enhanced patient outcomes.

On CT scans, ChRCC can appear as a well-defined, smooth, homogeneous mass with enhancement patterns similar to those of RO, making differentiation between the two particularly challenging [22,23,24]. Despite advancements in imaging techniques like CT and MRI, both tumours often present with overlapping radiographic features, such as well-circumscribed, homogeneous masses that enhance with contrast, leading to difficulties in distinguishing them [2,19,25]. Moreover, the similar growth rates of these tumours further complicate their clinical differentiation [26,27].

Histopathologically, the similarities between RO and ChRCC are particularly evident, especially with the eosinophilic variant of ChRCC which closely resembles RO. Both tumours can feature large, polygonal cells with eosinophilic cytoplasm, making accurate diagnosis challenging [19]. This overlap necessitates further histochemical and immunohistochemical analyses to distinguish between these two entities accurately, given the malignant potential of ChRCC and the benign nature of RO [28]. The complexity of differentiating these tumours underscores the importance of detailed diagnostic investigations to ensure appropriate treatment strategies.

The human genome exhibits a wide range of genetic structural variations, from large-scale chromosomal abnormalities to single nucleotide polymorphisms (SNPs) [29]. Although SNPs were once considered the primary source of phenotypic variation, recent studies have highlighted the crucial role of copy number variants (CNVs) in contributing to genetic diversity. CNVs, which involve changes in DNA segments longer than 50 base pairs, can affect larger genomic regions, including partial or complete genes, making them a significant component of human genetic diversity [29,30].

Numerous cytogenetic abnormalities are known to exist in cancer cells and genome-wide studies and are therefore used to identify chromosomal aberrations [31]. Accurate detection and clinical annotation of CNVs is essential because they can disrupt genes and regulatory elements and cause benignities or illnesses [18]. They have been connected to a number of hereditary illnesses, including autoimmune diseases, neurodevelopmental problems, and autism spectrum disorders [18,32]. As renal oncocytoma and chromophobe renal cell carcinoma share many characteristics, conventional approaches for distinguishing between the two tumours, such as imaging, histopathological, and genomic techniques frequently lack precision leading to misdiagnosis. Moreover, genomics is usually an expensive venture in cancer diagnosis. Radiogenomics holds the promise for cost-effective cancer diagnosis, particularly in low-resource settings where advanced molecular testing may be prohibitively expensive. By using non-invasive imaging to derive genetic insights, radiogenomics reduces reliance on costly molecular analyses that require specialised facilities and expertise [33]. This approach leverages widely available imaging technologies, such as CT and MRI, to derive molecularly informed diagnostic information, potentially lowering the overall cost of cancer management. Incorporating radiogenomics could thereby increase access to personalised cancer care, enabling healthcare providers in resource-limited settings to offer precise diagnostics without extensive laboratory infrastructure. This financial accessibility can enhance the integration of radiogenomic models into diverse clinical practices, improving patient outcomes and supporting equity in cancer care.

### 3.1. Comparison with Related Methodological Literature

In this study, a correlation of radiomic texture features extracted from computed tomography images and SNP-based microarray copy number variation cytogenomic features was performed. From the findings of the study, it is evident that there is a correlation between radiomic and genomic features, an outcome which has never been found by other studies in the distinction of chromophobe and oncocytoma renal masses. Nonetheless, the research found that the radiogenomics model for features with correlation >0.55 resulted in accuracy, sensitivity, specificity, and AUC of 81.25, 75.00, 87.50 and 85.00, respectively.

The research had 16,303 genes in total, out of which 97 genes were either significantly overlapping between the two tumour subtypes or were majorly present in either of the tumours. These genes were found in 61 different cytobands and were therefore correlated with tumour subtype. On correlation, 24 cytobands containing 28 affected genes were found to be highly correlated to histopathology. These cytobands were found in Chromosomes 1, 2, 6, 10, 17, and X. This is comparable to previous studies [20,34,35,36,37,38,39,40] which found these chromosomes to be associated with either chromophobe or oncocytoma. Specifically, 1p34.1 (RNF115), 1q21.3 (CTSK), 1q21.3 (S100A1), 1q22 (MUC1, RAB25), 1q25.2 (ANGPTL1), 1q32.3 (MTF2), 1q42.13 (TMED5), 1q21.2 (MCOLN2, MCOLN3), 1q32.1 (LAPTM5) and 1p36.22 (NBL1) were all found in Chromosome 1.

RNF115 has emerged as a significant gene in our study, as well as in the broader study of renal tumours, particularly in differentiating between chromophobe renal cell carcinoma (ChRCC) and renal oncocytoma (RO). Research indicates that RNF115 is consistently expressed in all cases of renal oncocytoma and in oncocytic neoplasms favouring oncocytoma, but it is barely detectable in ChRCC [41,42,43,44,45]. The study by Iakymenko et al. [46] investigated the expression of the CTSK gene and its product, Cathepsin K, in RO and ChRCC. The findings revealed that Cathepsin K was positively expressed in both tumour types, with stronger staining observed in renal oncocytoma compared to the weaker, more membranous staining in ChRCC. This expression pattern suggests that while Cathepsin K is present in both types of tumours, the differences in staining intensity might serve as a definitive marker for differentiating between the two. In the study by Li et al. [47], the S100A1 gene was expressed in seven out of eight RO cases, but not in any of the ChRCC cases. This gene expression pattern further supports the use of S100A1 as a diagnostic tool for differentiating between RO and ChRCC. These findings suggest that S100A1 is a useful marker, providing a reliable method for the differential diagnosis of renal RO and ChRCC. The study by Yusenko [48] discussed the expression of the MUC1 gene in the context of differentiating ChRCC from RO. MUC1, also known as epithelial membrane antigen (EMA), showed higher expression levels in ChRCC compared to RO. The study highlighted that while MUC1 is expressed in both tumour types, its stronger and more consistent expression in ChRCC makes it a useful marker in the differential diagnosis between these two renal tumours [48,49]. The findings in our study align with previous research, suggesting that copy number variations (CNVs) in RNF115, CTSK, S100A1, MUC1, RAB25 [50,51], ANGPTL1 [50], MTF2 [52], TMED5 [52], MCOLN2 [50], MCOLN3 [50], LAPTM5 [50], and NBL1 [53,54] could be valuable biomarkers for distinguishing benign RO from malignant ChRCC, which is crucial for accurate diagnosis and treatment planning.

The present research found only a single cytoband in Chromosome 2 containing ERBB4 gene 2q24 (ERBB4). The study by Liu et al. [55] demonstrated that hemizygous deletions of the ERBB4 gene were found in 33% of ChRCC cases, but not in any RO cases, indicating that ERBB4 deletions could serve as a useful marker for distinguishing between these two tumour types. In our study, we found copy number alteration of 2q24 (ERBB4) in 38% of the ChRCC and none in RO, which is comparable to Liu et al. [55].

In a study aimed at distinguishing ChRCC from RO, the genes LMBRD1, TPBG, MANEA, and HACE1 were integral components of a 30-gene signature known as chromophobe and oncocytoma-related gene signature (COGS). These genes were selected based on their differential expression patterns, which were identified through univariate gene expression and ROC curve analyses. The inclusion of these genes in the COGS signature contributed to the study’s ability to achieve a classification accuracy of 97.8% in the discovery dataset and 100% in the validation dataset, effectively differentiating ChRCC from RO using machine learning models. The cytobands 6q13 (LMBRD1), 6q14.1 (TPBG), 6q14.1 (MANEA), and 6q16.3 (HACE1) were found in our study to differentiate RO from ChRCC [50].

According to the study conducted by Yusenko et al. [52], the genes PRKG1 and CSTF2T are located within a region on Chromosome 10q11.23-q21.1, where overlapping alterations were observed in both ChRCC and RO. The study by Krill-Burger et al. [38] found that both ChRCC and RO exhibit significant genomic alterations, including copy number variations, in regions where the MRC1 and STAM genes are located. Specifically, deletions involving MRC1 and STAM were identified in ChRCC, with MRC1 being entirely deleted and STAM partially deleted. These deletions were significant in distinguishing ChRCC from other types, including RO. In our study, 10q11.23 (PRKG1), 10q22.1 (CSTF2T), 10p12.33 (MRC1), and 10p12.1 (STAM) occurred in 37.5% of ChRCC and not in RO, indicating the potential of the two cytobands in differentiating the two tumours.

In the study conducted by Satter et al. [50], PPP3CB was identified as one of the top 197 genes through differential gene expression and receiver-operating characteristic (ROC) analysis. This gene demonstrated a significant area under the curve (AUC) of 0.9 or higher, underscoring its potential role in distinguishing between chromophobe renal cell carcinoma and renal oncocytoma. Similarly, our study identified a copy number variation in the cytoband 10q22.2, which includes PPP3CB, in 33.33% of renal oncocytoma cases, with no such variation observed in chromophobe renal cell carcinoma cases.

The SLC4A1 gene, which encodes for a Solute Carrier Family 4 Member 1, plays a significant role in differentiating between RO and ChRCC. According to the study conducted by Molnar et al. [56], SLC4A1 was expressed in 60% of ROs but only in 11% of ChRCCs. This difference in expression suggests that while SLC4A1 is more commonly associated with ROs, its lower expression in ChRCCs can still be present, albeit less frequently. The findings suggest that SLC4A1 could be used in the differential diagnosis between RO and ChRCC, especially when morphological features overlap [56,57]. In our study, Cytoband 17q21.31 (SLC4A1) occurred in only 16.67% of ROs, which does not provide sufficient proof of its ability to distinguish RO from ChRCC.

In the study by Satter et al. [50], the DMD and DYNLT3 genes are included in the list of 197 top genes identified for their potential to differentiate ChRCC from RO. These genes were selected based on their differential expression and their ability to contribute to a gene signature (COGS) aimed at distinguishing between these two types of renal tumours. In our study, Xp21.2 (DMD) and Xp11.23 (DYNLT3) occurred in 87.5% of ChRCC and 33.33% in RO patients.

The CTAG1B gene, also known as NY-ESO-1, was found to be expressed in 6 out of 18 ChRCCs and 15 out of 17 ROs, suggesting its potential utility in diagnosing these tumours [58]. The study by Demirović et al. [59] investigated the expression of MAGE-A3/4 and NY-ESO-1 in RO and ChRCC, finding significant differences in the expression of these cancer testis antigens between the two tumour types, which may have diagnostic implications. In our study, Xq28 (CTAG1B, MAGEA4, MAGEA3) occurred in 75% of ChRCC and 33.33% RO cases.

In radiomics, a total of 1875 features [60] were initially extracted; however, after applying several feature reduction techniques, this number was reduced to 13 final features. These selected features belong to five radiomic feature classes and four filter classes. Among the final features, two were First-Order features—Skewness and Minimum—each associated with three filter classes: ‘Log Sigma 3 mm 3D’, ‘Wavelet LLL’, and ‘LBP 3D k’. The GLCM class contributed one feature, the ‘Informational Measure of Correlation 2’ (IMC2), which was combined with the ‘Wavelet LLH’ filter. Additionally, two GLDM features were selected: ‘Large Dependence Low Gray-Level Emphasis’ (LDLGLE) and ‘Large Dependence High Gray-Level Emphasis’ (LDHGLE), each combined with the ‘Wavelet LLL’ and Logarithm filters. The GLRLM class included two features: ‘Low Gray-Level Run Emphasis’ (LGLRE) and ‘Short Run Low Gray-Level Emphasis’ (SRLGLE), which were combined with the ‘Log Sigma 3 mm 3D’ and ‘Log Sigma 2 mm 3D’ filters. Finally, one GLSZM feature with three filter types, ‘Log Sigma 2 mm 3D’, ‘Wavelet LHL’, and ‘Wavelet LLH’, was selected.

The ‘Log Sigma 3 mm 3D First-Order Skewness’ and ‘Wavelet LLL First-Order Skewness’ are both radiomic features that measure the asymmetry of the intensity distribution within a 3D medical image [60,61], but they accomplish this using different filtering techniques. The ‘Log Sigma 3 mm 3D First-Order Skewness’ involves applying a ‘3D Gaussian’ smoothing filter with a ‘sigma of 3 mm’, followed by a logarithmic transformation of the image intensities. This process enhances subtle textural details, particularly in lower intensity ranges, and the skewness metric quantifies the asymmetry in the distribution of these intensities. A positive Skewness indicates that the distribution leans towards lower intensity values, while a negative Skewness suggests a bias towards higher intensities. This property is particularly useful in highlighting variations in tissue composition that may be indicative of specific pathologies. On the other hand, the ‘Wavelet LLL First-Order Skewness’ is derived from a different type of filter—the wavelet transform. The ‘Wavelet LLL’ filter applies low-pass filtering across all three dimensions (horizontal, vertical, and diagonal), which smoothens the image and emphasises large-scale, low-frequency components [62]. After this transformation, the Skewness is calculated to assess the asymmetry of the intensity distribution in the filtered image. This feature is effective in capturing broader structural patterns within the tissue, which can be crucial for distinguishing between different types of tissues or abnormalities. In summary, while both features measure Skewness, the ‘Log Sigma 3 mm 3D First-Order Skewness’ focuses on fine details and intensity variations, particularly in lower intensity ranges, and the ‘Wavelet LLL First-Order Skewness’ emphasises larger structural patterns by smoothing the image across multiple dimensions. Both features provide complementary insights into the textural characteristics of tissues, aiding in the differentiation of complex medical conditions like Chromophobe Renal Cell Carcinoma and Renal Oncocytoma. Our findings are similar to what has been highlighted by previous research [63,64,65,66].

‘LBP 3D k First-Order Minimum’ is a combination of Local Binary Patterns (LBPs) in three dimensions with the ’First-Order statistical Minimum’ value. LBP is a texture descriptor that captures the local spatial structure of images by analysing the relationship between a pixel and its surrounding neighbours [67]. When applied in 3D, it extends this analysis to volumetric data, making it highly effective for capturing complex texture patterns in medical images [68,69,70,71,72]. The ‘First-Order Minimum’ aspect focuses on the lowest intensity value in the voxel intensity distribution, providing insight into the darkest or least intense areas within the segmented volume [73]. This combination is particularly useful in radiomics for identifying and characterising subtle variations in texture, which could be indicative of specific tissue properties or pathological conditions.

The radiomic feature of ‘Wavelet LLH GLCM IMC2’ represents a combination of wavelet transformation and Gray-Level Co-occurrence Matrix (GLCM) analysis focused on the ‘Informational Measure of Correlation 2’ (IMC2) [63,65,66,74]. Wavelet transformation is a powerful tool that decomposes an image into different frequency components, allowing for the analysis of various levels of detail [62]. The ‘LLH filter’ specifically applies low-pass filtering in the first two dimensions (L and L) and high-pass filtering in the third dimension (H), capturing the horizontal details within the image. GLCM is a texture analysis method that evaluates the spatial relationship between pixel intensities, and IMC2 is a specific feature derived from GLCM which quantifies the complexity of the texture by measuring the correlation between pixel pairs in the image. High values of IMC2 indicate a more complex and less predictable texture [75,76,77]. By combining these techniques, the ‘Wavelet LLH GLCM IMC2’ feature provides a sophisticated measure of texture that is sensitive to subtle patterns in the image, particularly those related to structural complexity and spatial relationships, making it valuable in distinguishing between ChRCC and RO.

The three radiomic features—’Wavelet LLL GLDM Large Dependence Low Gray-Level Emphasis’, ‘Logarithm GLDM Large Dependence Low Gray-Level Emphasis’, and ‘Logarithm GLDM Large Dependence High Gray-Level Emphasis’—are advanced texture metrics used in radiomic analysis to capture subtle tissue characteristics in medical images [60]. The Gray-Level Dependence Matrix (GLDM) features focus on the relationship between a voxel and its dependent neighbours, emphasising different aspects of texture. ‘Wavelet LLL GLDM Large Dependence Low Gray-Level Emphasis’ is derived from applying a Wavelet transformation with a low-pass filter across all three axes (LLL), which highlights the broader, smooth patterns in the image. The ‘Large Dependence Low Gray-Level Emphasis’ then emphasises regions in the image where large groups of low-intensity pixels are clustered together, capturing homogeneity in low-density areas [78]. ‘Logarithm GLDM Large Dependence Low Gray-Level Emphasis’ is similar to the former but uses a logarithmic transformation instead of a wavelet filter. The logarithm filter can enhance subtle differences in pixel intensity, making this feature particularly useful for detecting fine, low-intensity patterns in the image that might be missed by other filters. ‘Logarithm GLDM Large Dependence High Gray-Level Emphasis’, unlike the previous two, emphasises areas with large clusters of high-intensity pixels. The logarithmic transformation again helps to enhance the contrast and detail within these high-intensity regions, making this feature useful for identifying dense or bright areas within the image that may correlate with certain pathological features [79,80]. Together, these features allow for a detailed analysis of the image’s texture, capturing both low- and high-intensity patterns that can be crucial for distinguishing between different tissue types or identifying specific pathological changes.

The three radiomic features—‘Log Sigma 3 mm 3D GLRLM Low Gray-Level Run Emphasis’, ‘Log Sigma 2 mm 3D GLRLM Short Run Low Gray-Level Emphasis’, and ‘Log Sigma 3 mm 3D GLRLM Short Run Low Gray-Level Emphasis’—are texture measures derived from the Gray-Level Run Length Matrix (GLRLM) combined with specific logarithmic filters applied to 3D images [60]. ‘Log Sigma 3 mm 3D GLRLM Low Gray-Level Run Emphasis’ focuses on the emphasis of runs of low gray-level values, highlighting regions with low-intensity pixels that are clustered together [81]. The ‘Log Sigma 3 mm 3D’ filter applied to this feature enhances finer details within the image at a specific spatial scale, making it useful for identifying subtle low-intensity structures within the volume. ‘Log Sigma 2 mm 3D GLRLM Short Run Low Gray-Level Emphasis’ measures the emphasis on shorter runs of low-intensity pixels, which indicates a texture where these pixels appear in smaller, more isolated clusters. The ‘Log Sigma 2 mm 3D’ filter is used here to capture finer, more localised texture patterns, emphasising the presence of smaller-scale low-intensity areas in the image. ‘Log Sigma 3 mm 3D GLRLM Short Run Low Gray-Level Emphasis’, similarly to the second feature, also emphasises short runs of low gray-level pixels but with a ‘Log Sigma 3 mm 3D filter’. This filter size captures slightly larger texture patterns compared to the 2 mm filter, allowing the feature to identify small but slightly broader low-intensity areas, which could be indicative of certain pathological changes or tissue characteristics. Together, these features provide a nuanced analysis of the texture in medical images, particularly focusing on low-intensity regions, which can be critical for detecting and characterising specific tissue properties or abnormalities [82,83].

The three radiomic features—‘Log Sigma 2 mm 3D GLSZM Small Area Low Gray-Level Emphasis’, ‘Wavelet LHL GLSZM Small Area Low Gray-Level Emphasis’, and ‘Wavelet LLH GLSZM Small Area Low Gray-Level Emphasis’—are derived from the Gray-Level Size Zone Matrix (GLSZM), a texture analysis method that quantifies the size of homogeneous zones of gray levels in an image, combined with specific filters that enhance different aspects of the image texture [60,81]. ‘Log Sigma 2 mm 3D GLSZM Small Area Low Gray-Level Emphasis’ emphasises small areas within the image that consist of low gray-level zones, highlighting regions where small clusters of low-intensity pixels are prevalent. The ‘Log Sigma 2 mm 3D’ filter enhances the detection of fine texture details at a specific spatial scale, making this feature useful for identifying subtle patterns of low-intensity areas in the image. ‘Wavelet LHL GLSZM Small Area Low Gray-Level Emphasis’ [60] is a feature in which the ‘Wavelet LHL’ filter is applied capturing the horizontal high-frequency details along with low-pass filtering in the other directions. This combination focuses on small, low-intensity zones in the image, particularly those with fine horizontal structures, allowing for detailed texture analysis in specific directions. ‘Wavelet LLH GLSZM Small Area Low Gray-Level Emphasis’ is similar to the second feature; it applies the ‘Wavelet LLH’ filter, which emphasises high-frequency details in the vertical direction while applying low-pass filtering horizontally. This feature targets small areas of low-intensity zones, especially those aligned with vertical structures, providing a focused analysis of these specific patterns within the image. These features collectively contribute to a detailed texture analysis by focusing on small, low-intensity areas within the image, enhanced by various filters that capture specific directional details. This facilitates the recognition of fine texture details that could be pivotal in distinguishing various tissue types or pinpointing specific pathological changes in medical imaging [84].

It is worth noting that the radiomic features extracted from the 14 patients did not achieve statistical significance. However, a previous study by Alhussaini et al. [17] involving a larger cohort of 78 patients found that at least four features either attained or approached statistical significance. This finding highlights the vital role that sample size plays in enhancing the statistical power of analyses, demonstrating how a larger sample can reveal significant trends that smaller samples may not capture.

Lichtensztajn et al. [85], demonstrated that the distribution of renal cell carcinoma subtypes varies significantly by race and ethnicity, suggesting that genetic factors play a role in these differences. Similarly, a review by Cotta et al. [86] highlighted the importance of considering genetic diversity in genomic biomarkers for clear cell renal cell carcinoma to improve diagnostic and therapeutic strategies. Genetic variability among racial and ethnic groups may therefore impact the performance of radiogenomic models, as differences in copy number variations and genetic markers for renal tumours have been observed across populations. Studies indicate that kidney tumour biomarkers can vary significantly in frequency and expression across ethnic groups, potentially affecting the sensitivity and specificity of predictive models developed in homogeneous populations. This variability underscores the need for models that account for such diversity to ensure accuracy across diverse patient populations [87,88,89].

In conclusion, our research identified significant correlations between specific radiomic features and genomic markers, highlighting the potential of radiogenomics in non-invasive tumour characterisation. Notably, ‘Log Sigma 3 mm 3D Firstorder Skewness’ showed strong correlations with ChXp21.2 (DMD) (−0.73), ChXp11.23 (DYNLT3) (−0.73), and Ch2q24 (ERBB4) (−0.65). Additionally, ‘Logarithm GLDM Large Dependence High Gray-Level Emphasis’ was linked with Ch6q14.1 (TPBG) (−0.61), while ‘Wavelet LLL Firstorder Skewness’ correlated with Ch6q14.1 (TPBG) (−0.61), Ch6q13 (LMBRD1) (−0.58), Ch6q14.1 (MANEA) (−0.58), and Ch6q16.3 (HACE1) (−0.58). Finally, ‘Wavelet LHL GLSZM Small Area Low Gray-Level Emphasis’ was associated with ChXp21.2 (DMD) (−0.57) and ChXp11.23 (DYNLT3) (−0.57). These findings underscore the potential of radiomic features as surrogates for genomic data, offering promising avenues for enhancing non-invasive diagnostic and prognostic tools in clinical practice.

### 3.2. Limitations and Future Work

In the context of our study, one of the limitations is the relatively small number of subjects, a constraint often encountered in pilot studies. However, our patients reflect routine NHS practice. While this smaller sample size is a common characteristic of preliminary research, it does limit the generalisability of our findings. Despite this, we meticulously detailed all the radiomics and genomics methodologies employed, ensuring that the study is transparent and reproducible.

The limited sample size underscores the need for independent replication of our findings with a larger dataset to validate the results. We calculated the required sample size using the power function:

(1) $$N_{i}=\frac{p_{1}\left(1-p_{1}\right)+p_{2}\left(1-p_{2}\right)}{E^{2}}\;\cdot \;Z^{2}$$

where

- $N_{i}$ is the sample size of each independent sample,
- $p_{1}$ is the proportion of the first sample,
- $p_{2}$ is the proportion of the second independent sample,
- *Z* is the *Z*-score of the confidence interval,
- *E* is the margin of error.

Using this formula, with $p_{1}=0.286$, $p_{2}=0.714$, E=0.05, and Z=1.96, we estimate that a sample size of approximately 1254 subjects is necessary to achieve adequate statistical power; refer to Supplementary Materials Tables S16 and S17. We recognise that with a larger and more diverse cohort, the accuracy of future research is likely to offer better and more consistent results due to a more varied population.

Although we followed standardised imaging protocols, variations in imaging quality, such as resolution and contrast timing differences, could impact the consistency of radiomic feature extraction across different settings. Future studies incorporating multi-centre data from various imaging equipment could address this variability, improving model reliability. Additionally, manual tumour segmentation introduces potential inter-observer differences, even among experienced radiologists. Although we calculated the Dice similarity coefficient to assess segmentation consistency, future work could benefit from using automated segmentation tools to reduce observer-dependent variability further. Moreover, the inherent heterogeneity in tissue samples poses another challenge, as genetic diversity within these samples can influence CNV analysis quality. Ensuring rigorous tissue quality control and using optimised sampling methods would help mitigate the effects of sample heterogeneity on genomic data, increasing the transparency and reliability of the findings.

The conclusions of this study are based on data from a single-site cohort, limiting its applicability to only the United Kingdom population. To enhance the applicability of radiogenomic models across diverse populations, future research should focus on collecting and analysing genomic and imaging data from a wide range of racial and ethnic groups to capture the full spectrum of genetic variability. Additionally, it is essential to validate these models in diverse cohorts to assess their performance and reliability across populations. Promoting interdisciplinary collaboration among geneticists, radiologists, and oncologists is crucial for developing comprehensive models that integrate genetic, imaging, and clinical data. Addressing these areas will advance more inclusive and effective radiogenomic applications, ultimately easing their translation into clinical practice and improving patient outcomes across varied demographic groups.

Likewise, we recommend the use of machine learning with nested cross-validation on the identified features to enhance the robustness and accuracy of such models. Nested cross-validation [90,91,92] is advantageous because it helps prevent over-fitting by incorporating an additional layer of validation. This method is particularly beneficial in tuning hyperparameters while simultaneously assessing model performance, leading to more reliable and generalisable results when applied to new data.

Nested cross-validation is unsuitable for our dataset of only 14 patients due to inherent limitations arising from the small sample size. With so few patients, each fold in the cross-validation process would contain only a handful of samples, resulting in high variability in performance estimates. This small number of samples per fold can lead to unstable and unreliable results, as each split may fail to capture a representative subset of the data. Additionally, models trained on such a limited dataset are highly sensitive to minor variations, which nested cross-validation can exacerbate by further reducing the effective training sample size within each fold. This reduction increases the likelihood of model instability and compromises the reliability of the evaluation while also heightening the risk of overfitting due to insufficient training data in each fold.

In future studies, it is essential to consider the limitations of both CT imaging and SNP microarray techniques used in radiogenomics. CT imaging, while widely available, has limited resolution compared to other imaging modalities, which may hinder the accurate delineation of small anatomical or pathological features [93,94,95]. This limitation can impact the precision of radiomic feature extraction, potentially affecting model performance. Similarly, SNP microarray analysis, while effective for detecting large-scale chromosomal alterations, may lack the sensitivity required to identify smaller genetic changes or complex structural variations [96,97]. To improve the robustness of radiogenomic models, future research should explore integrating higher-resolution imaging modalities and more sensitive genomic techniques, such as next-generation sequencing, to capture subtle genetic and imaging features. Addressing these limitations could enhance the accuracy and applicability of radiogenomic models across diverse clinical contexts.

Acknowledging these limitations, we believe that our study provides valuable insights and a strong foundation for future research. Nonetheless, independent replication with a more extensive dataset is crucial to confirm the robustness and generalisability of our findings.

### 3.3. Strengths

- Non-Invasive Analysis: It is considered to be an alternative to biopsies by leveraging imaging data, which can be obtained non-invasively, reducing the need for tissue biopsies. This is particularly beneficial for patients with tumours in hard-to-reach locations or those who cannot undergo invasive procedures.
- Comprehensive Tumour Profiling: Unlike traditional biopsies, which sample only a small portion of a tumour, radiogenomics analyses the entire tumour through imaging. This provides a more comprehensive view of tumour heterogeneity, capturing variations across different regions of the tumour.
- Molecular Insights from Imaging: Radiogenomics establishes correlations between imaging features and molecular markers, allowing for the prediction of genetic and molecular characteristics based on imaging data. This can lead to better understanding and characterisation of tumours.
- Tailored Treatment Strategies: By linking imaging features with specific genetic mutations, radiogenomics can help in personalising treatment plans. This ensures that therapies are more closely aligned with the molecular profile of the tumour, potentially improving patient outcomes.
- Potential for Early Detection and Prognosis: Radiogenomic research can identify imaging biomarkers that correlate with molecular signatures, which may be used for early detection of diseases or to predict disease outcomes such as response to treatment or risk of recurrence.
- Widespread Imaging Availability: Imaging technologies like CT, MRI, and PET scans are widely available in clinical settings, making radiogenomics more accessible and scalable compared to genetic testing, which may require specialised laboratories and significant costs.
- Cost-Effectiveness: In low-resource settings where molecular testing may be cost-prohibitive, radiogenomics offers a more affordable alternative for tumour characterisation and risk stratification.
- Real-Time Tracking: This approach allows for continuous monitoring of tumour changes over time through serial imaging, enabling the assessment of treatment response and disease progression without repeated invasive procedures.
- Utilisation of Routine Clinical Data: Research can utilise existing imaging data routinely collected in clinical practice, making it possible to conduct large-scale studies without the need for new data collection efforts.
- Facilitating Research and Clinical Trials: These trials can aid in the discovery and validation of new biomarkers, enhancing the design and effectiveness of clinical trials. They also enable the stratification of patients based on imaging-genomic correlations, improving trial outcomes.
- Advancing Precision Medicine: It enhances the precision of medical interventions by integrating imaging and genomic data, leading to more accurate diagnoses, better-targeted therapies, and improved patient management.

### 3.4. Summary

Our radiogenomic study reveals that CT scans can effectively capture changes linked to intrinsic molecular characteristics in rare renal tumour subtypes. This is supported by predictive imaging-based models that showed performance comparable to validated gene signatures for the ChRCC and RO molecular subtypes. These findings suggest that imaging features could serve as accessible, non-invasive surrogates for molecular characteristics, providing a cost-effective alternative to genetic assessment. This is particularly beneficial in low-income settings where molecular testing may be financially prohibitive, thereby promoting more equitable access to personalised cancer care and enhancing clinical decision-making. However, further research is essential to validate and refine these imaging-based signatures across diverse populations and clinical contexts. Future studies should focus on integrating standardised imaging protocols and advanced machine learning techniques to improve the accuracy of these non-invasive biomarkers. Validation by independent researchers is crucial to confirm the robustness and clinical applicability of these findings.

## 4. Materials and Methods

### 4.1. Ethical Approval

This study received approval from the East of Scotland Research Ethical Service. Access to patients’ medical healthcare data was granted under Caldicott Approval Number IGTCAL9519 on 25 August 2021. Additionally, the Tissue Bank Committee [98] approved the application number TR000611 for this study on 29 March 2022.

### 4.2. Patients and Tissues

This research was a prospective study conducted using a database of 35 patients (10 with ChRCC and 25 with RO) from Ninewells Hospital, collected between 2011 and 2021. All cases were pathologically confirmed at the institution. Patients lacking approval from the tissue bank and patients with poor DNA yield were excluded from the study. The participants underwent pre-operative contrast-enhanced CT scan imaging. The imaging data, provided in DICOM format with a resolution of 512 × 512 pixels, was obtained from the institution’s Picture Archiving and Communication System (PACS). Likewise, the patients had various types of tissue samples. These samples included formalin-fixed paraffin-embedded (FFPE) and fresh-frozen tissues, with some patients undergoing biopsies, resections, or both. However, the total number of patients accessible for the study was significantly reduced to 14 due to several obstacles, i.e., 15 patients had poor DNA yield from the biopsy FFPE samples and issues with bead carryover during the DNA extraction process. An additional 6 patients were eliminated due to a lack of approval from the tissue bank. Figure S11 represents the exclusion and inclusion criteria for the study. For the clinical report information of the 35 patients, refer to Tables S10–S14.

As a result of certain patients lacking sufficient tissue samples for analysis, the study’s focus shifted to utilising fresh-frozen tissues and FFPE samples obtained from patients who underwent partial or radical nephrectomy while excluding those who only underwent biopsy. For the next step, namely DNA extraction, we sectioned 72 tissue samples (3 sections per sample). We utilised 19 FFPE samples and 5 fresh-frozen samples, totalling 24 samples collected from 14 patients (6 RO and 8 ChRCC) for this research. The DNA quantification and purity is presented in Table S15. Refer to Supplementary Material Figures S13–S24 and Tables S18–S22 for sample preparation and SNP-based microarrays technical lab details. Figure S12 summaries the study’s methodological process.

### 4.3. Statistical Analysis

A statistical analysis was conducted using the SciPy package in Python to evaluate the relationships between age, tumour size (3D), gender, and histopathology. Additionally, the associations between radiomic features, cytogenetic features, CN size, CN value, and histopathology were investigated. The Chi-square test ($\chi^{2}$) and Student’s T-test (t) were used to assess differences between groups, while Pearson’s correlation coefficient (*r*) was used to measure the strength and direction of the linear relationship between variables. Significance level was set at 0.05, aligning with conventional standards in medical research to balance sensitivity and specificity. This threshold was selected to limit Type I errors while maintaining statistical power.

### 4.4. Computed Tomography Scans

Data were captured using a Helical CT scanner from GE-Healthcare, Chicago, IL, USA. The scanning parameters included a large body scan field of view (SFOV), a gantry rotation time of 0.7 s, a slice thickness of 1.25 mm, a pitch of 1375:1, and a detector coverage of 40 mm. The Noise Index (NI) was set to 30, with a Computed Tomography Dose Index Volume (CTDIvol) of 9.59 mGy. The X-ray tube-voltage was 120 kVp, and the X-ray tube-current ranged from 100 to 560 mA (auto-modulated) depending on patient size. The contrast agent used was intravenous Omnipaque 300 (concentration), administered at 80–100 mL per patient. A Bayer Centargo contrast pressure injector was used, with a flow rate of 3 mL/s for the renal scan. The crucial pre-operative CT nephrographic stage, occurring 100 to 120 s after IV contrast injection, was utilised in this study. This stage, identified by studies [99,100], allows the clearest identification of renal lesions.

### 4.5. Tumour Volume Segmentation Technique

**Figure 9:**
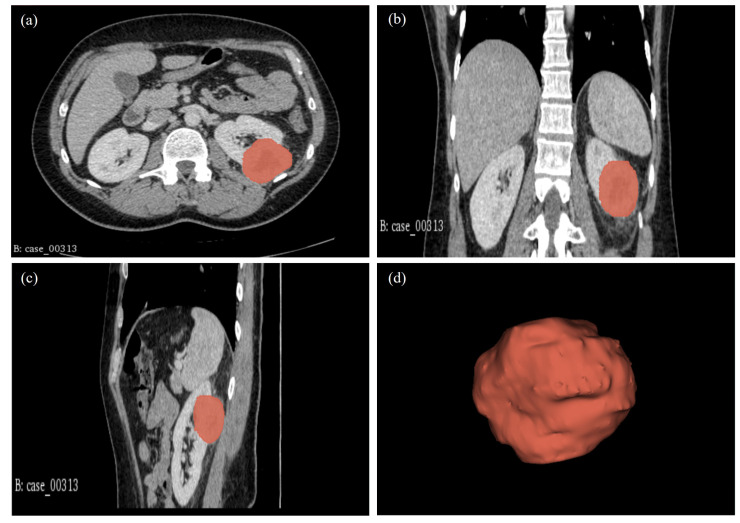
Manual segmentation of the 3D image slices using the 3D Slicer software: version 4.11.20210226. (**a**) CT scan axial plane; (**b**) Coronal plane; (**c**) Sagittal plane; and (**d**) 3D VOI.

CT image slices for each patient were converted to 3D NIFTI (Neuroimaging Informatics Technology Initiative) format using Python version 3.9. These 3D images were then imported into 3D Slicer software, version 4.11.20210226, for segmentation. Manual segmentation was conducted on the 3D images, delineating the edges of the tumour slice by slice to obtain the volume of interest (VOI) as shown in Figure 9.

The procedure was carried out twice by a blinded investigator (A.J.A.) with 14 years of experience in interpreting medical images who was unaware of the tumour’s final pathological grade. Another blinded investigator (A.J.), with 12 years of experience in medical imaging technology, conducted confirmatory segmentation. The inter-reader and intra-reader [17] agreement for the segmentations was determined using the Dice similarity coefficient (DSC). It effectively quantifies the overlap between two segmentations and provides a clear, interpretable measure of similarity, ranging from 0 (no overlap) to 1 (perfect overlap), making it ideal for evaluating the consistency and accuracy of segmentations in medical imaging. DSC robustness to small variations and wide acceptance in the field make it a reliable choice for assessing agreement in this study.

The Dice similarity coefficient (DSC) was calculated using the following formula [101]:

(2) $$\mathrm{DSC}=\frac{2|A\cap B|}{\left|A|+|B\right|}$$

where

- |A∩B| is the size of the intersection between two sets *A* and *B* (i.e., the number of pixels in the case of image segmentation that are common to both sets);
- |A| and |B| are the sizes of sets *A* and *B*, respectively (i.e., the number of pixels in each set).

In the context of image segmentation,

- *A* represents the set of pixels in the segmentation performed by one reader or at one time point;
- *B* represents the set of pixels in the segmentation performed by another reader or at another time point.

Subsequently, the segmentations were evaluated by an independent experienced urological surgical oncologist (G.N.) who considered radiology and histology reports. The gold standard for pathology diagnosis was assumed to be histopathology from partial or radical nephrectomy. The result of the segmentation was a binary mask of the tumour.

### 4.6. Radiomics Feature Computation

Texture descriptors of the features were computed using the PyRadiomics module in Python 3.6.1 [102]. The goal of PyRadiomics is to provide a standardised method for extracting radiomic features from medical images, minimising inter-observer variability [60]. The parameters used in PyRadiomics included a minimum region of interest (ROI) dimension of 2, a pad distance of 5, normalisation set to false, and a normaliser scale of 1. No outliers were removed, no re-sampling of pixel spacing was performed, and no pre-cropping of the image was applied. SitkBSpline was used as the interpolator, with the bin-width set to 20. On average, PyRadiomics generated approximately 1500 features per image, allowing the extraction of seven feature classes from each 3D image. The extracted feature categories included first-order (19 features), grey-level co-occurrence matrix (GLCM) (24 features), grey-level run-length matrix (GLRLM) (16 features), grey-level size-zone matrix (GLSZM) (16 features), grey-level dependence matrix (GLDM) (14 features), neighboring grey-tone difference matrix (NGTDM) (5 features), and 3D shape features (16 features). These features enable the computation of texture intensities and their distribution within the image [60].

In a previous study [17], combining original feature classes with filter features significantly enhanced model performance. Consequently, we extracted filter class features in addition to the original features. These filter classes included local binary pattern (LBP-3D), gradient, exponential, logarithm, square-root, square, Laplacian of Gaussian (LoG), and wavelet. Each filter was applied to every feature in the original feature classes. For example, since the first-order statistic feature class has 19 features, it also includes 19 LBP filter features. The filter class features were named by combining the name of the original feature with the name of the filter class [60].

### 4.7. Radiomics Feature Pre-Processing and Selection

The radiomic features were normalised using a standard scaler so that the mean of each feature was zero, with a standard deviation of one. The ground truth labels were annotated as 1 and 0 for ChRCC (positive class) and RO (negative class), respectively, in preparation for classification. Inter-feature correlation coefficients were computed, and when two features had a correlation coefficient greater than 0.8, one of the features was dropped. Thereafter, the least absolute shrinkage and selection operator (LASSO) model, recursive feature elimination (RFE), extreme gradient boosting (XGBoost), and random forest (RF) were used to select the essential features, as shown in Figure S2. Features selected by any two algorithms were included in the final feature set.

### 4.8. Tissue Data Scanning and Processing

Genomic DNA samples from 14 patients were extracted from formalin-fixed paraffin-embedded (FFPE) and frozen tissue samples. A total of 24 genomic DNA samples were genotyped using Infinium CytoSNP-850K v1.2 BeadChip [103] according to the manufacturer’s instructions. CytoSNP-850K v1.2 BeadChip is used to identify genetic and structural variants. The array contains approximately 850,000 single nucleotide polymorphism (SNP) markers spanning the entire genome, with an average probe spacing of 50 mer oligonucleotides, which covers cytogenomic-relevant genes from the International Collaboration for Clinical Genomics (ICCG) and the Cancer Cytogenomics Microarray Consortium (CCMC) for cancer research applications. It also provides enriched coverage for 3262 dosage-sensitive genes, and the high 15× bead redundancy facilitates the identification of CNV calls with high confidence. Genotyped arrays were processed and scanned using iScan. The raw data were processed, quality assessed, and analysed initially using the Illumina Genome Studio genotyping module [104] based on the reference human genome (hg19/GRCh37). The clustering of intensities for all SNPs was performed using the raw data, the SNP manifest file (.bpm), and the standard cluster file (.egt). Genotyping calls, which involve determining the specific allele (genetic variant) present at each SNP location, were performed using the GenCall algorithm, implemented within the GenTrain clustering algorithm. GenCall is more suitable for the identification of only common SNPs.

### 4.9. CNV Analysis

#### 4.9.1. Performing CNV Analysis Using cnvPartition Algorithm

For CNV calling, llumina provides its own algorithm named “CNV Partition”. This algorithm is used to identify regions of the genome that are aberrant in copy numbers in samples based on intensity and allele frequency data. This tool is part of the Genome Studio platform 2.0 genotyping module, which can be freely downloaded from the Illumina support page [104]. The cnvPartition algorithm estimates copy number by comparing the observed log R ratio (LRR) and B allele frequency (BAF) for each locus, predicting the LRRs and BAFs for different copy number scenarios. This algorithm employs bivariate Gaussian distribution genotype models using LRRs and BAFs for each of 14 different copy number scenarios, as presented in Table S1 [13]. It provides a quick and straightforward overview of the data, boasting high specificity and a positive predictive rate for both deletions and duplications.

The processed data were analysed using cnvPartition 3.2.0 [105]. Additionally, three other algorithms for CNV analysis were employed: CNV Region Report, Homozygosity Detector, and LOH Score. B Allele Frequency and Log R Ratio plots are visualised using the Chromosome Browser within the Illumina Genome Viewer. Figure S3 illustrates the various standard parameter thresholds employed for CNV analysis, including a confidence threshold of 35 and a minimum probe count of 3. A stringent filtering criterion was implemented to exclude poor-quality samples; specifically, samples with a log R ratio standard deviation greater than 0.28 were removed from further analyses to reduce the incidence of false-positive CNVs. Normal regions exhibit a CNV value of 2 and have an empty CNV confidence as represented in Table S2. Markers linked to identified CNVs display values in the CNV confidence column and varying numbers in the CNV value column. Copy-neutral CNVs are indicated by a CNV value of 2, along with values present in the CNV confidence column [106]. cnvPartition is more user-friendly and best suited for routine analysis of common CNVs on Illumina platforms, offering quick and integrated CNV detection.

#### 4.9.2. Performing CNV Analysis Using PennCNV Algorithm

The PennCNV tool was used to detect CNVs from SNP genotyping arrays. The PennCNV algorithm, which employs a Hidden Markov Model, was used to detect both CNVs and copy-neutral LOH (PennCNV: an integrated hidden Markov model designed for high-resolution copy number variation detection in whole-genome SNP genotyping data Genome Research 17:1665-1674, 2007) [107]. The PennCNV algorithm was developed for genome-wide detection of CNVs using Illumina SNP data and is now available as a plug-in to Illumina’s Genome Studio analysis software version 3.2.1. It utilises the log R ratio (total signal intensity) and B allele frequency (allelic intensity ratio). PennCNV [107] is a more powerful and flexible tool, ideal for advanced users working with complex datasets or needing detailed CNV detection. It excels at identifying rare and complex CNVs with greater sensitivity and adaptability.

### 4.10. Classification of CNVs

The detected CNVs were classified as pathogenic, likely pathogenic, benign, or of uncertain significance using the Classify CNV tool. This classification follows established guidelines from the International Standard Cytogenomic Array (ISCA) and the American College of Medical Genetics and Genomics (ACMG) guidelines [18,108]. The ClassifyCNV is a command-line tool that implements the 2019 ACMG guidelines to evaluate the pathogenicity of germline duplications and deletions [18]. The tool uses pre-parsed publicly available databases to calculate a pathogenicity score for each copy-number variant in accordance with the ACMG guidelines. It obtained a set of 17,683 duplications and 20,805 deletions from the nstd102 study in ClinVar (dbVar) [41]. The ClassifyCNV tool accepts a BED file as input, including chromosome, CNV start position, CNV end position, and CNV type (DEL or DUP). A study conducted by Gurbich and Ilinsky [18] illustrates how the ClassifyCNV algorithm works to classify the following CNVs based on pathogenicity scores: Pathogenic, Likely Pathogenic, Variant of Uncertain Significance (VUS), and Benign.

### 4.11. CNV Analysis Report and Data Extraction Using R Package

The Database of Genomic Variants (DGV) [109], the University of California, Santa Cruz (UCSC) Genome Browser [110], Copy Number Variation Explorer (CNVXPLORER) [111], and Copy Number Variation Clinical Viewer (CNV-ClinViewer) [112] were used to extract Cytogenetic location, CNV size and genomic coordinates. All statistical comparisons were performed by Chi-square testing using the R package version 4.3.1 with a significance level of 0.05.

### 4.12. Chromosomal Cytogenetic Band Selection

Different samples and variance types were segregated to obtain a list of common regions between samples and the associated genes with those regions. This achieved by creating three (.bed) files per individual representing deletion, duplication, and loss of heterozygosity from the original CNV data. These (.bed) files were ran through Bedsect [113], an online implementation of bed-tools intersect to find overlaps between the files, i.e., the regions that vary in more than one sample by types of variation. Subsequently, the list of regions was analysed using UCSC’s Table Browser [114] based on the defined regions from Genome Reference Consortium Human Build 37 (GRCh37), the National Center for Biotechnology Information Reference Sequence (NCBI RefSeq), and the complete RefSeq All dataset. The CNV values of the cytogenetic bands selected were correlated with histopathology to obtain the bands having the highest influence on histopathology. All bands with a correlation above 0.1 were retained for further analysis.

### 4.13. Model Construction

The cytogenetic band and the radiomic features which were found to be highly correlated to histopathology were combined and used to train a machine learning algorithm to predict the tumour subtype. A Random Forest (RF) model was implemented using the cross-validation technique; refer to Supplementary Material for more information about the RF model. Accuracy, sensitivity, specificity, AUC, MCC, and F1 were used to evaluate the model performance.

## 5. Conclusions

In conclusion, this pilot study offers important insights into the role of radiogenomics in distinguishing between RO and ChRCC. By examining the relationship between radiomic features and SNP-based microarray copy number variations, our findings suggest that imaging can serve as a viable non-invasive alternative to traditional molecular diagnostics. The observed correlations between specific imaging characteristics and genomic markers highlight the potential of radiogenomics to improve diagnostic precision, especially in contexts where molecular testing is less accessible. These results provide a foundation for future research aimed at validating and enhancing these imaging-based biomarkers, ultimately paving the way for the integration of radiogenomics into clinical practice to better manage renal tumours and improve patient care.

## Figures and Tables

**Table 1:** Statistical demographic characteristics of the patients’ data.

Patients Characteristics				
	Variable	RO	ChRCC	*p*-Value
	Age (Mean ± SD)	63.5 ± 8.67	61.40 ± 7.13	0.653
n=14	tumour Size	3.60 ± 1.47	3.80 ± 1.09	0.791
	Gender			1
	Male	6 (42.86%)	7 (50.0%)	
	Female	0 (0%)	1 (7.14%)	

## Data Availability

The data provided are available on request from the corresponding author. The codes used to reproduce the results can be found on GitHub, upon request, at the following link: https://github.com/abeer2005/Radiogenomics_ChRCC_RO (accessed on: 20 October 2024).

## Supplementary Materials

The following supporting information can be downloaded at: https://www.mdpi.com/article/10.3390/ijms252312512/s1. References [13,98,103,106,115,116,117,118,119,120,121,122,123] are cited in the Supplementary Materials.

## References

[1] Wobker S.E., Williamson S.R. (2017). Modern pathologic diagnosis of renal oncocytoma. J. Kidney Cancer VHL.

[2] Rosenkrantz A.B., Hindman N., Fitzgerald E.F., Niver B.E., Melamed J., Babb J.S. (2010). MRI features of renal oncocytoma and chromophobe renal cell carcinoma. Am. J. Roentgenol..

[3] Wu J., Zhu Q., Zhu W., Chen W., Wang S. (2016). Comparative study of CT appearances in renal oncocytoma and chromophobe renal cell carcinoma. Acta Radiol..

[4] Saha A., Harowicz M.R., Grimm L.J., Kim C.E., Ghate S.V., Walsh R., Mazurowski M.A. (2018). A machine learning approach to radiogenomics of breast cancer: A study of 922 subjects and 529 DCE-MRI features. Br. J. Cancer.

[5] Lambin P., Rios-Velazquez E., Leijenaar R., Carvalho S., Van Stiphout R.G., Granton P., Zegers C.M., Gillies R., Boellard R., Dekker A. (2012). Radiomics: Extracting more information from medical images using advanced feature analysis. Eur. J. Cancer.

[6] Zhao M., Wang Q., Wang Q., Jia P., Zhao Z. (2013). Computational tools for copy number variation (CNV) detection using next-generation sequencing data: Features and perspectives. BMC Bioinform..

[7] Redon R., Ishikawa S., Fitch K.R., Feuk L., Perry G.H., Andrews T.D., Fiegler H., Shapero M.H., Carson A.R., Chen W. (2006). Global variation in copy number in the human genome. Nature.

[8] McCarroll S.A., Kuruvilla F.G., Korn J.M., Cawley S., Nemesh J., Wysoker A., Shapero M.H., de Bakker P.I., Maller J.B., Kirby A. (2008). Integrated detection and population-genetic analysis of SNPs and copy number variation. Nat. Genet..

[9] Berry N.K., Scott R.J., Rowlings P., Enjeti A.K. (2019). Clinical use of SNP-microarrays for the detection of genome-wide changes in haematological malignancies. Crit. Rev. Oncol./Hematol..

[10] CD Genomics The Principles and Workflow of SNP Microarray. https://www.cd-genomics.com/the-principles-and-workflow-of-snp-microarray.html.

[11] CD Genomics Whole Genome SNP Genotyping. https://www.cd-genomics.com/whole-genome-snp-genotyping.html.

[12] Illumina (2024). Interpreting Infinium Assay Data for Whole-Genome Structural Variation. https://www.illumina.com/Documents/products/technotes/technote_cytoanalysis.pdf.

[13] Illumina (2024). DNA Copy Number and Loss of Heterozygosity Analysis Algorithms. https://www.illumina.com/documents/products/technotes/technote_cnv_algorithms.pdf.

[14] Karlo C.A., Di Paolo P.L., Chaim J., Hakimi A.A., Ostrovnaya I., Russo P., Hricak H., Motzer R., Hsieh J.J., Akin O. (2014). Radiogenomics of clear cell renal cell carcinoma: Associations between CT imaging features and mutations. Radiology.

[15] Ferro M., Musi G., Marchioni M., Maggi M., Veccia A., Del Giudice F., Barone B., Crocetto F., Lasorsa F., Antonelli A. (2023). Radiogenomics in renal cancer management—Current evidence and future prospects. Int. J. Mol. Sci..

[16] Posada Calderon L., Eismann L., Reese S.W., Reznik E., Hakimi A.A. (2023). Advances in imaging-based biomarkers in renal cell carcinoma: A critical analysis of the current literature. Cancers.

[17] Alhussaini A.J., Steele J.D., Nabi G. (2022). Comparative Analysis for the Distinction of Chromophobe Renal Cell Carcinoma from Renal Oncocytoma in Computed Tomography Imaging Using Machine Learning Radiomics Analysis. Cancers.

[18] Gurbich T.A., Ilinsky V.V. (2020). ClassifyCNV: A tool for clinical annotation of copy-number variants. Sci. Rep..

[19] Ng K.L., Rajandram R., Morais C., Yap N.Y., Samaratunga H., Gobe G.C., Wood S.T. (2014). Differentiation of oncocytoma from chromophobe renal cell carcinoma (RCC): Can novel molecular biomarkers help solve an old problem?. J. Clin. Pathol..

[20] Dvorakova M., Dhir R., Bastacky S.I., Cieply K.M., Acquafondata M.B., Sherer C.R., Mercuri T.L., Parwani A.V. (2010). Renal oncocytoma: A comparative clinicopathologic study and fluorescent in-situ hybridization analysis of 73 cases with long-term follow-up. Diagn. Pathol..

[21] Vera-Badillo F.E., Conde E., Duran I. (2012). Chromophobe renal cell carcinoma: A review of an uncommon entity. Int. J. Urol..

[22] Kim J.K., Kim T.K., Ahn H.J., Kim C.S., Kim K.R., Cho K.S. (2002). Differentiation of subtypes of renal cell carcinoma on helical CT scans. Am. J. Roentgenol..

[23] Choudhary S., Rajesh A., Mayer N., Mulcahy K., Haroon A. (2009). Renal oncocytoma: CT features cannot reliably distinguish oncocytoma from other renal neoplasms. Clin. Radiol..

[24] Bird V.G., Kanagarajah P., Morillo G., Caruso D.J., Ayyathurai R., Leveillee R., Jorda M. (2011). Differentiation of oncocytoma and renal cell carcinoma in small renal masses (<4 cm): The role of 4-phase computerized tomography. World J. Urol..

[25] Akın I.B., Altay C., Güler E., Çamlıdağ İ., Harman M., Danacı M., Tuna B., Yörükoğlu K., Seçil M. (2019). Discrimination of oncocytoma and chromophobe renal cell carcinoma using MRI. Diagn. Interv. Radiol..

[26] Kurup A.N., Thompson R.H., Leibovich B.C., Harmsen W.S., Sebo T.J., Callstrom M.R., Kawashima A., Atwell T.D. (2012). Renal oncocytoma growth rates before intervention. BJU Int..

[27] Chawla S.N., Crispen P.L., Hanlon A.L., Greenberg R.E., Chen D.Y., Uzzo R.G. (2006). The natural history of observed enhancing renal masses: Meta-analysis and review of the world literature. J. Urol..

[28] Baharzadeh F., Sadeghi M., Ramezani M. (2019). Chromophobe renal cell carcinoma or oncocytoma: A manner of challenge in frozen section diagnosis. BioMedicine.

[29] Shao X., Lv N., Liao J., Long J., Xue R., Ai N., Xu D., Fan X. (2019). Copy number variation is highly correlated with differential gene expression: A pan-cancer study. BMC Med. Genet..

[30] Sebat J., Lakshmi B., Troge J., Alexander J., Young J., Lundin P., Manér S., Massa H., Walker M., Chi M. (2004). Large-scale copy number polymorphism in the human genome. Science.

[31] Albertson D.G., Pinkel D. (2003). Genomic microarrays in human genetic disease and cancer. Hum. Mol. Genet..

[32] Shaikh T.H. (2017). Copy number variation disorders. Curr. Genet. Med. Rep..

[33] National Human Genome Research Institute (2023). The Cost of Sequencing a Human Genome. https://www.genome.gov/about-genomics/fact-sheets/Sequencing-Human-Genome-cost.

[34] Füzesi L., Frank D., Nguyen C., Ringert R.H., Bartels H., Gunawan B. (2005). Losses of 1p and chromosome 14 in renal oncocytomas. Cancer Genet. Cytogenet..

[35] Yap N.Y., Rajandram R., Ng K.L., Pailoor J., Fadzli A., Gobe G.C. (2015). Genetic and chromosomal aberrations and their clinical significance in renal neoplasms. BioMed Res. Int..

[36] Ohashi R., Schraml P., Angori S., Batavia A.A., Rupp N.J., Ohe C., Otsuki Y., Kawasaki T., Kobayashi H., Kobayashi K. (2019). Classic chromophobe renal cell carcinoma incur a larger number of chromosomal losses than seen in the eosinophilic subtype. Cancers.

[37] Tan M.H., Wong C.F., Tan H.L., Yang X.J., Ditlev J., Matsuda D., Khoo S.K., Sugimura J., Fujioka T., Furge K.A. (2010). Genomic expression and single-nucleotide polymorphism profiling discriminates chromophobe renal cell carcinoma and oncocytoma. BMC Cancer.

[38] Krill-Burger J.M., Lyons M.A., Kelly L.A., Sciulli C.M., Petrosko P., Chandran U.R., Kubal M.D., Bastacky S.I., Parwani A.V., Dhir R. (2012). Renal cell neoplasms contain shared tumor type–specific copy number variations. Am. J. Pathol..

[39] Van den Berg E., Van der Hout A., Oosterhuis J., Störkel S., Dijkhuizen T., Dam A., Zweers H., Mensink H., Buys C., De Jong B. (1993). Cytogenetic analysis of epithelial renal-cell tumors: Relationship with a new histopathological classification. Int. J. Cancer.

[40] Herbers J., Schullerus D., Chudek J., Bugert P., Kanamaru H., Zeisler J., Ljungberg B., Akhtar M., Kovacs G. (1998). Lack of genetic changes at specific genomic sites separates renal oncocytomas from renal cell carcinomas. J. Pathol. J. Pathol. Soc. Great Br. Irel..

[41] National Center for Biotechnology Information (NCBI) (2024). National Center for Biotechnology Information (NCBI)-nstd102—Clinical Structural Variants. https://www.ncbi.nlm.nih.gov/dbvar/studies/nstd102.

[42] Wang M.X., Liuyu T., Zhang Z.d. (2022). Multifaceted roles of the E3 ubiquitin ligase RING finger protein 115 in immunity and diseases. Front. Immunol..

[43] Amemiya Y., Bacopulos S., Seth A. (2014). Novel Ubiquitin E3 Ligases as Targets for Cancer Therapy: Focus on Breast Cancer-Associated Gene 2 (BCA2). Resistance to Proteasome Inhibitors in Cancer: Molecular Mechanisms and Strategies to Overcome Resistance.

[44] Pan Z. (2016). Identification of Novel Substrates of the Ubiquitin E3 Ligase RNF126 and Characterization of Its Role in Lipid Droplet Homeostasis. Master’s Thesis.

[45] Ehsani L., Seth R., Bacopulos S., Seth A., Osunkoya A.O. (2013). BCA2 is differentially expressed in renal oncocytoma: An analysis of 158 renal neoplasms. Tumor Biol..

[46] Iakymenko O.A., Delma K.S., Jorda M., Kryvenko O.N. (2021). Cathepsin K (clone EPR19992) demonstrates uniformly positive immunoreactivity in renal oncocytoma, chromophobe renal cell carcinoma, and distal tubules. Int. J. Surg. Pathol..

[47] Li G., Gentil-Perret A., Lambert C., Genin C., Tostain J. (2005). S100A1 and KIT gene expressions in common subtypes of renal tumours. Eur. J. Surg. Oncol. (EJSO).

[48] Yusenko M.V. (2010). Molecular pathology of chromophobe renal cell carcinoma: A review. Int. J. Urol..

[49] Zhu B., Rohan S.M., Lin X. (2020). Cytomorphology, immunoprofile, and management of renal oncocytic neoplasms. Cancer Cytopathol..

[50] Satter K.B., Tran P.M.H., Tran L.K.H., Ramsey Z., Pinkerton K., Bai S., Savage N.M., Kavuri S., Terris M.K., She J.X. (2022). Oncocytoma-related gene signature to differentiate chromophobe renal cancer and oncocytoma using machine learning. Cells.

[51] Wu H., Fan L., Liu H., Guan B., Hu B., Liu F., Hocher B., Yin L. (2020). Identification of key genes and prognostic analysis between chromophobe renal cell carcinoma and renal oncocytoma by bioinformatic analysis. BioMed Res. Int..

[52] Yusenko M.V., Kuiper R.P., Boethe T., Ljungberg B., van Kessel A.G., Kovacs G. (2009). High-resolution DNA copy number and gene expression analyses distinguish chromophobe renal cell carcinomas and renal oncocytomas. BMC Cancer.

[53] McGillivray P.D., Ueno D., Pooli A., Mendhiratta N., Syed J.S., Nguyen K.A., Schulam P.G., Humphrey P.A., Adeniran A.J., Boutros P.C. (2021). Distinguishing benign renal tumors with an oncocytic gene expression (ONEX) classifier. Eur. Urol..

[54] Rohan S., Tu J.J., Kao J., Mukherjee P., Campagne F., Zhou X.K., Hyjek E., Alonso M.A., Chen Y.T. (2006). Gene expression profiling separates chromophobe renal cell carcinoma from oncocytoma and identifies vesicular transport and cell junction proteins as differentially expressed genes. Clin. Cancer Res..

[55] Liu Q., Cornejo K.M., Cheng L., Hutchinson L., Wang M., Zhang S., Tomaszewicz K., Cosar E.F., Woda B.A., Jiang Z. (2018). Next-generation sequencing to detect deletion of RB1 and ERBB4 genes in chromophobe renal cell carcinoma: A potential role in distinguishing chromophobe renal cell carcinoma from renal oncocytoma. Am. J. Pathol..

[56] Molnar A., Horvath C.A., Czovek P., Szanto A., Kovacs G. (2019). FOXI1 immunohistochemistry differentiates benign renal oncocytoma from malignant chromophobe renal cell carcinoma. Anticancer. Res..

[57] Ishihara H., Yamashita S., Liu Y.Y., Hattori N., El-Omar O., Ikeda T., Fukuda H., Yoshida K., Takagi T., Taneda S. (2020). Genetic and epigenetic profiling indicates the proximal tubule origin of renal cancers in end-stage renal disease. Cancer Sci..

[58] Giesen E., Jilaveanu L.B., Parisi F., Kluger Y., Camp R.L., Kluger H.M. (2014). NY-ESO-1 as a potential immunotherapeutic target in renal cell carcinoma. Oncotarget.

[59] Demirović A., Džombeta T., Tomas D., Spajić B., Pavić I., Hudolin T., Milošević M., Čupić H., Krušlin B. (2010). Immunohistochemical expression of tumor antigens MAGE-A3/4 and NY-ESO-1 in renal oncocytoma and chromophobe renal cell carcinoma. Pathol. Res. Pract..

[60] Van Griethuysen J.J., Fedorov A., Parmar C., Hosny A., Aucoin N., Narayan V., Beets-Tan R.G., Fillion-Robin J.C., Pieper S., Aerts H.J. (2017). Computational radiomics system to decode the radiographic phenotype. Cancer Res..

[61] Coppola F., Mottola M., Lo Monaco S., Cattabriga A., Cocozza M.A., Yuan J.C., De Benedittis C., Cuicchi D., Guido A., Rojas Llimpe F.L. (2021). The heterogeneity of skewness in T2W-based radiomics predicts the response to neoadjuvant chemoradiotherapy in locally advanced rectal cancer. Diagnostics.

[62] Çinarer G., Emiroğlu B.G., Yurttakal A.H. (2020). Prediction of glioma grades using deep learning with wavelet radiomic features. Appl. Sci..

[63] Belfiore M.P., Sansone M., Monti R., Marrone S., Fusco R., Nardone V., Grassi R., Reginelli A. (2022). Robustness of radiomics in pre-surgical computer tomography of non-small-cell lung cancer. J. Pers. Med..

[64] Foy J.J., Robinson K.R., Li H., Giger M.L., Al-Hallaq H., Armato S.G. (2018). Variation in algorithm implementation across radiomics software. J. Med. Imaging.

[65] Linsalata S., Borgheresi R., Marfisi D., Barca P., Sainato A., Paiar F., Neri E., Traino A.C., Giannelli M. (2022). Radiomics of patients with locally advanced rectal cancer: Effect of preprocessing on features estimation from computed tomography imaging. BioMed Res. Int..

[66] Yu H., Scalera J., Khalid M., Touret A.S., Bloch N., Li B., Qureshi M.M., Soto J.A., Anderson S.W. (2017). Texture analysis as a radiomic marker for differentiating renal tumors. Abdom. Radiol..

[67] Rahim M.A., Hossain M.N., Wahid T., Azam M.S. (2013). Face recognition using local binary patterns (LBP). Glob. J. Comput. Sci. Technol..

[68] D’Amico N.C., Sicilia R., Cordelli E., Tronchin L., Greco C., Fiore M., Carnevale A., Iannello G., Ramella S., Soda P. (2020). Radiomics-based prediction of overall survival in lung cancer using different volumes-of-interest. Appl. Sci..

[69] Santucci D., Faiella E., Cordelli E., Sicilia R., de Felice C., Zobel B.B., Iannello G., Soda P. (2021). 3T MRI-radiomic approach to predict for lymph node status in breast cancer patients. Cancers.

[70] Sicilia R., Cordelli E., Merone M., Luperto E., Papalia R., Iannello G., Soda P. Early radiomic experiences in classifying prostate cancer aggressiveness using 3D local binary patterns. Proceedings of the 2019 IEEE 32nd International Symposium on Computer-Based Medical Systems (CBMS).

[71] Tibermacine H., Rouanet P., Sbarra M., Forghani R., Reinhold C., Nougaret S., GRECCAR Study Group (2021). Radiomics modelling in rectal cancer to predict disease-free survival: Evaluation of different approaches. Br. J. Surg..

[72] Yu Y., Li X., Du T., Rahaman M., Grzegorzek M.J., Li C., Sun H. (2024). Increasing the accuracy and reproducibility of positron emission tomography radiomics for predicting pelvic lymph node metastasis in patients with cervical cancer using 3D local binary pattern-based texture features. Intell. Med..

[73] Jensen L.J., Kim D., Elgeti T., Steffen I.G., Schaafs L.A., Hamm B., Nagel S.N. (2022). Enhancing the stability of CT radiomics across different volume of interest sizes using parametric feature maps: A phantom study. Eur. Radiol. Exp..

[74] Scalco E., Belfatto A., Mastropietro A., Rancati T., Avuzzi B., Messina A., Valdagni R., Rizzo G. (2020). T2w-MRI signal normalization affects radiomics features reproducibility. Med. Phys..

[75] Tietz E., Truhn D., Müller-Franzes G., Berres M.L., Hamesch K., Lang S.A., Kuhl C.K., Bruners P., Schulze-Hagen M. (2021). A radiomics approach to predict the emergence of new hepatocellular carcinoma in computed tomography for high-risk patients with liver cirrhosis. Diagnostics.

[76] Shin J., Lim J.S., Huh Y.M., Kim J.H., Hyung W.J., Chung J.J., Han K., Kim S. (2021). A radiomics-based model for predicting prognosis of locally advanced gastric cancer in the preoperative setting. Sci. Rep..

[77] Bernatowicz K., Grussu F., Ligero M., Garcia A., Delgado E., Perez-Lopez R. (2021). Robust imaging habitat computation using voxel-wise radiomics features. Sci. Rep..

[78] Choi W., Liu C.J., Alam S.R., Oh J.H., Vaghjiani R., Humm J., Weber W., Adusumilli P.S., Deasy J.O., Lu W. (2023). Preoperative 18F-FDG PET/CT and CT radiomics for identifying aggressive histopathological subtypes in early stage lung adenocarcinoma. Comput. Struct. Biotechnol. J..

[79] Lee J., Yoo S.K., Kim K., Lee B.M., Park V.Y., Kim J.S., Kim Y.B. (2023). Machine learning-based radiomics models for prediction of locoregional recurrence in patients with breast cancer. Oncol. Lett..

[80] Chen Q., Wang L., Wang L., Deng Z., Zhang J., Zhu Y. (2020). Glioma grade prediction using wavelet scattering-based radiomics. IEEE Access.

[81] Meijer K. (2019). Accuracy and Stability of Radiomic Features for Characterising Tumour Heterogeneity Using Multimodality Imaging: A Phantom Study. Master’s Thesis.

[82] Ericsson-Szecsenyi R., Zhang G., Redler G., Feygelman V., Rosenberg S., Latifi K., Ceberg C., Moros E.G. (2022). Robustness assessment of images from a 0.35 T scanner of an integrated MRI-Linac: Characterization of radiomics features in phantom and patient data. Technol. Cancer Res. Treat..

[83] Fernandes C.D., Dinh C.V., Walraven I., Heijmink S.W., Smolic M., van Griethuysen J.J., Simões R., Losnegård A., van der Poel H.G., Pos F.J. (2018). Biochemical recurrence prediction after radiotherapy for prostate cancer with T2w magnetic resonance imaging radiomic features. Phys. Imaging Radiat. Oncol..

[84] Li Y., Huang X., Xia Y., Long L. (2020). Value of radiomics in differential diagnosis of chromophobe renal cell carcinoma and renal oncocytoma. Abdom. Radiol..

[85] Lichtensztajn D.Y., Hofer B.M., Leppert J.T., Brooks J.D., Chung B.I., Shah S.A., DeRouen M.C., Cheng I. (2023). Associations of Renal Cell Carcinoma Subtype with Patient Demographics, Comorbidities, and Neighborhood Socioeconomic Status in the California Population. Cancer Epidemiol. Biomarkers Prev..

[86] Cotta B.H., Choueiri T.K., Cieslik M., Ghatalia P., Mehra R., Morgan T.M., Palapattu G.S., Shuch B., Vaishampayan U., Van Allen E. (2023). Current landscape of genomic biomarkers in clear cell renal cell carcinoma. Eur. Urol..

[87] Sankin A., Hakimi A.A., Mikkilineni N., Ostrovnaya I., Silk M.T., Liang Y., Mano R., Chevinsky M., Motzer R.J., Solomon S.B. (2014). The impact of genetic heterogeneity on biomarker development in kidney cancer assessed by multiregional sampling. Cancer Med..

[88] Batai K., Bergersen A., Price E., Hynes K., Ellis N.A., Lee B.R. (2018). Clinical and molecular characteristics and burden of kidney cancer among Hispanics and Native Americans: Steps toward precision medicine. Clin. Genitourin. Cancer.

[89] Zhang N., Chen S., Jiang G., Wu Y., Shao J., Liu W., Wang X., Na R., Xu J. (2020). The study on copy number alteration of clear cell renal cancer in Chinese population. J. Cancer.

[90] Varma S., Simon R. (2006). Bias in error estimation when using cross-validation for model selection. BMC Bioinform..

[91] Vabalas A., Gowen E., Poliakoff E., Casson A.J. (2019). Machine learning algorithm validation with a limited sample size. PLoS ONE.

[92] Cawley G.C., Talbot N.L. (2010). On over-fitting in model selection and subsequent selection bias in performance evaluation. J. Mach. Learn. Res..

[93] Shin T., Duddalwar V.A., Ukimura O., Matsugasumi T., Chen F., Ahmadi N., de Castro Abreu A.L., Mimata H., Gill I.S. (2017). Does computed tomography still have limitations to distinguish benign from malignant renal tumors for radiologists?. Urol. Int..

[94] Israel G.M., Bosniak M.A. (2005). How I do it: Evaluating renal masses. Radiology.

[95] Wasim A., Mumtaz F. (2018). Limitations of CT scanning in Bosniak staging of renal cystic carcinoma. J. Surg. Case Rep..

[96] Coughlin C.R., Scharer G.H., Shaikh T.H. (2012). Clinical impact of copy number variation analysis using high-resolution microarray technologies: Advantages, limitations and concerns. Genome Med..

[97] Bier F.F., Kleinjung F. (2001). Feature-size limitations of microarray technology–a critical review. Fresenius J. Anal. Chem..

[98] (2022). Tayside Biorepository. https://www.tissuebank.dundee.ac.uk.

[99] Yap F.Y., Varghese B.A., Cen S.Y., Hwang D.H., Lei X., Desai B., Lau C., Yang L.L., Fullenkamp A.J., Hajian S. (2021). Shape and texture-based radiomics signature on CT effectively discriminates benign from malignant renal masses. Eur. Radiol..

[100] Yi X., Xiao Q., Zeng F., Yin H., Li Z., Qian C., Wang C., Lei G., Xu Q., Li C. (2021). Computed tomography radiomics for predicting pathological grade of renal cell carcinoma. Front. Oncol..

[101] Yeap P.L., Wong Y.M., Ong A.L.K., Tuan J.K.L., Pang E.P.P., Park S.Y., Lee J.C.L., Tan H.Q. (2023). Predicting dice similarity coefficient of deformably registered contours using Siamese neural network. Phys. Med. Biol..

[102] Python Release Python 3.6.0. https://www.python.org/downloads/release/python-360/.

[103] Illumina (2023). Infinium CytoSNP-850K BeadChip Assay Reference Guide. https://support.illumina.com/ko-kr/downloads/infinium-cytosnp-850k-reference-guide-15046990.html.

[104] Illumina (2024). GenomeStudio Software Downloads. https://support.illumina.com/array/array_software/genomestudio/downloads.html.

[105] Illumina (2024). GenomeStudio 2.0 Plug-ins. https://support.illumina.com/downloads/genomestudio-2-0-plug-ins.html.

[106] Illumina Microarray General Reference Materials.2024. https://knowledge.illumina.com/microarray/general/microarray-general-reference_material-list/000002766.

[107] University of Pennsylvania (2024). PennCNV: Copy Number Variation (CNV) detection from SNP Genotyping Arrays. https://hpc.nih.gov/apps/PennCNV.html.

[108] (2024). International Standards for Cytogenomic Arrays. https://www.ncbi.nlm.nih.gov/projects/gap/cgi-bin/study.cgi?study_id=phs000205.v2.p1.

[109] The Centre for Applied Genomics (2024). Database of Genomic Variants. https://dgv.tcag.ca/dgv/app/home.

[110] University of California, Santa Cruz (2024). UCSC Genome Browser. https://genome.ucsc.edu/.

[111] (2024). CNV Xplorer. https://cnvxplorer.com/.

[112] Broad Institute (2024). CNV ClinViewer. https://cnv-clinviewer.broadinstitute.org/.

[113] IMGSB (2024). BEDsect: A Tool for Feature-based Annotations of Genomic Datasets. https://imgsb.org/bedsect/.

[114] University of California, Santa Cruz The UCSC Table Browser Data Retrieval Tool. https://genome.ucsc.edu/cgi-bin/hgTables.

[115] Gupta N. (2019). DNA extraction and polymerase chain reaction. J. Cytol..

[116] Corporation P. (2022). Maxwell® RSC DNA FFPE Kit Technical Manual. https://www.promega.co.uk/resources/protocols/technical-manuals/101/maxwell-rsc-dna-ffpe-kit-protocol/.

[117] Corporation P. (2022). Maxwell® RSC Genomic DNA Kit Technical Manual. https://www.promega.co.uk/resources/protocols/technical-manuals/500/maxwell-rsc-genomic-dna-kit-protocol/.

[118] Illumina (2023). InfiniumTM CytoSNP-850K v1.4 BeadChip Data Sheet. https://support.illumina.com/content/dam/illumina/gcs/assembled-assets/marketing-literature/infinium-cytosnp850k-data-sheet-m-gl-01507/infinium-cytosnp850k-data-sheet-m-gl-01507.pdf.

[119] Illumina (2023). iScan System Guide. https://support-docs.illumina.com/ARR/iScan/Content/ARR/FrontPages/iscan.htm.

[120] Breiman L. (2001). Random forests. Mach. Learn..

[121] Louppe G. (2014). Understanding random forests: From theory to practice. arXiv preprint.

[122] Biau G., Scornet E. (2016). A random forest guided tour. Test.

[123] Qi Y. (2012). Random forest for bioinformatics. Ensemble Machine Learning: Methods and Applications.

