# Supplementary material for: Radiogenomics Pilot Study: Association Between Radiomics and Single Nucleotide Polymorphism-Based Microarray Copy Number Variation in Diagnosing Renal Oncocytoma and Chromophobe Renal Cell Carcinoma
Source: Int J Mol Sci. 2024 Nov 21;25(23):12512. doi: 10.3390/ijms252312512 (PMC11641663; doi:10.3390/ijms252312512)
Supplement: Supplementary file 1 [file ijms-25-12512-s001.zip › ijms-3310980-supplementary.pdf]

International Journal of
Molecular Sciences

MDPI

Radiogenomics Pilot Study: Association Between Radiomics and SNP-Based Microarray Copy Number Variation in Diagnosing Renal Oncocytoma and Chromophobe Renal Cell Carcinoma

Abeer J. Alhussaini [1,2,3], Abirami Veluchamy [4], Adel Jawli [1,5], Neil Kernohan [6], Benjie Tang [7,*], Colin N. A. Palmer [8], J. Douglas Steele [1,2] and Ghulam Nabi [1,9]

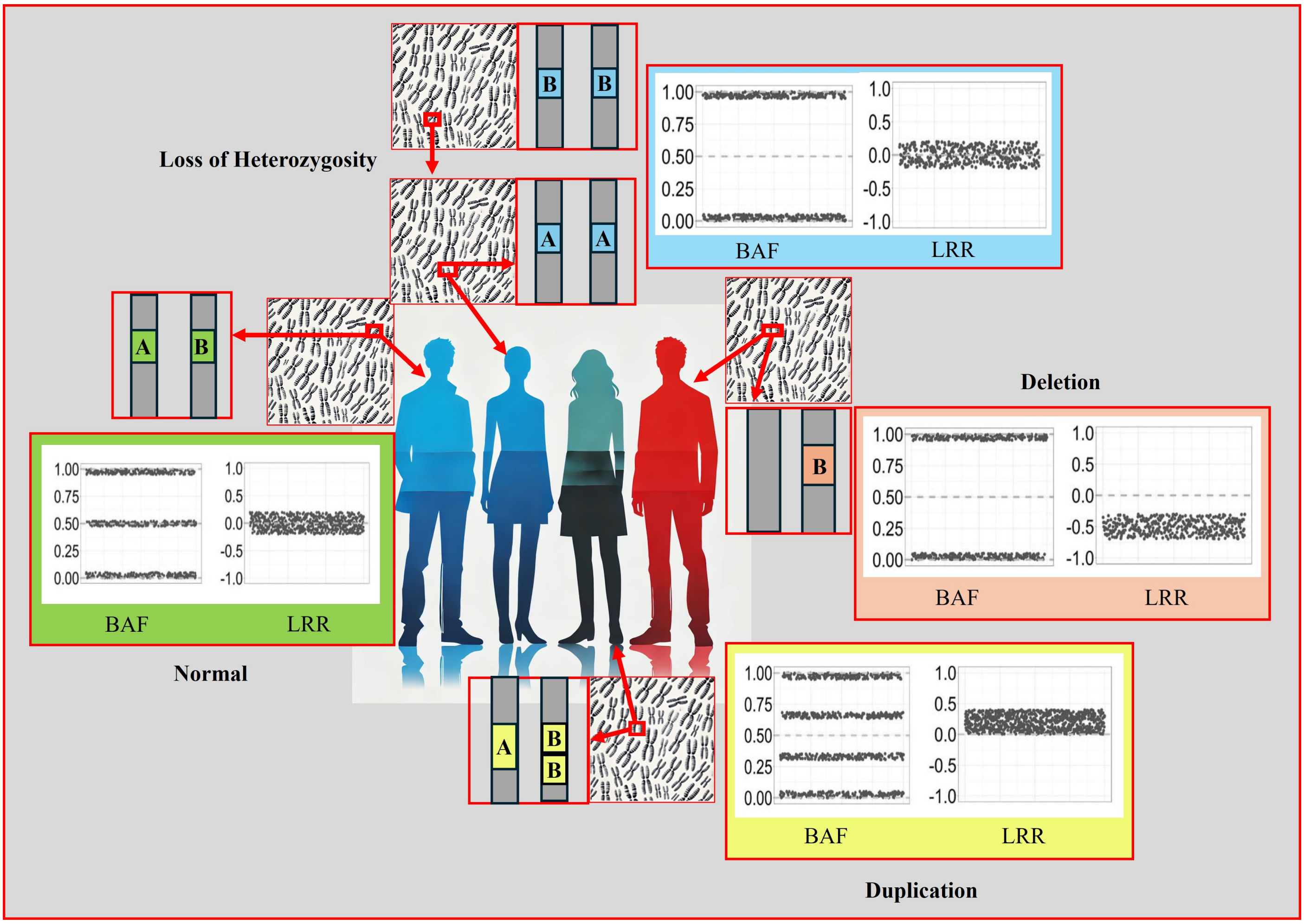

Figure S1. Illustration of different CNV types showing the corresponding changes in B-allele frequency (BAF) and log R ratio (LRR) values. In a normal diploid sample, BAF, which measures the intensity ratio (proportion) of the B allele compared to the total alleles at a specific genetic locus, typically shows values of 0.0, 0.5, and 1.0, representing the AA, AB, and BB genotypes respectively. In the case of a deletion of one allele, the BAF will shift towards 0.0 or 1.0, depending on whether the remaining allele is A or B, reflecting the presence of only one allele at that locus. Duplications lead to BAF values between the typical 0.0, 0.5, and 1.0, depending on the proportion of A and B alleles in the extra copy, often clustering around 0.33 for AAB and 0.66 for ABB. For regions without CNV, the Log R Ratio (LRR), which measures the total signal intensity compared to a reference genome to indicate copy number changes, should hover around 0, reflecting no deviation from the expected copy number. In a deletion, the LRR drops below 0, indicating a reduction in the overall copy number, while in a duplication, the LRR increases above 0, reflecting an extra copy of the affected region.

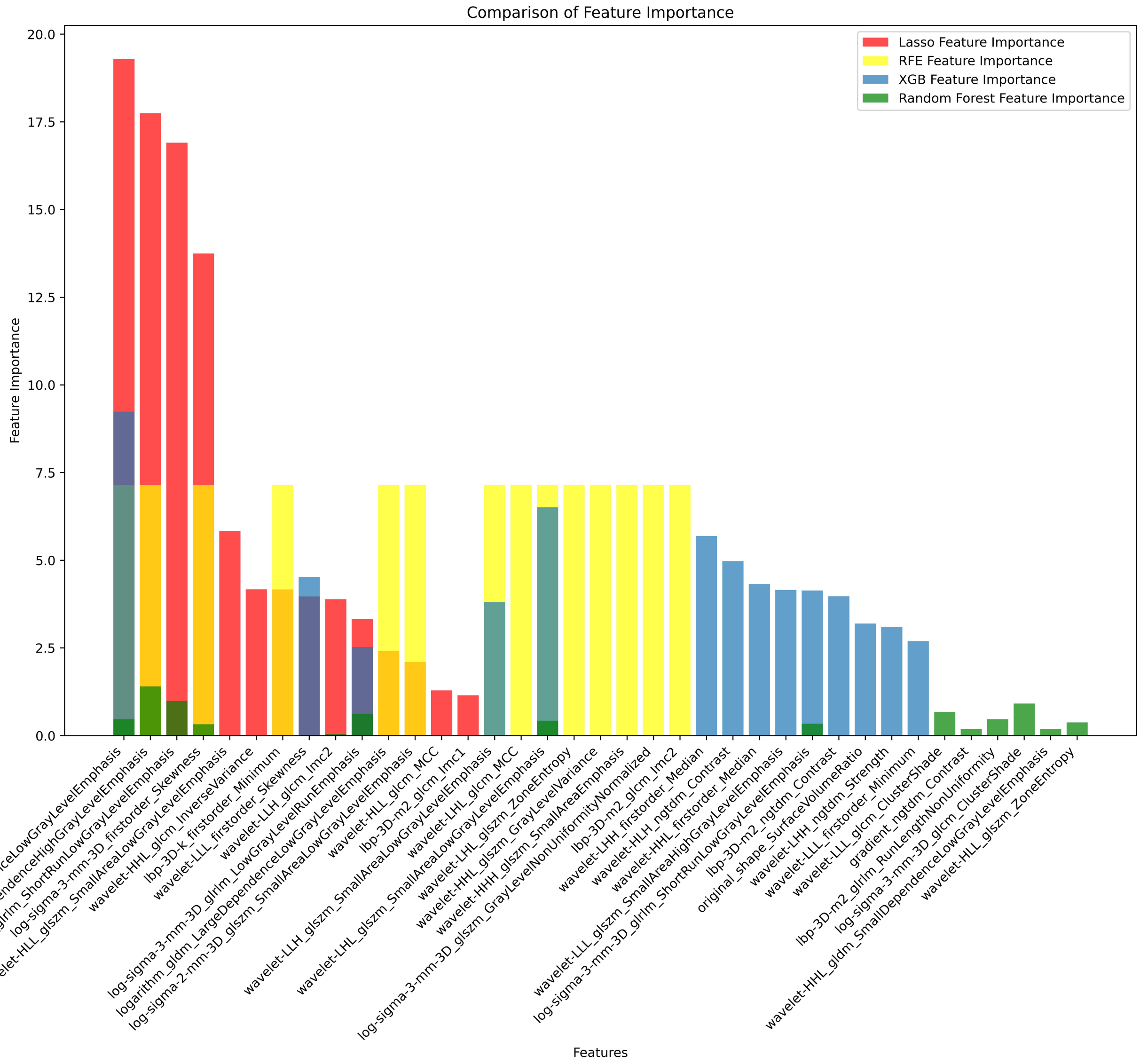

Figure S2. Comparison of feature importance between the four feature selection algorithms.

Table S1. The parameters for each of the fourteen genotypes analysed by cnvPartition are displayed. For homozygous deletions (DD), BAFs are modelled as a uniform distribution between zero and one. All other genotypes are modelled using Gaussian distributions with the specified parameters. The genotype AABB is excluded from modelling because it would represent two independent duplication events, which are rare in nature. (CN = copy number, DD = double deletion, SD = standard deviation) [1].

Genotype	CN	LRR-Mean	LRR-SD	BAF-Mean	BAF-SD
DD	0	-5	2	NA	NA
A	1	-0.45	0.18	0	0.3
B	1	-0.45	0.18	1	0.3
AA	2	0	0.18	0	0.3
AB	2	0	0.18	0.5	0.3
BB	2	0	0.18	1	0.3
AAA	3	0.3	0.18	0	0.3
AAB	3	0.3	0.18	1/3	0.3
ABB	3	0.3	0.18	2/3	0.3
BBB	3	0.3	0.18	1	0.3
AAAA	4	0.75	0.18	0	0.3
AAAB	4	0.75	0.18	0.25	0.3
ABBB	4	0.75	0.18	0.75	0.3
BBBB	4	0.75	0.18	1	0.3

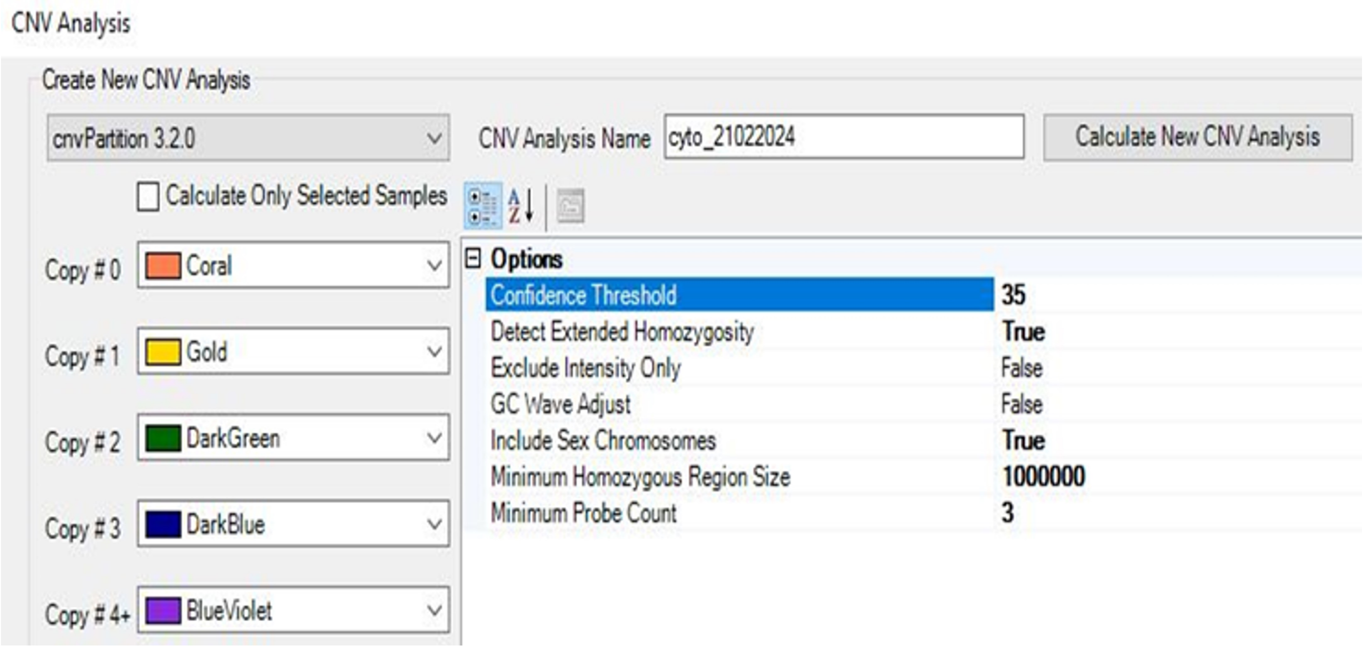

Figure S3. Visualisation of CNV analysis settings using the Illumina Genome Viewer within the Chromosome Browser. The figure represents the various standard parameter thresholds that were used for CNV analysis, including confidence threshold of 35 and minimum probe count of 3. The left panel indicates colour coding of copy numbers (CN) as determined during the analysis.

Table S2. The Illumina Genome Viewer, integrated within Genome Studio, is utilised to graphically visualise copy number regions and to display copy number analysis results in tabular format [2].

CNV-Type	CNV-Value	CNV-Confidence
Normal	2	Blank
Duplication	3 or 4	Contains Value
Deletion	1	Contains Value
Copy Neutral-LOH	2	Contains Value

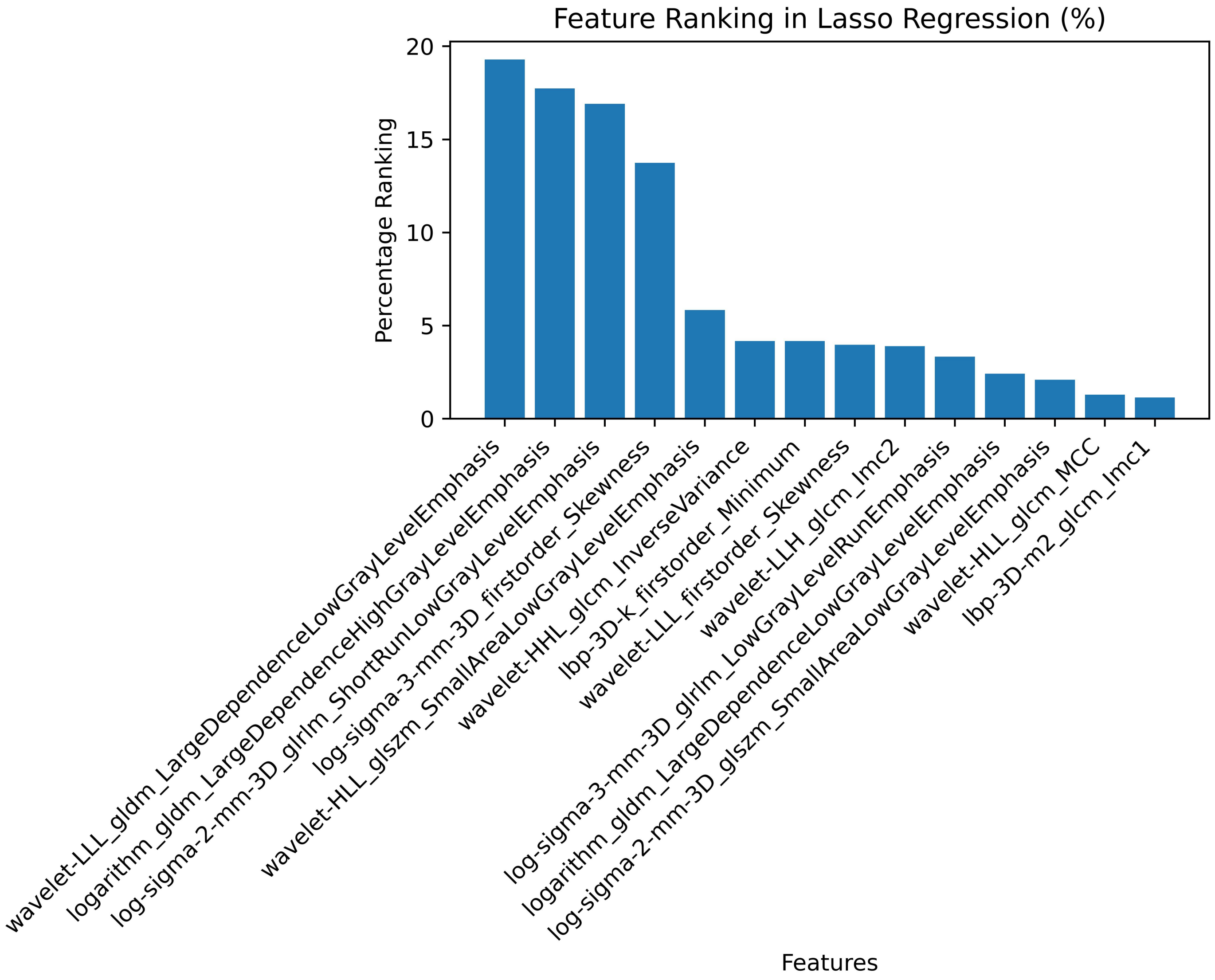

Figure S4. Representation of the feature ranking using Lasso Regression.

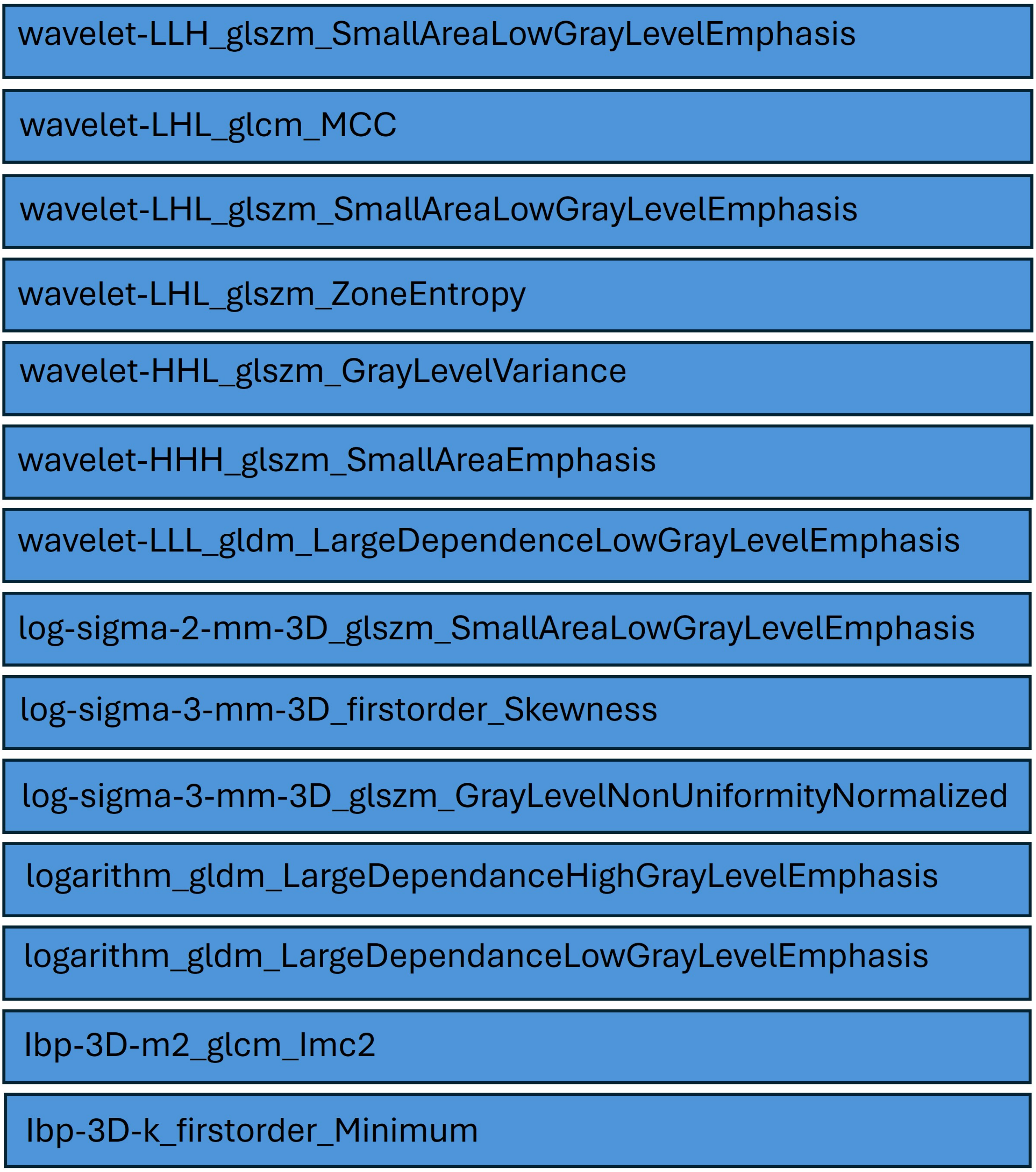

Figure S5. Representation of the best 14 features selected using RFE.

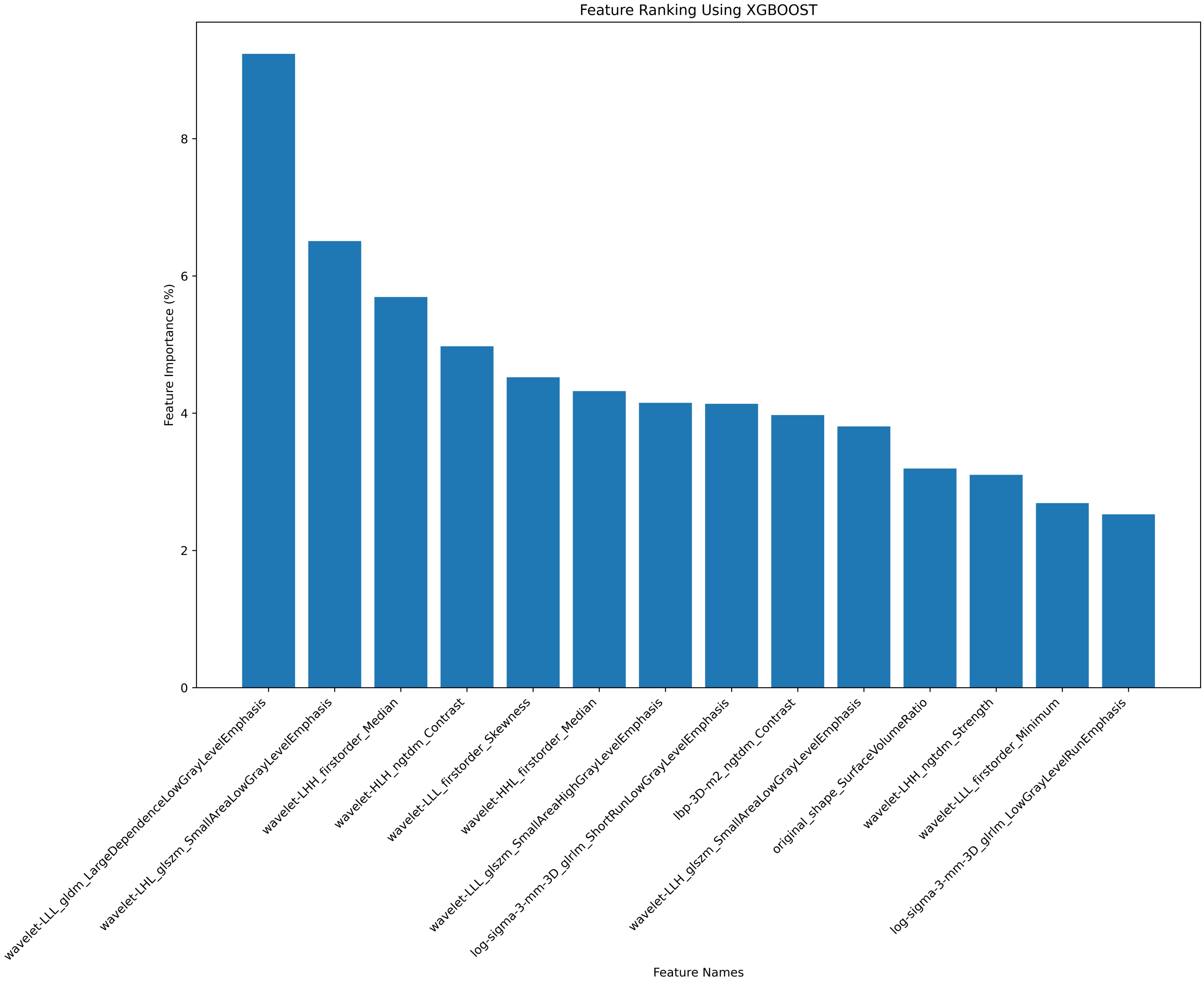

Figure S6. Representation of the feature ranking using XGBOOST.

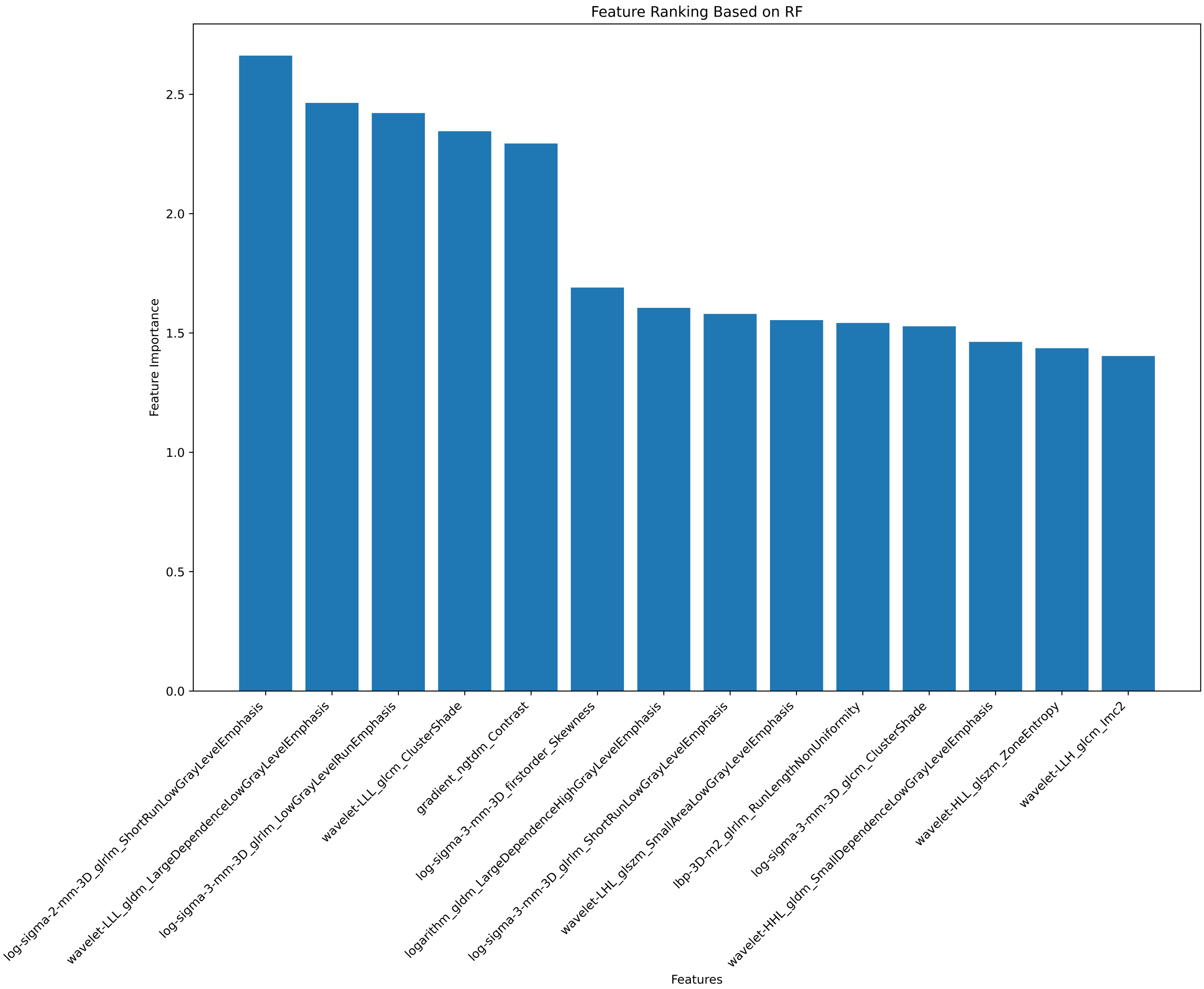

Figure S7. Representation of the feature ranking using RF.

Table S3. Correlation of radiomic features with histopathological differentiation of ChRCC and RO using various filters.

#	Filter Type	Feature Category	Radiomic Feature	Correlation *(r)*	p-value (t)
1	Log Sigma 3 mm 3D	First Order	Skewness	0.39	0.698
2	Wavelet LLL	First Order	Skewness	-0.37	0.527
3	LBP 3D k	First Order	Minimum	0.37	0.79
4	Wavelet LLH	GLCM	Informational Measure of Correlation '2' (IMC2)	0.25	0.988
5	Wavelet LLL	GLDM	Large Dependence Low Gray Level Emphasis (LDLGLE)	-0.38	0.85
6	Logarithm	GLDM	Large Dependence Low Gray Level Emphasis (LDLGLE)	-0.12	0.594
7	Logarithm	GLDM	Large Dependence High Gray Level Emphasis (LDHGLE)	0.34	0.07
8	Log Sigma 3 mm 3D	GLRLM	Low Gray Level Run Emphasis (LGLRE)	0.27	0.69
9	Log Sigma 2 mm 3D	GLRLM	Short Run Low Gray Level Emphasis (SRLGLE)	0.33	0.626
10	Log Sigma 3 mm 3D	GLRLM	Short Run Low Gray Level Emphasis (SRLGLE)	0.29	0.956
11	Log Sigma 2 mm 3D	GLSZM	Small Area Low Gray Level Emphasis (SALGLE)	0.19	0.079
12	Wavelet LHL	GLSZM	Small Area Low Gray Level Emphasis (SALGLE)	0.2	0.137
13	Wavelet LLH	GLSZM	Small Area Low Gray Level Emphasis (SALGLE)	-0.05	0.516

Note: In the table, Pearson's correlation coefficient *(r)* was used to quantify the linear relationship between various radiomic features and the histopathological differentiation of ChRCC and RO. This coefficient is well-suited for measuring the strength and direction of linear associations, where values close to 1 or -1 indicate strong correlations, and values near 0 suggest little to no linear relationship. Its use in this analysis helps identify which features might be most relevant for distinguishing between ChRCC and RO, providing valuable insights for predictive models or diagnostic tools. The T-test *(t)* was employed to determine whether there are statistically significant differences in the means of radiomic features between the two groups, ChRCC and RO. This test assumes normality within each group and is effective for detecting even small differences in means.

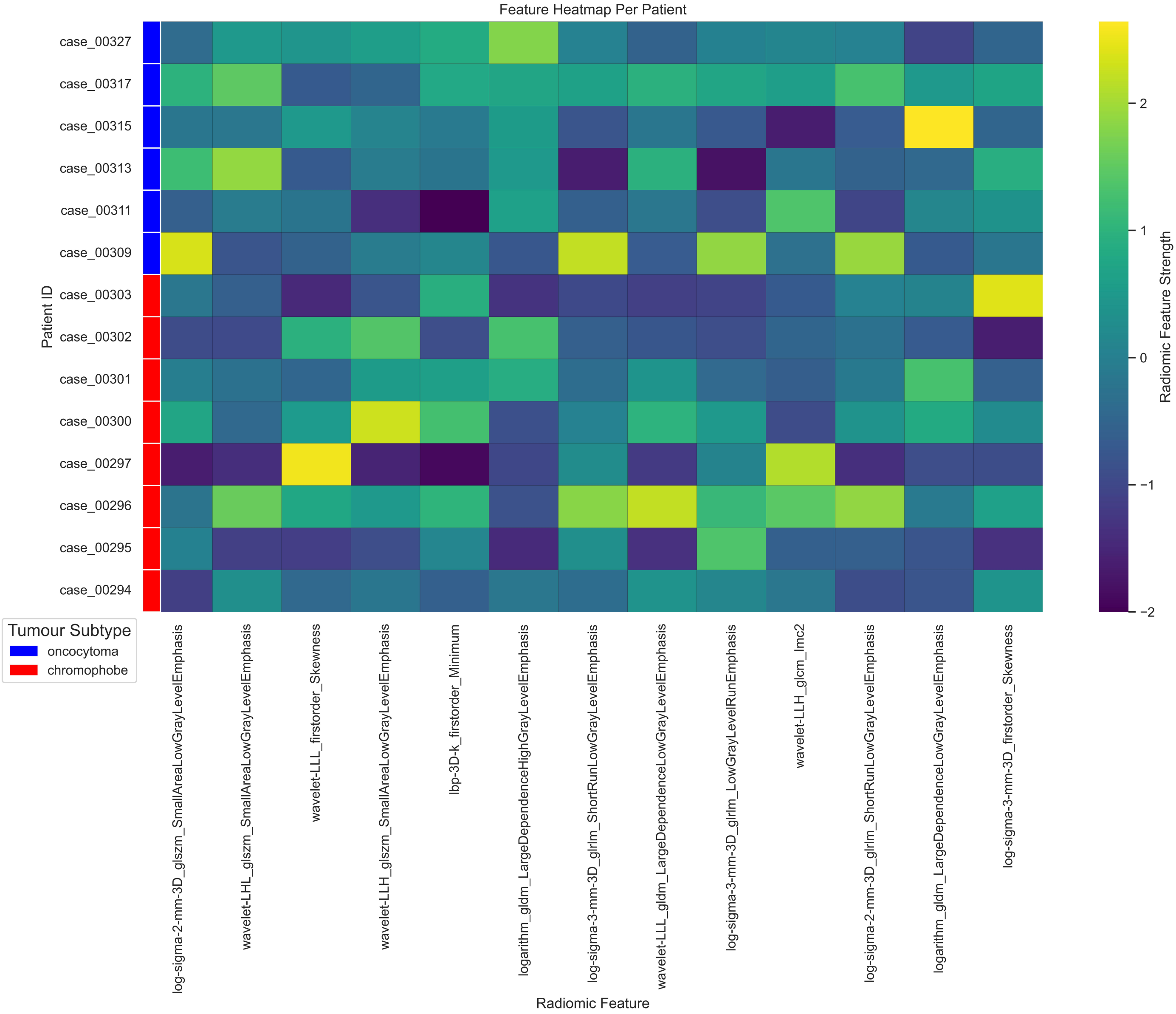

Figure S8. Representation of the heat map showing the correlation between the features and histopathology target of each patient. The colour gradient indicates the intensity of each feature, ranging from blue/purple (low) to yellow (high). The heat map highlights variations in feature expression between the two tumour subtypes, which may assist in differentiating between oncocytoma and chromophobe renal cell carcinoma based on radiomic signatures.

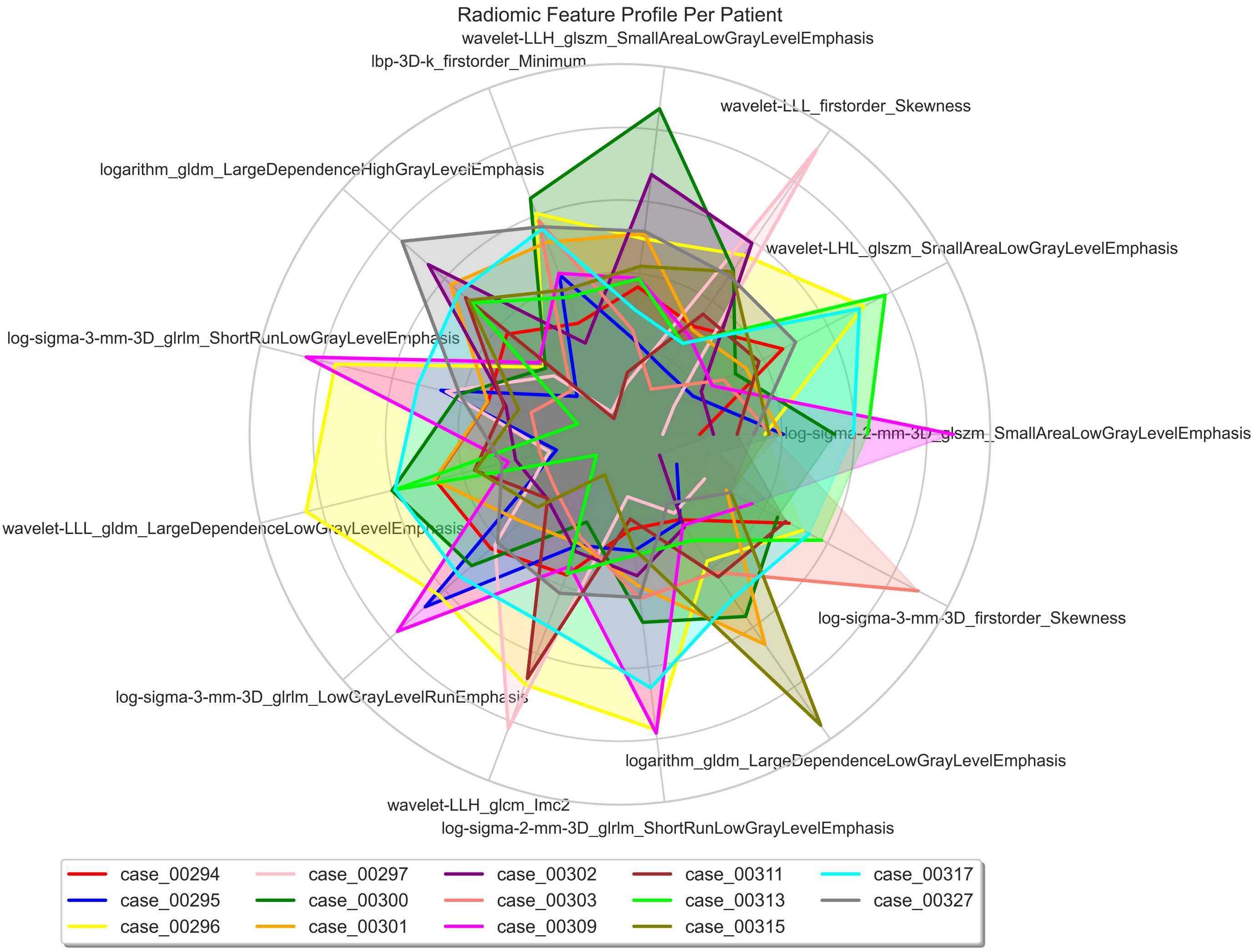

Figure S9. This radar plot illustrates the radiomic feature profiles for 14 patients, highlighting the variation in radiomic feature strength across different cases. Each line represents a unique patient, with radiomic features plotted around the perimeter. The plot enables comparison of individual feature patterns, revealing potential correlations between radiomic characteristics and specific histopathological findings. Different colors represent each patient, allowing for a clear visual comparison of feature expression across the cohort.

Table S4. 24 Genetic and statistical data with correlation values for ChRCC and RO analysis.

Cytoband	Gene	Correlation *(r)*	ChRCC%	RO%	p-value (χ^2)	p-value (t)
1p34.1	RNF115	0.41	12.5	50	0.347	0.14
1q21.3	CTSK	0.41	12.5	50	0.347	0.14
1q21.3	S100A1	0.6	12.5	50	0.347	0.02
1q22	MUC1, RAB25	0.6	0	50	0.109	0.2
1q25.2	ANGPTL1	0.26	25	50	0.687	0.37
1q32.3	MTF2	0.26	25	50	0.687	0.37
1q42.13	TMED5	0.26	25	50	0.687	0.37
1q21.2	MCOLN2, MCOLN3	0.26	25	50	0.687	0.37
1q32.1	LAPTM5	0.42	25	66.67	0.31	0.14
1p36.22	NBL1	0.41	12.5	50	0.347	0.14
2q24	ERBB4	0.45	37.5	0	0.301	0.11
6q13	LMBRD1	0.45	37.5	0	0.301	0.10
6q14.1	TPBG	0.35	25	0	0.58	0.21
6q14.1	MANEA	0.45	37.5	0	0.301	0.10
6q16.3	HACE1	0.45	37.5	0	0.301	0.10
10q11.23	PRKG1	0.45	37.5	0	0.301	0.10
10q22.1	CSTF2T	0.45	37.5	0	0.301	0.10
10p12.33	MRC1	0.45	37.5	0	0.301	0.10
10p12.1	STAM	0.45	37.5	0	0.301	0.10
10q22.2	PPP3CB	0.47	0	33.33	0.58	0.09
17q21.31	SLC4A1	0.32	0	16.67	0.88	0.26
Xp21.2	DMD	0.61	87.5	33.33	0.126	0.02
Xp11.23	DYNLT3	0.61	87.5	33.33	0.126	0.02
Xq28	CTAG1B, MAGEA4, MAGEA3	0.47	75	33.33	0.31	0.09

Note: In this table, the Pearson correlation coefficient *(r)* was employed to measure the strength and direction of the linear relationship between CNV values of specific genetic regions and the histopathological differentiation of ChRCC and RO. The Chi-square test (χ^2) was used to assess whether there is a statistically significant association between categorical variables—specifically, the presence or absence of certain CNVs (categorised as gain, loss, or neutral) and the histopathological classifications of ChRCC and RO—helping to determine if the distribution of these categorical CNV data differs between the two histological types. Additionally, the T-test (t) was applied to compare the means of CNV values between the two groups, ChRCC and RO. This test was appropriate for determining whether there were statistically significant differences in the average CNV values of specific genetic regions between these two histopathological types, under the assumption that the CNV data within each group were approximately normally distributed with similar variances.

Table S5. Representation of the radiomics and cytogenomics features that are highly correlated and mapped with each other and with the histopathology target.

Correlation	Radiogenomics Features n=34
-0.73	ChXp21.2 (DMD) and Log Sigma 3 mm 3D Firstorder Skewness
-0.73	ChXp11.23 (DYNLT3) and Log Sigma 3 mm 3D Firstorder Skewness
-0.65	Ch2q24 (ERBB4) and Log Sigma 3 mm 3D Firstorder Skewness
-0.61	Ch6q14.1 (TPBG) and Logarithm GLDM Large Dependence High Gray Level Emphasis
-0.61	Ch6q14.1 (TPBG) and Wavelet LLL Firstorder Skewness
-0.58	Ch6q13 (LMBRD1) and Wavelet LLL First Order Skewness
-0.58	Ch6q14.1 (MANEA) and Wavelet LLL First Order Skewness
-0.58	Ch6q16.3 (HACE1) and Wavelet LLL First Order Skewness
-0.57	ChXp21.2 (DMD) and Wavelet LHL GLSZM Small Area Low Gray Level Emphasis
-0.57	ChXp11.23 (DYNLT3) and Wavelet LHL GLSZM Small Area Low Gray Level Emphasis
-0.56	Ch6q14.1 (TPBG) and Wavelet LLL GLDM Large Dependence Low Gray Level Emphasis
0.5	Ch1q21.3 (S100A1) and Wavelet LHL GLSZM Small Area Low Gray Level Emphasis
0.5	Ch1q22 (MUC1, RAB25) and Wavelet LHL GLSZM Small Area Low Gray Level Emphasis
-0.5	Ch1q21.3 (S100A1) and Log Sigma 3 mm 3D GLRLM Low Gray Level Run Emphasis
-0.5	Ch1q22 (MUC1, RAB25) and Log Sigma 3 mm 3D GLRLM Low Gray Level Run Emphasis
-0.5	Ch10q11.23 (PRKG1) and Wavelet LLH GLCM Imc2
-0.5	Ch10q22.1 (CSTF2T) and Wavelet LLH GLCM Imc2
-0.5	Ch10p12.33 (MRC1) and Wavelet LLH GLCM Imc2
-0.5	Ch10p12.1 (STAM) and Wavelet LLH GLCM Imc2
0.47	Ch1q32.1 (LAPTM5) and Wavelet LHL GLSZM Small Area Low Gray Level Emphasis
0.46	Ch10q22.2-q23.1 (PPP3CB) and Logarithm GLDM Large Dependence High Gray Level Emphasis
0.45	Ch17q11.1-q21.32 (SLC4A1) and Logarithm GLDM Large Dependence High Gray Level Emphasis
-0.45	Ch6q13 (LMBRD1) and Wavelet LLH GLCM Imc2
-0.45	Ch6q14.1 (MANEA) and Wavelet LLH GLCM Imc2
-0.45	Ch6q16.3 (HACE1) and Wavelet LLH GLCM Imc2
-0.45	Ch17q21.31 (SLC4A1) and Logarithm GLDM Large Dependence Low Gray Level Emphasis
-0.44	Ch2q24 (ERBB4) and Wavelet LHL GLSZM Small Area Low Gray Level Emphasis
0.43	Ch1q25.2 (ANGPTL1) and Wavelet LHL GLSZM Small Area Low Gray Level Emphasis
-0.42	ChXq28 (CTAG1B, MAGEA3, MAGEA4) and Wavelet LHL GLSZM Small Area Low Gray Level Emphasis
-0.41	Ch6q14.1 (TPBG) and Wavelet LLH GLSZM Small Area Low Gray Level Emphasis
-0.41	Ch6q14.1 (TPBG) and Wavelet LHL GLSZM Small Area Low Gray Level Emphasis
0.41	Ch1q21.3 (S100A1) and Logarithm GLDM Large Dependence High Gray Level Emphasis
0.41	Ch1q22 (MUC1, RAB25) and Logarithm GLDM Large Dependence High Gray Level Emphasis
-0.41	Ch6q14.1 (TPBG) and Wavelet LHL GLSZM Small Area Low Gray Level Emphasis

Note: Pearson's correlation coefficient (r) is used to quantify the strength and direction of the linear relationship between radiomic features and cytogenomic variations, as well as their association with histopathological outcomes. By using Pearson's correlation, we were able to identify and measure the degree to which specific genetic alterations (represented by cytogenomic features) are associated with specific radiomic characteristics. This analysis helps to highlight potential radiogenomic biomarkers that could be relevant for differentiating between ChRCC and RO based on their histopathological classification, contributing valuable insights for both diagnosis and potential treatment strategies.

Table S6. Representation of the number and percentage of genome segments with duplication, deletion, and loss of heterozygosity for each patient. The table also shows the mean size of the affected segments in each category.

Subtype	Patient	DUP	DEL	LOH	Total	DUP (%)	DEL (%)	LOH (%)	DUP (Mb)	DEL (Mb)	LOH (Mb)
ChRCC	294	20	7	4	31	6.35	5.26	2.35	1.14	2.61	37.31
	295	66	5	2	73	20.95	3.76	1.8	14.91	0.33	2.57
	296	3	25	6	34	0.95	18.8	3.53	1.35	0.74	25.18
	297	5	6	10	21	1.59	4.51	5.88	1.72	3.14	15.26
	300	9	13	8	30	2.6	9.77	4.71	10.69	0.03	20.93
	301	44	15	2	61	13.97	11.28	1.78	12.67	1.91	1.82
	302	11	19	131	161	3.49	14.29	77.06	14.21	1.08	1.35
	303	59	0	0	59	18.73	0	0	19.26	0	0
RO	309	6	13	3	22	1.9	9.77	1.76	10.62	1.4	49.52
	311	14	2	0	16	4.4	1.5	0	20.09	6.67	0
	313	4	3	1	8	1.27	2.56	0.59	17.77	5.94	1.22
	315	5	14	3	22	1.59	10.53	1.76	0.037	1.37	50.01
	317	11	10	0	21	3.49	7.52	0	1.62	1.95	0
	327	58	1	0	59	18.41	0.75	0	5.5	0.0065	0
p-value	χ^2	0.35	0.43	0*	-	-	-	-	1.0	0.08	0.78

* Statistical significant difference is considered at 0.05 significance level. DUP: Duplication, DEL: Deletion and LOH: Loss of Heterozygosity.

Table S7. Count of chromosomes per patient, comparing ChRCC cases numbered 294 to 303 with RO cases numbered 309 to 327.

#	1	**2**	**3**	4	5	**6**	**7**	8	**9**	10	**11**	**12**	**13**	**14**	15	16	**17**	18	19	20	**21**	22	**X**	Y
294	9	1	1	0	0	1	0	0	0	2	1	0	0	3	0	0	4	0	0	0	0	1	4	4
295	8	11	6	2	1	10	4	1	4	4	1	1	3	1	0	0	6	0	0	0	2	0	5	3
296	3	1	0	1	1	1	3	2	0	2	2	1	1	1	1	2	0	0	0	1	1	0	6	4
297	2	0	3	0	0	1	0	0	1	0	0	0	1	1	0	0	0	1	0	0	0	0	6	5
300	0	1	0	0	0	2	0	2	1	8	0	0	2	0	1	0	1	0	0	2	1	0	6	3
301	6	4	3	0	0	2	3	0	6	7	2	2	2	1	1	0	4	3	0	1	2	0	8	4
302	25	33	28	0	1	21	1	1	3	10	3	0	8	1	1	0	7	1	0	2	2	0	8	5
303	2	2	4	3	3	2	3	6	2	0	3	2	3	1	1	5	4	4	4	2	1	1	1	0
309	6	0	0	1	2	1	1	0	0	0	0	0	0	0	1	0	0	1	1	0	0	0	4	4
311	3	1	0	1	0	2	0	0	0	3	0	0	1	0	0	1	0	0	0	1	0	0	1	2
313	2	0	0	0	1	0	0	0	0	0	0	0	0	0	0	1	0	0	0	1	0	0	0	3
315	5	0	2	0	0	1	0	1	0	1	1	0	0	0	1	0	0	0	1	0	1	1	2	5
317	7	0	0	0	0	1	0	2	0	1	0	0	1	0	0	1	1	2	0	0	0	1	0	4
327	11	3	1	0	4	5	0	2	1	8	2	0	3	2	2	2	5	2	2	3	0	0	1	0
χ^2	0.9	0	0	0.1	0.4	0	0	0.3	0	0.2	0	0	0	0	1	0.7	0	0.7	0.1	0.5	0	0.7	0	0.9

Bold: Statistical significance at the 0.05 level using chi-squared test (χ^2).

Table S8. Chromophobe RCC and RO CNVs classification and type identified.

	**ChRCC**								**RO**						**p-value**
**Patient #**	**294**	**295**	**296**	**297**	**300**	**301**	**302**	**303**	**309**	**311**	**313**	**315**	**317**	**327**	χ^2
	**Pathogenic**														
**DEL**	0	0	1	0	0	1	0	0	0	0	0	0	0	0	
**DUP**	0	7	0	0	0	3	3	8	0	1	1	0	0	4	0.72
**LOH**	3	0	4	4	4	1	11	0	3	0	0	3	0	0	
	**Likely Pathogenic**														
**DEL**	1	0	6	1	0	3	0	0	2	1	1	1	2	0	
**DUP**	1	21	0	1	2	14	0	31	2	5	1	0	0	16	0.16
**LOH**	0	0	0	0	1	0	5	0	0	0	0	0	0	0	
	**Benign**														
**DEL**	0	1	1	2	0	0	2	0	0	0	0	2	2	0	
**DUP**	0	0	0	0	0	0	0	0	0	0	0	1	1	0	-
**LOH**	0	0	0	0	0	0	0	0	0	0	0	0	0	0	
	**Uncertain Significance**														
**DEL**	6	4	17	3	13	0	13	0	11	0	2	11	6	1	
**DUP**	19	38	3	4	7	27	7	20	4	8	2	4	10	34	0*
**LOH**	1	2	2	6	3	0	115	0	0	0	1	0	0	0	

*Statistical significance at the 0.05 level using chi-squared test (χ^2).

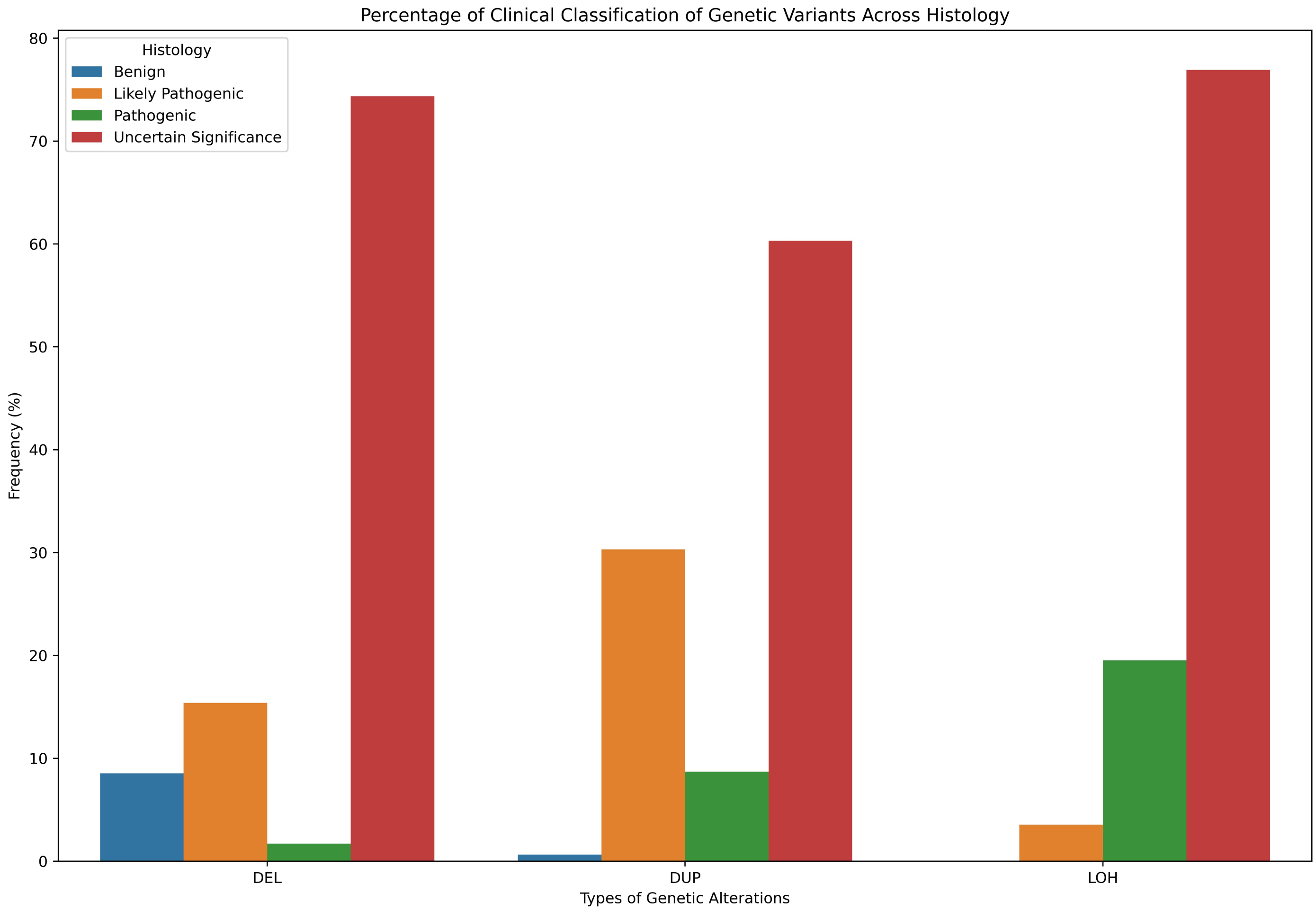

Figure S10. Distribution of clinical classifications of genetic variants across different types of genetic alterations. The bar chart represents the frequency (in percentage) of genetic variants classified as Benign, Likely Pathogenic, Pathogenic, and of Uncertain Significance for three types of genetic alterations: Deletions (DEL), Duplications (DUP), and Loss of Heterozygosity (LOH). The y-axis shows the frequency percentage, while the x-axis categorises the genetic alterations. The chart highlights that the majority of variants within DUP and LOH are classified as having Uncertain Significance, whereas DEL has a more varied distribution across all classifications.

Table S9. The diagnostic performance of the RF model using different cut-offs for Pearson's correlation coefficient (r).

**Correlation (r)**	**ACC**	**SPE**	**SEN**	**AUC**	**MCC**	**F1**
>0.55	81.25	87.50	75.00	85.00	0.63	0.80
>0.50	68.75	87.50	50.00	83.00	0.40	0.62
>0.45	62.50	87.50	37.50	80.00	0.29	0.70
>0.40	75.00	87.50	62.50	78.00	0.52	0.71
>0.30	68.75	87.50	50.00	89.00	0.40	0.62

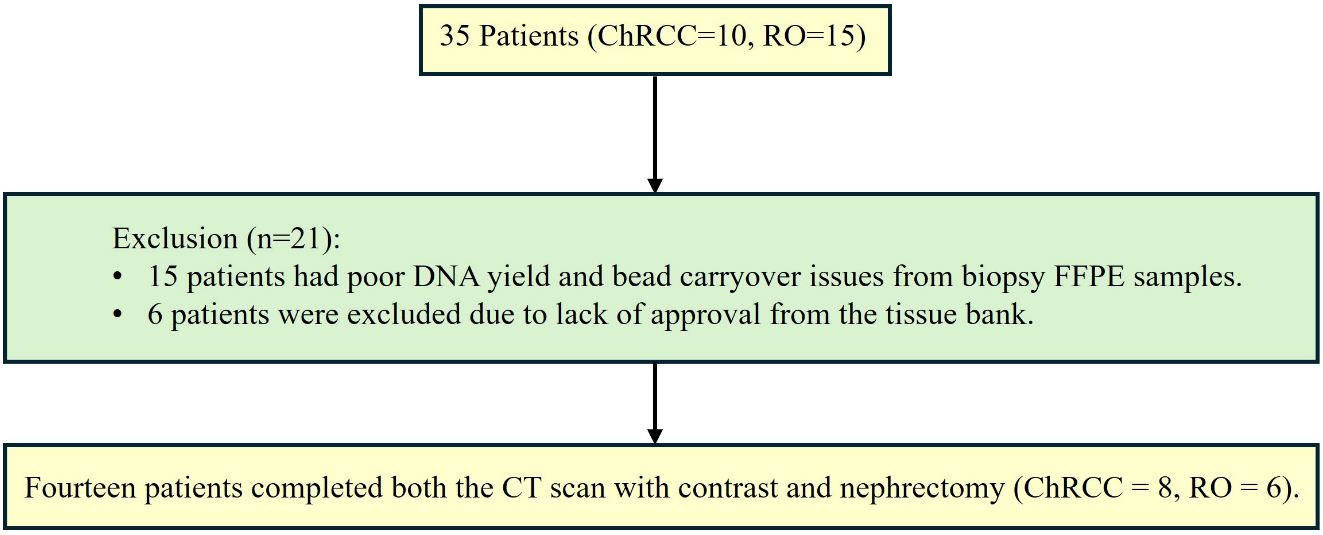

Figure S11. Exclusion and inclusion criteria of the study.

Table S10. Clinical report information of the 35 patients.

Patient	CT Scan and Histopathology Report
**00294***	CT: Renal neoplastic. Histology: RN shows a tumour composed of cells with well-defined cell borders, abundant granular eosinophilic cytoplasm, and central nuclei with surrounding perinuclear halo. Occasional resinoid and binucleate forms are noted. Immunohistochemistry shows diffuse positive staining within the tumour cells for cytokeratin 7 and focal positivity for CD117. Immunohistochemistry for racemase is negative. The morphological and immunophenotypical features are consistent with chromophobe carcinoma.
**00295***	CT: Well-defined enhancing lesion 35 HU non-contrast, 56 HU post-contrast. Histology: PN; chromophobe RCC. Invasion of prerenal fat.
**00296***	CT: Small renal mass lesion. Histology: PN; macroscopically sections show a partial resection of kidney and perirenal fat with a well-circumscribed tumour. The tumour exhibits solid growth and nests of polygonal cells with distinct cell borders. Nuclei are pleomorphic, irregular, and wrinkled. Some cells have eosinophilic granular cytoplasm and other cells have clear cytoplasm. The appearance is suggestive of a chromophobe renal cell carcinoma. While the tumour is present very close to the capsule, it does not extend beyond the capsule. There is no evidence of vascular invasion, within the specimen. The appearance is suggestive of chromophobe RCC.
**00297***	CT: Enhancing mass suggestive of malignancy. Histology: PN; 3.4 cm; chromophobe histology shows a well-circumscribed tumour composed of islands of cells separated by vessels of varying calibre. The tumour cells have well-defined cell borders, a moderate volume of granular eosinophilic cytoplasm, perinuclear halos, and in most cases central slightly irregular nuclei. In areas, nuclear pleomorphism is more prominent, atypical and multinucleate forms are present. The features are those of a chromophobe carcinoma of the kidney.
**00300***	CT: Enhancing heterogeneous mass, partly exophytic unenhanced 36 HU, post-contrast 71 HU. Highly suspicious of malignancy. Histology: PN; sections show a cellular tumour composed of cells with prominent cell borders, and large and occasionally crinkled nuclei with perinuclear halos. The features are typical chromophobe RCC. Margin clear.
**00301***	CT: Renal neoplastic. Histology: PN; microscopically show a lesion composed of nested tumour cells with abundant associated eosinophilic cytoplasm. The nuclei show moderate atypia with often prominent nucleoli and irregular nuclear outlines. Some cells show perinuclear clearing. In areas, the cytoplasm shows peripheral clearing giving a cell wall-type appearance. Much of the tumour is cystically dilated with numerous blood-filled pools and areas of haemosiderin deposition. Morphologically the appearance is most in keeping with those of a renal chromophobe type renal cell carcinoma. Unfortunately, confirmatory immunohistochemistry has not been helpful in this case. Excision margins are clear.
**00302***	CT: Enhancing lesion. Histology: PN; microscopically histology shows a well circumscribed tumour composed of cells with well-defined cell borders, pale eosinophilic granular cytoplasm and central nuclei with prominent nucleoli and occasional raisinoid forms. These cells are arranged predominantly in solid nests with occasional more cystic areas. Immunohistochemistrey has been performed. This shows strong diffuse positive staining for Cytokeratin 7. There is also positivity for CD10. A Hale's colloidal iron stain is positive. Whilst CD10 positivity is less common (around 26% according to some studies), the presence of Cytokeratin 7 positivity and Hale's colloidal iron positivity, together with the morphological features, are consistent with those of a chromophobe carcinoma. The lesion adequately excised.
**00303***	CT: Exophytic left renal cortical lesion concerning for RCC. Histology: PN; microscopically sections show a tumour composed of closely packed cells with clear or mildly eosinophilic cytoplasm, with other cells showing cytoplasmic clearing. Many of the nuclei are irregular and crinkled in shape and some have perinuclear halos. Immunohistochemistry: The tumour cells show positivity for cytokeratin 7 over most of the areas of the tumour in the section. Widespread membrane CD117 staining is also present. Vimentin staining is negative. The appearance is in keeping with a chromophobe carcinoma. The tumour appears well clear of the sinus excision margin. However, in areas at the outer aspect of the tumour where it bulges the capsule; there is evidence of spread beyond the capsule (pT3a).
**00309***	CT: Small renal mass lesion. Histology: PN; a benign oncocytoma characterised by a well-circumscribed tumour composed of nests and tubules of cells with granular eosinophilic cytoplasm and central smooth nuclei. In the centre of the lesion, the tubules and nests are set within a paucicellular edematous stroma, corresponding to the scar seen macroscopically. This benign lesion has clear margins.
**00311***	CT: Enhancing solid left renal mass. Histology: RN; microscopically histology shows a high relatively well-circumscribed tumour composed of variably sized nests of uniform cells with eosinophilic granular cytoplasm and central nuclei with inconspicuous nucleoli. A central oedematous area with infiltration of small nests into this is noted. The morphological features are highly characteristic of a benign oncocytoma. This is confirmed on immunohistochemistry with patchy positivity for Cytokeratin 7 and occasional cells and strong diffuse positivity for CD117 and PAX8. Notably, there is infiltration into the renal sinus fat and an area of vascular invasion is noted. However, this is a recognised phenomenon in benign oncocytomas and in large series following patient outcomes, did not affect their benign behaviour. Left kidney benign oncocytoma with infiltration of the renal sinus and renal vein. Margin clear.

*Represents the patients included for Radiogenomics study.

Table S11. Continuation of clinical report information for the 35 patients.

Patient	CT Scan and Histopathology Report
**00313***	CT: RCC. Highly suspicious of malignancy. Histology: PN; sections show a cellular tumour composed of large nodules of bland cells with abundant eosinophilic cytoplasm and regular nuclei. In areas, smaller islands of similar cells are present with surrounding oedematous stroma. At the periphery of the larger nodules, there are groups of slightly different appearing cells with more hyperchromatic nuclei and less eosinophilic cytoplasm. Central myxoid degeneration/scarring is present. Immunohistochemistry shows that the more eosinophilic tumour cells are negative for vimentin and slightly positive for CD10. There is focal positivity for CK7 but diffuse positivity for CD117. The smaller more hyperchromatic tumour nuclei are positive for vimentin and cytokeratin 7. Despite some unusual morphological and immunohistochemical features, the overall appearance in keeping with the gross appearance of the tumour is best regarded as those of a benign oncocytoma. The tumour appears clear of the sinus excision margin by 2-3 mm.
**00315***	CT: Small renal mass of 28 HU pre-contrast and 73 HU post-contrast. Suspicious of malignancy. Histology: Biopsy; two cores up to 8 mm and fragments. All taken. Microscopically; sections show two cores of renal parenchyma with one of them containing nested groups of closely packed round cells. These cells have abundant intensely eosinophilic cytoplasm, uniform small round central nuclei, mild pleomorphism, evenly distributed chromatin, and smooth nuclear membranes. There is no necrosis or mitotic activity. For immunohistochemistry these cells show strong positive staining with CD117 and patchy positive staining with CK7. The other core shows benign renal parenchyma. The features are in keeping with a benign oncocytoma. PN; macroscopically section displays renal parenchyma predominantly replaced by a neoplasm comprising nests and trabeculae of cells demonstrating intensely eosinophilic and granular cytoplasm, round nuclei, and central nucleoli. The lesion appears vascular and shows no evidence of necrosis. On immunohistochemistry these cells stain diffusely positive with CD117, show scattered positivity with CK7, and stain negative with EMA, Vimentin, and CD10. This shows negative to scattered weak positivity with CK20. Overall, the features are those of a benign oncocytoma which appears 0.4 mm away from the resection margin.
**00317***	CT: Exophytic enhancing solid mass lesion appearance may represent RCC. Histology: PN; oncocytoma. Histology shows a relatively well-circumscribed tumour with a central oedematous area in which small nests and pseudo cystic structures of cells are present which have abundant granular eosinophilic cytoplasm and central nuclei with inconspicuous nucleoli. Immunohistochemistry is positive within the tumour cells for CD117 and PAX8 with only one or two cells staining focally or cytokeratin 7. The features are consistent with a benign oncocytoma. Notably, there is an area of infiltration into the perinephric fat attached to the main specimen; however, this is a recognised phenomenon in benign oncocytoma and does not affect its benign behaviour. The margin is clear of the tumour.
**00327***	CT: RCC. Histology: PN; sections show this neoplasm is disrupted but comprises eosinophilic cells with smooth nuclei with no perinuclear halo's. These neoplastic cells are evenly spaced and immunohistochemistry demonstrates patchy positivity for CK7 and negative staining of CD10. Taking these morphological and IHC phenotypic features this neoplasm is consistent with an oncocytoma. No evidence of a malignant tumour.
**00328**	CT: Enhanced lesion. Histology: PN; sections show an encapsulated but well-delineated tumour composed of oncocytic epithelial cells which have abundant finely granular eosinophilic cytoplasm with large round nuclei, some of which have prominent nucleoli. Occasional perinuclear halo is identified along with binucleate cells; however, these are sparse and not seen across the whole tumour. The oncocytic cells also lack well-defined cell borders. Very focally there is marked nuclear pleomorphism, which is associated with regions that are regarded as being degenerative. There is extensive haemorrhage throughout the tumour and in some areas, small nests and single oncocytic cells are seen set in a background oedematous stroma. There is a marked haemorrhage within the tumour and around the capsule. Immunohistochemistry shows a diffuse expression of CD117 within the tumour with only scattered occasional cells, showing expression of cytokeratin 7. These features are most in keeping with an oncocytoma. There is no evidence of extension into the adjacent perirenal fat and the tumour is clear of the surgical resection margin by 3.8mm. However, in several areas, the capsule has been disrupted and the tumour lies on the surface therefore complete excision cannot be guaranteed.
**00316**	CT: Mass with stellate-like central hypodensity which may represent necrosis or scarring. The appearance is suggestive of but not pathognomonic of an oncocytoma. Histology: PN; microscopically, sections show a cellular tumour composed of cells arranged in solid sheets, nodules, or microcysts. The tumour cells have abundant eosinophilic cytoplasm and the nuclei contain prominent central nucleoli. There is no significant mitotic activity or necrosis. In some areas, the tumour also has an oedematous and focally haemorrhagic stroma. The tumour is well-circumscribed and is well-clear of the inked sinus excision margin. Immunohistochemistry shows that the tumour cells are diffusely positive for pan-cytokeratin and CD117 but only very focally positive for CK7. Staining for CD10, Vimentin, and renal cell carcinoma antigen is negative. The morphological and immunohistochemical features are entirely in keeping with renal oncocytoma.

*Represents the patients included for Radiogenomics study.

Table S12. Continuation of clinical report information for the 35 patients.

Patient	CT Scan and Histopathology Report
**00321**	CT: Renal neoplasm, likely RCC. Suspicious of malignancy. Histology: PN; microscopically, sections show a tumour composed of nodules and strands of tumour cells with abundant eosinophilic cytoplasm and relatively regular nuclei. The nodules are separated by an oedematous and occasionally haemorrhagic stroma with some haemosiderin deposition. The features are those of a benign oncocytoma. The tumour appears confined to the kidney and is well clear of renal sinus excision margin by approximately 5 mm.
**00322**	CT:Small solid lesion in keeping with small renal cell carcinoma. Histology: PN; microscopically, sections show a well circumscribed tumour partly composed of islands of regular cells with abundant eosinophilic cytoplasm in an oedematous stroma. The rest of the tumour appears cystic the cysts being lined by similar tumour cells. The appearance is those of an oncocytoma. The tumour appears clear of the sinus excision by approximately 2 mm.
**00308**	CT: An exophytic hyper-enhancing solid lesion, RCC cannot be excluded, further, follow-up is advised. Histology: RN; microscopically, histology shows a well circumscribed tumour with a central area of hyalinised scarring. The tumour cells are arranged in tubular structures and small nests formed of cells with central nuclei and abundant granular eosinophilic cytoplasm. Immunohistochemistry has been performed. This shows patchy focal positivity for cytokeratin 7, focal positivity for CD10 and positive staining for Vimentin predominantly concentrated in the cells around the central scar area. Immunohistochemistry for CD117 is positive. Hale's colloidal iron shows no evidence of positivity within tumour cells. On close examination, one or two small areas are identified in which there is a slightly greater degree of nuclear abnormality with slightly irregular nuclear contours. Different diagnosis considered are chromophobe carcinoma and oncocytoma, however, on balance the central scar, morphological features of the vast majority of the tumour and the distinctive, pattern of immunohistochemistry with patchy cytokeratin 7 positivity and characteristic Vimentin positivity distribution, the features are regarded on balance as representing those of a benign oncocytoma.
**00324**	CT: Small renal mass demonstrate enhancement and are highly suspicious of small renal malignancies. Histology: PN; the lesion consists of packeted nests of an oncocytic neoplasm. The tumour cells have abundant oncocytic cytoplasm and central very smooth contoured round nuclei with minimal atypia. The features in both cases appear to represent those of benign oncocytoma. There is no evidence of malignancy.
**00298**	CT: Renal lesion Ca/Oncocytoma. Histology: Biopsy; multiple cores up to 12 mm plus fragments. Micro report: histology shows fragmented cores of tissue lesions composed of polygonal cells with abundant eosinophilic slightly granular cytoplasm, with a trabecular architecture. The nuclei are round and central, displaying little variation in size. No prominent cell membranes are identified. Immunohistochemistry shows strong expression of CK7 and EMA by the tumour cells. CD10, vimentin, and CD117 are not expressed. Hales colloidal iron staining shows a very weak focal suggestion of cytoplasmic staining but is considered largely unhelpful in further typing.<br>In conclusion: the appearances are those of an eosinophilic cell renal tumour, the differential diagnosis of which includes the eosinophilic variant of clear cell renal cell carcinoma, chromophobe, and oncocytoma. While the morphological features are not entirely typical, the immunohistochemistry profile is most in keeping with a chromophobe neoplasm. However, a definitive diagnosis cannot be achieved on this small sample, and excision of the lesion or treatment by other means is advised. Discussion of this case at the urology MDT meeting is recommended. The patient under AS and for RFA.
**00299**	CT:Solid renal mass most likely to represent carcinoma. Histology: Biopsy; two cores up to 7 mm. Microscopically the specimen consists of cores of a tumour arranged in a trabecular manner, the cells of which contain abundant eosinophilic cytoplasm. The nuclei are relatively regular with no definite perinuclear halo. The impression on H&E staining was that of an oncocytoma but the immunohistochemical profile contradicts this. The tumour is negative for CD10 and vimentin but shows strong diffuse positivity for cytokeratin 7. This is much in keeping with the eosinophilic variant of chromophobe carcinoma. Patient underwent RFA.
**00304**	CT: Exophytic enhancing small renal mass. Histology: Biopsy; two cores up to 8 mm. All taken. Microscopically, sections show fragments of renal parenchyma and solid tumour composed of clusters and trabeculae of eosinophilic epithelial cells with minimal nuclear pleomorphism and no necrosis. Immunohistochemistry tumour cells show diffuse strong positivity for CD117 and are negative for CK7. Conclusion: taken together the morphological appearance and immunohistochemistry are in keeping with eosinophilic cell neoplasm favouring oncocytoma. Clinical correlation and MDT discussion are advised. Patient under AS.

Table S13. Continuation of clinical report information for the 35 patients.

Patient	CT Scan and Histopathology Report
**00305**	CT: It has a mean HU of 20 on non-cotrast CT, 42 NP and 50 excretory. The heterogeneous left interpolar lesion demonstrates enhancement and although morphologically may represent an oncocytoma, it is suspicious of an RCC. Histology: Biopsy; Three Cores. Microscopically; sections show needle core biopsies of renal parenchyma which are largely replaced by a solid epithelial neoplasm arranged in solid nests and tubules. Tumour cells are remarkably monomorphic displaying abundant eosinophilic slightly granular cytoplasm with round regular nuclei and inconspicuous nucleoli. Nuclear pleomorphism, mitotic activity, and necrosis are not identified. Background perirenal soft tissue is also seen. Immunohistochemistry; tumour cells mildly express a little CD117 but appear almost negative for Cytokeratin 7 except for a few scattered cells. Vimentin and CD10 appear negative. Conclusion: taken together the morphological appearance and immunohistochemical profile favour an oncocytoma. Further clinical correlation and MDT discussion are required. Patient under AS and for RFA.
**00306**	CT: Interpolar renal mass with a central low attenuation, though this could be RCC, oncocytoma cannot be excluded. Histology: Biopsy; two cores up to 25 mm. Microscopically, the cores include some normal kidney but are mainly of a tumour made up of tubular and solid arrangements of round strikingly eosinophilic epithelial cells. There is no significant nuclear pleomorphism. Immunohistochemistry; the cells show patchy expression of CD10 and very focal expression of cytokeratin 7. CD117 is expressed in a membrane fashion. The appearance is entirely in keeping with oncocytoma. There is no histological evidence of malignancy. The patient under AS.
**00310**	CT: Heterogeneous enhancing exophytic mass, most likely to be a renal cell carcinoma. Histology: Biopsy; small fragments, Sections show a tumour consisting of nests of relatively regular cells with small nuclei and abundant eosinophilic cytoplasm. These nests are separated by a vascular oedematous stroma. Immunohistochemistry shows very slight patchy staining for CK7 but negative staining for CD10 and vimentin. The appearance is in keeping with benign oncocytoma.
**00312**	CT:Suspicious enhancing rounded solid (average HU 90). May represent RCC. Histology: Biopsy; microscopically histology shows core biopsies of a tumour with nests and trabecular arrangements of cells that have abundant eosinophilic granular cytoplasm and central round nuclei with occasional nucleoli. Immunohistochemistry has been performed. This is negative for CD10 and shows only focal positivity for cytokeratin 7. The features are of an oncocytic/eosinophilic cell neoplasm, the morphology, and immunophenotype favouring origin from an oncocytoma. Patient under AS.
**00318**	CT: Exophytic enhancing small renal mass. Histology: Biopsy; multiple small cores, the largest 12 mm, microscopically the specimen consists of cores of a tumour composed of regular cells with small round nuclei and abundant eosinophilic granular cytoplasm. No prenuclear haloes or clear cell change is seen. Immunohistochemistry shows that the tumour cells are virtually negative for cytokeratin 7 and negative for vimentin and RCC markers. However, staining for KIT is strongly positive. The morphological and immunohistochemical features are in keeping with benign oncocytoma. Patient under AS.
**00319**	CT: Enhancing exophytic mass. The heterogeneous left interpolar lesion demonstrates enhancement and although morphologically may represent an oncocytoma, it is suspicious of an RCC. Histology: Biopsy; three cores of tissue. Multiple fragments up to 5 mm. Microscopically, most of the specimen consists of connective tissue, but a small focus of neoplastic cells is present. These cells have abundant eosinophilic cytoplasm and small, regular nuclei, with no obvious prominent nucleoli. Immunohistochemical staining shows that the tumour cells show slight positivity for CD10 and very occasional cells are positive for CK7. The tumour cells are negative for vimentin, racemase and HMB45. The features in this biopsy are of a low grade renal epithelial neoplasm. It is often difficult to definitely subtype eosinophilic renal tumours in a small biopsy such as this. However, the morphological and immunohistochemical features are suggestive of an oncocytoma. Patient underwent RFA.
**00320**	CT: Renal mass. Histology: Biopsy; microscopically the sections show strands of tissue that have been derived from a tumour comprising packets, cords, and trabecula of cells set in the relatively abundant loose and pale connective tissue matrix. The tumour cells have bland cytological features but with some variation in nuclear size. Perinuclear halos are not a prominent feature; however, nucleoli can be identified in several of the nuclei. The cytoplasm shows strong granular eosinophilia. Immunohistochemistry: The sections stained for Cytokeratin 7 demonstrate occasional positive cells within the tumour. The majority of the tumour cells are negative. The tumour cells do however show positive staining for CD117 but there is no significant staining for CD10. The principal differential diagnosis, in this case, is between an oncocytoma and a chromophobe form of renal cell carcinoma. The morphology with the immunohistochemical pattern of staining particularly with respect to the very focal staining for cytokeratin 7 would favour an oncocytoma. The patient under AS.

Table S14. Continuation of clinical report information for the 35 patients.

Patient	CT Scan and Histopathology Report
**00307**	CT: Contrast-enhanced renal mass. Histology: Biopsy; microscopically, sections how nests of round cells with abundant finely granular eosinophilic cytoplasm and uniform small, round, and central nuclei with evenly dispersed chromatin. Immunohistochemistry: these cells stained positive for CD10 and CD117 and were focally positive for CK7. They stained negative with vimentin. The morphology appearances and immunohistochemical profile are in keeping with renal oncocytoma. The patient under AS.
**00314**	CT: Solid enhancing exophytic small renal mass. May represent RCC. Histology: Biopsy; three cores up to 30 mm. Micro Report: one of these cores is of the unremarkable renal parenchyma. The other two are samples of neoplasm made up of homogenous rounded epithelial cells with prominent eosinophilic cytoplasm. Nuclear pleomorphism is not notable and there is no obvious mitotic activity. Immunohistochemistry shows only rare cells expressing cytokeratin 7. The tumour cells show membranous expression of CD117 and are negative for vimentin. The appearance is highly suggestive of oncocytoma. Patient under AS.
**00323**	CT: Small renal mass, enhancing solid lesion. Histology: Biopsy; three cores up to 12mm. Micro report: these cores are partially replaced by a tumour with a very eosinophilic morphology. Immunohistochemistry shows diffuse staining for CD117 and only patchy staining for Cytokeratin 7. Taken together the morphology and immunohistochemistry profile would favour an oncocytoma. Patient under AS.
**00325**	CT: Solid renal mass, suspicious of RCC. Histology: Biopsy; two cores. X1 up to 10 mm plus fragments. Sections show a core biopsy of renal parenchyma bearing a focus on neoplastic tissue towards one end. The tumour cells are arranged in rosettes and solid islands. Tumour cells have abundant eosinophilic cytoplasm and small hyperchromatic nuclei. No mitotic activity or necrosis is identified. Results of special stains immunohistochemical, Tumour cells appear entirely negative for cytokeratin 7. Tumour cells moderately express KIT. Tumour cells appear negative for CD10. Tumour cells are negative for RCC. Conclusion: Taken together the morphological appearance and immunohistochemical profile are most in keeping with an oncocytoma. Further clinical correlation and discussion of this case at the urology MDT meeting are strongly recommended. Patient under AS.
**00326**	CT: Enhanced solid small renal mass, in the arterial and delayed phase. Histology: Biopsy; 2018; two cores of tissue up to 14 mm in length. Micro Report: The section shows cores of tissue that have been derived from a tumour that is formed of trabeculae and groups of bland-appearing cells. The cells show oncocytic features with uniform pink cytoplasm. The cells have round nuclei with minimal pleomorphism. Perinuclear halos are not apparent. The cytoplasmic boundaries are not sharply defined. A panel of immunohistochemistry demonstrates that the tumour exhibits diffuse strong positivity for broad-spectrum cytokeratin's recognised by MNF. Scattered positive tumour cells are identified in the sections stained for broad-spectrum cytokeratin's recognised by AE1/3 as well as for cytokeratin 7. The sections stained for cytokeratin 20 show a blush of staining in the tumour cells but the section stained for cytokeratin 14 is negative. The tumour cells are negative for vimentin. There is a patchy variable cytoplasmic expression of CD10. The tumour cells are substantially negative for RCC but do show variable positive staining for EMA. Overall, the appearance indicates cores of tissue derived from a tumour with prominent oncocytic features. The principal differential diagnosis rests between oncocytic features; morphology of the lesion together with the immunohistochemical profile favour oncocytomas rather than a chromophobe RCC. Biopsy; 2019; increased in size; malignant? one core up to 20 mm. Micro Report: morphologically this is a primary renal neoplasm with marked oncocytic features with cells arranged in nests and cords. There is abundant eosinophilic cytoplasm and central round nuclei with smooth contour. Atypical features are not identified and morphological features more suggestive of a chromophobe RCC are not present. Furthermore, immunohestochimistry: there is virtually negative staining for cytokeratin 7 and positive staining for CD117. The features therefore remain consistent with those of an oncocytoma. Further clinical and radiological correlation is advised. The patient under AS.

Table S15. DNA quantification and purity results for FFPE and fresh-frozen samples using NanoDrop Microvolume spectrophotometer. The 260/280 ratio provides a measure of DNA purity, while **ng/μl** indicates the DNA concentration.

**Sample ID**	**260/280**	**ng/μl**
T511 3A (1)	1.75	62.21
T52223 5H	1.84	215.9
T53222 2C	1.78	104.2
T54444 1C	1.83	135.5
T55447 1C	1.81	203.7
T56643 1C	1.82	516.3
T535 2Cre	1.78	104.2
T5721 1Bre	1.83	297.8
T57987 1B	1.83	297.8
T58876 2I	1.77	89.14
T59543 1E	1.78	175.2
T51032 1D	1.83	168.5
T51123 4B	1.85	517.9
T5134 2Cre	1.71	101.1
T512 3A (2)	1.58	37.82
T51308 2C	1.71	101.1
T51444 2E	1.71	66.63
T51555 1A	1.75	145.21
115161 14b	1.74	91.41
114939 3re	1.65	83.63
135177 34e	1.69	69.51
1114188 39	1.65	83.63
115199 07f	1.64	74.89
1154 14Bre	1.74	91.41

Note: This table provides the results of DNA quantification and purity assessment for various FFPE samples and fresh-frozen samples using a NanoDrop Microvolume spectrophotometer. The **260/280 ratio** is used as an indicator of DNA purity, with a ratio of around 1.8 typically considered indicative of pure DNA. Lower ratios may suggest the presence of protein contamination, while higher ratios might indicate RNA contamination. The **ng/μl** column represents the concentration of DNA in each sample, measured in nanograms per microlitre. Higher concentrations indicate larger amounts of DNA in the sample, which are essential for downstream molecular biology applications.

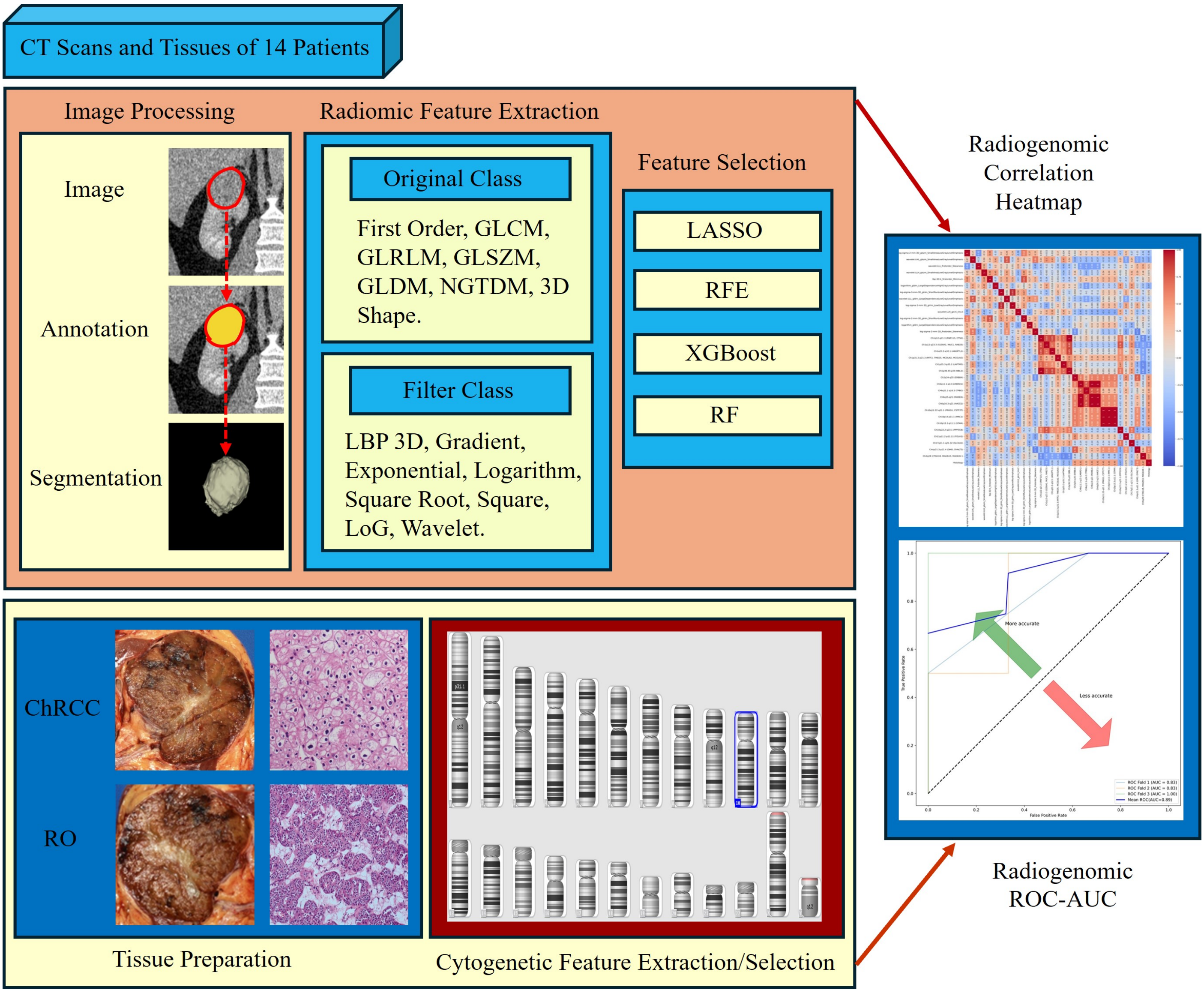

Figure S12. The diagram offers a representation of the study's methodological process.

Table S16. Calculation of the power function to determine the required sample size.

p_1 (ChRCC)	p_2 (RO)	E	Z
0.286	0.714	0.05	1.96
N_i			627.576

Note: N_i is the sample size of each independent sample, p_1 is the proportion of the first sample, p_2 is the proportion of the second independent sample, Z is the Z-score of the confidence interval, and E is the margin of error. From the samples obtained in the first project, a total of 35 samples were analysed. Of these, 10 were identified as chromophobe (ChRCC) and 25 as renal oncocytoma (RO). The proportions for each subtype were calculated, yielding 29% for ChRCC and 71% for RO, which correspond to proportions of 0.29 and 0.71, respectively. The study determined the margin of error to be 0.05 and set the confidence interval at 95%. Using this confidence interval, the corresponding Z-score was calculated. Based on these parameters, the power function was computed, indicating that a sample size of 627.58 would be required for each subtype to achieve the desired statistical power.

Table S17. Representation of Z-scores for two-tailed hypothesis testing at various confidence levels.

**z**	**.00**	**.01**	**.02**	**.03**	**.04**	**.05**	**.06**	**.07**	**.08**	**.09**
**0.0**	.5000	.5040	.5080	.5120	.5160	.5199	.5239	.5279	.5319	.5359
**0.1**	.5398	.5438	.5478	.5517	.5557	.5596	.5636	.5675	.5714	.5753
**0.2**	.5793	.5832	.5871	.5910	.5948	.5987	.6026	.6064	.6103	.6141
**0.3**	.6179	.6217	.6255	.6293	.6331	.6368	.6406	.6443	.6480	.6517
**0.4**	.6554	.6591	.6628	.6664	.6700	.6736	.6772	.6808	.6844	.6879
**0.5**	.6915	.6950	.6985	.7019	.7054	.7088	.7123	.7157	.7190	.7224
**0.6**	.7257	.7291	.7324	.7357	.7389	.7422	.7454	.7486	.7517	.7549
**0.7**	.7580	.7611	.7642	.7673	.7704	.7734	.7764	.7794	.7823	.7852
**0.8**	.7881	.7910	.7939	.7967	.7995	.8023	.8051	.8078	.8106	.8133
**0.9**	.8159	.8186	.8212	.8238	.8264	.8289	.8315	.8340	.8365	.8389
**1.0**	.8413	.8438	.8461	.8485	.8508	.8531	.8554	.8577	.8599	.8621
**1.1**	.8643	.8665	.8686	.8708	.8729	.8749	.8770	.8790	.8810	.8830
**1.2**	.8849	.8869	.8888	.8907	.8925	.8944	.8962	.8980	.8997	.9015
**1.3**	.9032	.9049	.9066	.9082	.9099	.9115	.9131	.9147	.9162	.9177
**1.4**	.9192	.9207	.9222	.9236	.9251	.9265	.9279	.9292	.9306	.9319
**1.5**	.9332	.9345	.9357	.9370	.9382	.9394	.9406	.9418	.9429	.9441
**1.6**	.9452	.9463	.9474	.9484	.9495	.9505	.9515	.9525	.9535	.9545
**1.7**	.9554	.9564	.9573	.9582	.9591	.9600	.9608	.9616	.9625	.9633
**1.8**	.9641	.9649	.9656	.9664	.9671	.9678	.9686	.9693	.9699	.9706
**1.9**	.9713	.9719	.9726	.9732	.9738	.9744	**.9750**	.9756	.9761	.9767
**2.0**	.9772	.9778	.9783	.9788	.9793	.9798	.9803	.9808	.9812	.9817
**2.1**	.9821	.9826	.9830	.9834	.9838	.9842	.9846	.9850	.9854	.9857
**2.2**	.9861	.9864	.9868	.9871	.9875	.9878	.9881	.9884	.9887	.9890
**2.3**	.9893	.9896	.9898	.9901	.9904	.9906	.9909	.9911	.9913	.9916
**2.4**	.9918	.9920	.9922	.9925	.9927	.9929	.9931	.9932	.9934	.9936
**2.5**	.9938	.9940	.9941	.9943	.9945	.9946	.9948	.9949	.9951	.9952
**2.6**	.9953	.9955	.9956	.9957	.9959	.9960	.9961	.9962	.9963	.9964
**2.7**	.9965	.9966	.9967	.9968	.9969	.9970	.9971	.9972	.9973	.9974
**2.8**	.9974	.9975	.9976	.9977	.9977	.9978	.9979	.9979	.9980	.9981
**2.9**	.9981	.9982	.9982	.9983	.9984	.9984	.9985	.9985	.9986	.9986
**3.0**	.9987	.9987	.9987	.9988	.9988	.9989	.9989	.9989	.9990	.9990
**3.1**	.9990	.9991	.9991	.9991	.9991	.9992	.9992	.9992	.9992	.9992
**3.2**	.9993	.9993	.9993	.9993	.9993	.9993	.9994	.9994	.9994	.9994
**3.3**	.9994	.9994	.9994	.9994	.9994	.9994	.9995	.9995	.9995	.9995
**3.4**	.9995	.9995	.9995	.9995	.9995	.9995	.9995	.9995	.9996	.9996

Note: To determine the Z-score corresponding to a 95% confidence interval, we start by recognising that a 95% confidence level leaves 5% of the distribution in the tails (since 100% − 95% = 5%). Because the Z-distribution is symmetrical, this 5% is split evenly between the two tails, leaving 2.5% in each tail. Thus, to find the Z-score, we need to locate the value on the Z-table that corresponds to a cumulative probability of 97.5% (which is 95% + 2.5%). This cumulative probability represents the area under the curve to the left of the Z-score. Looking at the Z-table; We first find 0.9750 in the body of the table, which is the closest value to our desired cumulative probability. This value corresponds to a Z-score of 1.9 in the row and 0.06 in the column, which when added together gives us a Z-score of 1.96. Therefore, the Z-score associated with a 95% confidence interval is 1.96.

1. Technical Lab Work

1.1. Samples

The study included 14 patients with 24 samples; out of these 6 RO patients:

- 4 with only 1 FFPE sample.
- 1 with 2 FFPE samples and 1 frozen tissue samples.
- 1 with both 2 FFPE.

For the 8 ChRCC patients:

- 4 with 1 FFPE sample.
- 1 with 2 FFPE samples.
- 1 with 2 FFPE and 1 frozen tissue.

- 1 with 2 FFPE and 2 frozen tissues.
- 1 with both 1 FFPE and 1 frozen tissue samples.

*1.2. **Samples Preparation***

1.2.1. FFPE Samples

Materials:

- Microtome
- Microtome Blades
- Floating Out Water Bath
- Cold Plate/Ice Tray
- Hot Plate
- Slides
- Forceps
- Paint brush

Methods:

We precisely segment tissue using the Leica RM2235 Rotary Microtome. The microtome's configuration ensures the ideal clearance angle for sectioning, specifically angled to accommodate the microtome RM55 blades. A specimen clamp is utilised to accommodate various cassette clamp sizes. By releasing the specimen clamp lever, the orientation of the specimen clamp can be adjusted using the dials. The thickness of the section can be altered simply by turning the knob, with the selected thickness set at 10 µm. After configuring the microtome, tissue blocks are created. This process involves removing any excess wax from the tissue cassette while ensuring the knife guard and brake are securely in place for safety. Additionally, the knob is adjusted to the desired thickness.

Using the coarse driving wheel, we cautiously advance the tissue block close to the blade after aligning it with precision. To trim the block carefully, ensuring a visible whole tissue face, we typically use a new blade or the unused portion of an old blade. Once trimming is complete, the tissue blocks are placed face down on the ice tray or cold plate, ready for sectioning. To maintain consistent section thickness throughout the sectioning process, we finely adjust the adjustment knob. For sectioning, either a brand-new blade or the unused portion of a blade is utilised. The required tissue block is placed and aligned with the blade for sectioning.

We advance the cassette clamp to create continuous ribbon-shaped pieces by slowly twisting the smooth-turning wheel. A small paintbrush is used to remove the ribbon from the blade, and forceps are employed to lift the sections for further processing. These sections are delicately floated onto a clean water bath after being cut.

We emphasise the importance of avoiding over-expansion by minimising the time sections spend on the water bath to reduce tissue damage. Using a pencil, pertinent information for each section is noted on the frosted portion of the slide. The slide is then placed on a heated plate to accelerate drying and improve section adherence. After each set of sections, we ensure to wipe down the surface of the water bath thoroughly, ensuring no remnants from the previous tissue block are left behind.

After finishing each block, it is essential to clear excess wax from the microtome to maintain equipment cleanliness and avoid any possible carry-over. To promote good section adhesion to the slides and aid in drying, the cut section slides are placed on the hot plate. We ensure to engage the brake and blade guard when our service is completed. Used blades are disposed of in the Sharp's container if necessary. All wax and debris are removed from the microtome, and the water bath is completely emptied and cleaned. Lastly, depending on specific needs, the sliced sections can be stored in slide baskets either

at room temperature or in the oven. Figures S13 and S14 represents labelled FFPE tissue samples on each slide for ChRCC and RO respectively.

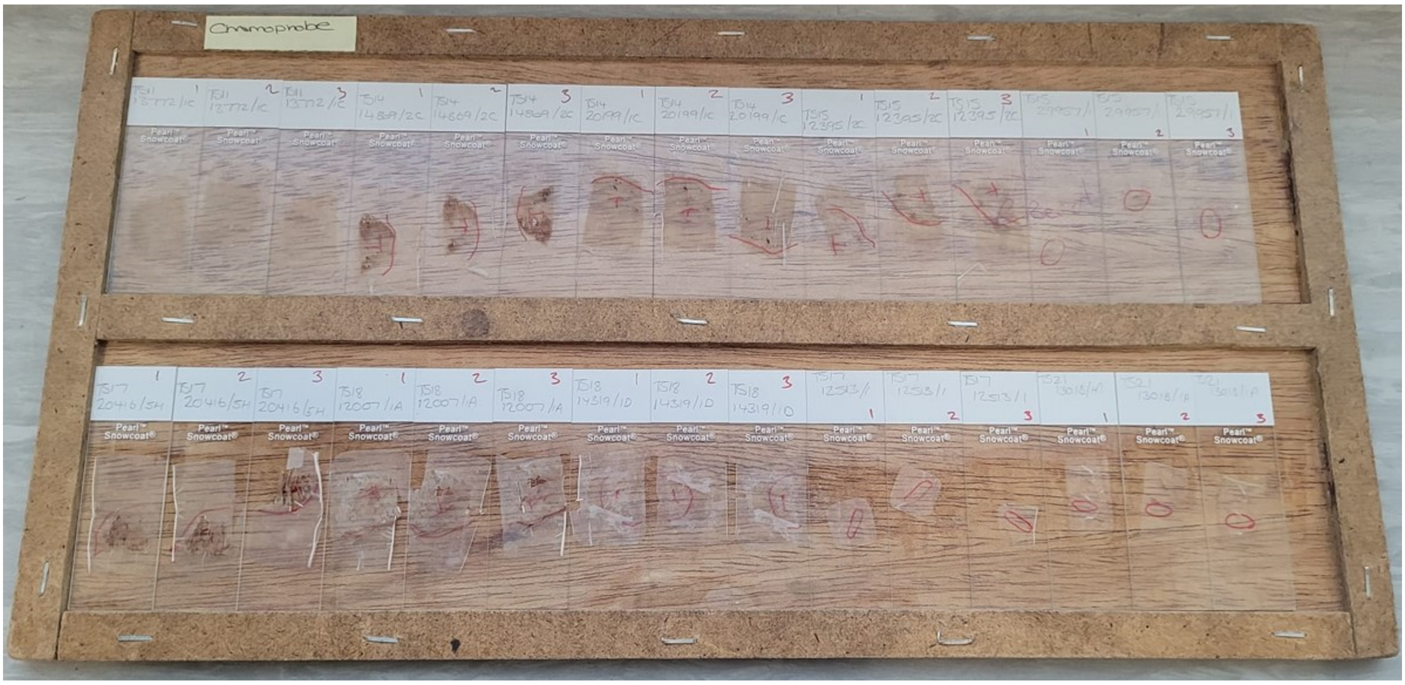

Figure S13. Representation of labelled FFPE tissue samples on each slide for Chromophobe patients.

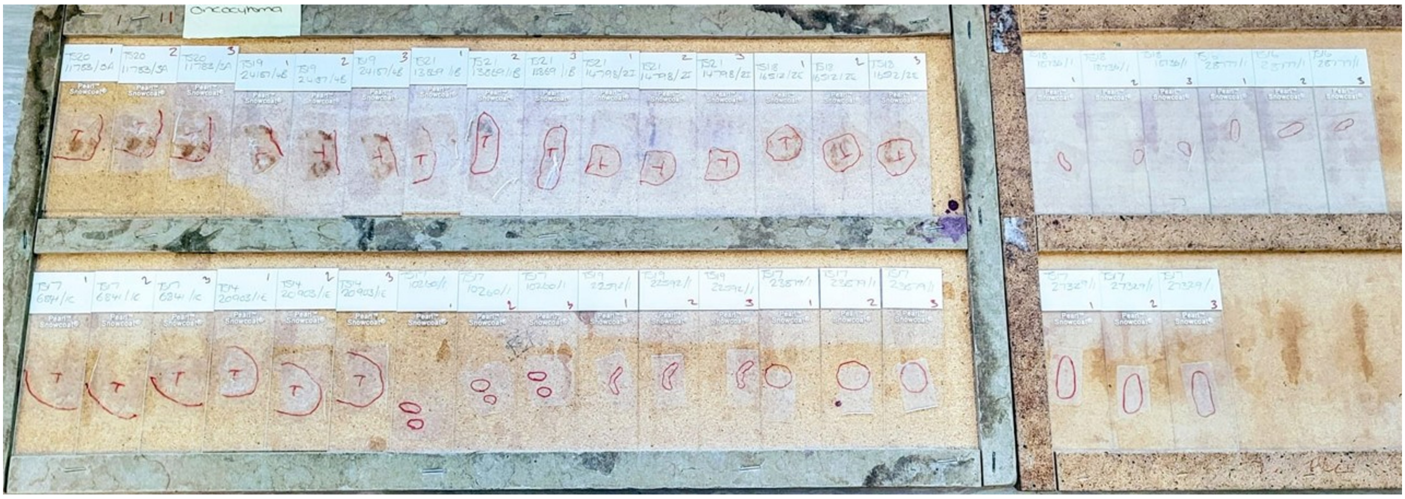

Figure S14. Representation of labelled FFPE tissue samples on each slide for oncocytoma patients.

1.2.2. Fresh-Frozen Samples

Tissue samples are obtained from patients who have given their consent to initiate this process [3]. Once collected, the samples undergo a careful examination to confirm their authenticity. The next step involves transferring these samples to Pathology Reception. Here, the pathologist holds the final decision regarding tissue dissection. To aid in sample orientation, the pathologist may annotate tissue margins following NHS SOP NLHR009, Surgical Cut Up. The pathologist separates both healthy and diseased tissue into labelled cryovials and provides essential information if excess tissue is available and permitted for retention.

The tissue samples in the labelled cryovials are then snap-frozen in liquid nitrogen, with the precise freezing time duly noted on the pathology form. The separated material,

designated as cancerous and normal, should be placed into two distinct cryo-boxes and promptly stored in the freezer at -80 degrees Celsius. Once the boxes are filled, they should be evenly distributed between the two freezers specified to be kept at -80°C.

Ultimately, two copies of the Pathology Form are produced for Tissue Bank documentation. One copy is retained in the "Collected Tissue Pathology and Consent Forms" folder, while the original signed Consent Form and a copy are deposited in the "Awaiting cut-up" folder [3].

1.3. DNA Extraction

An essential method in molecular biology, DNA extraction involves separating and purifying DNA molecules from biological materials such as tissues, blood, or cells [4]. For this study, a protocol from the Maxwell RSC DNA FFPE kit was followed for DNA extraction [5]. Since we had different types of tissue samples, including FFPE and fresh-frozen samples, we adopted two different methods for DNA extraction from each sample type [5,6].

1.3.1. **FFPE Preparation and Preprocessing for DNA Extraction:**

Materials for FFPE samples:

- FFPE tissue samples (of volume 2.0 mm3).
- Maxwell RSC DNA FFPE Kit
- Micro-centrifuge
- Vortex
- Razor blades
- Micro-tubes (1.5-2.0ml)
- Pipettors and pipette tips
- Heating blocks
- Deionised or nuclease free water

Slide-mounted tissue sections were scraped for five minutes. Subsequently, the FFPE tissue sections were inserted into a microcentrifuge tube (patients had 3 slides sectioned for FFPE tissue samples, so we scraped all 3 slides and added them to the tube) and centrifuged for fifteen seconds, collecting the sample at the bottom of the tube. Tissue sections made of FFPE with a maximum volume of 2 mm^3 were utilised [5].

For the manual preprocessing step, sample tubes were filled with 300 μl of mineral oil and vortexed for ten seconds. The samples were then heated to 80°C for 2 minutes and allowed to cool to room temperature. Following the instructions in the Maxwell Manual, a master mix of Lysis Buffer, Proteinase K Solution, and Blue Dye was carefully prepared. Each sample tube was then filled with 250 μl of the master mix, and vortexing was performed for 5 seconds. Layers were separated by centrifugation at 10,000 x g for 20 seconds, with close attention paid to any pellets that might have formed in the aqueous layer. After a 30-minute incubation at 56°C, the sample tubes were transferred to an 80°C heating block for a 4-hour incubation period [5].

Following this phase, the samples were allowed to cool to room temperature and then further incubated for five minutes. Concurrently, cartridge preparation was initiated, following the instructions provided in the protocol. The blue, aqueous phase was finally separated after centrifugation at maximum speed for five minutes. It was promptly transferred to well #1 of a Maxwell8 FFPE Cartridge. The cartridges were placed in the deck trays with well #1 positioned away from the tubes used for elution. The laboratory technique proceeded with accuracy, completing each stage as it progressed and meeting

all standards [5]. Following that, the Maxwell instrument run was performed for FFPE samples.

1.3.2. **Fresh-Frozen Sample Preparation for DNA Extraction**

Materials for Fresh-Frozen Samples:

- Fresh-frozen tissue samples (of volume 2.0 mm3).
- Vortex
- Pipettors and pipette
- Micro-tubes
- Dry heat block
- Deionised or nuclease free water
- 1x Phosphate-buffered saline

We initiated the tissue lysis procedure by labelling the incubation tubes and adjusting the temperature to 56°C. After transferring 5–50 mg of tissue, we centrifuged at top speed for 15 seconds to gather tissue pieces at the bottom of the tube. Subsequently, we carefully changed the pipette tips in each tube to avoid cross-contamination and added 300 μl of Nuclease-Free Water and 30 μl of Proteinase K (PK) Solution. Following each addition, a 10-second vortexing phase was carried out. Then, we vortexed once more after adding 300 μl of Lytic Enhancer (LE2) using the same tip-changing procedure. Without shaking, the incubation was conducted for a minimum of 16 hours at 56°C.

Following the incubation, we vortexed each tube for ten seconds. To remove any remaining undigested material, we next centrifuged at maximum speed for five minutes. Next, we carefully poured all of the supernatant, making sure not to include any pelleted material, into fresh tubes. We dispensed 300 μl of Lysis Buffer into these new tubes, replacing the pipette tips after each addition. We completed another 10-second vortex and readied the cartridges. To ensure homogeneity, the tissue lysate samples were transferred to separate cartridges into the largest well (well #1). During each sample transfer, pipette tips were carefully changed to prevent cross-contamination. The samples were combined with the binding solution by aspirating and dispensing at least ten times [6].

Afterwards we proceeded to the preparation of the Maxwell RSC Genomic DNA Cartridges. We arranged the cartridges on the deck tray outside the instrument in preparation for the purification procedure. Firmly snapping each cartridge into place, we positioned them in the deck trays farthest away from the elution tubes, ensuring they were placed in the largest well, well #1. After confirming that each cartridge was fully inserted into the deck tray on both sides, we carefully removed the seal, ensuring all adhesive residue and sealing tape were removed from the top of the cartridge.

Additionally, one plunger was inserted into well #8 of each Maxwell RSC Cartridge, and 15 μl of RNase A Solution was added to well #3 of the cartridges. An empty elution tube was placed in each cartridge in the deck trays, and to complete the setup, we added 50–200 μl of elution buffer to the bottom of each tube [6].

1.4. Maxwell Instrument Run

We powered on the Maxwell Instrument and Tablet PC, logged in, and double-tapped the desktop icon to open the Maxwell software in order to start the extraction process. Every movable part of the instrument was checked and adjusted by hand. Next, we selected "Start" from the 'Home' menu. The Research Sample Concentrator (RSC) Genomic DNA method was the first one we chose on the 'Methods' screen, and we double-checked that it was the right method before clicking the "Proceed" button. We provided the necessary

expiration and kit lot information when asked. We chose the cartridge locations for the extraction run and validated the Maxwell RSC Genomic DNA technique at the top of the "Cartridge Setup" page. After entering any relevant sample tracking data, we continued.

We placed the deck trays on the Maxwell Instrument platform after ensuring that all the steps on the Extraction Checklist had been completed when the door was opened [6]. These steps included making sure there were samples in cartridge well #1, loaded cartridges, uncapped elution tubes with Elution Buffer, and plungers in well #8. The extraction run started when the "Start" button was pressed, and the door closed as the platform withdrew. The device completed the purification run, displaying the stages that were still in progress and the projected amount of time left on the screen.

When it finished, the method-end message appeared, and we opened the door by following the on-screen directions. We confirmed that the plungers were in cartridge well #8 and consulted the Maxwell Instrument Technical Manual for the Clean-Up procedure in case the plungers had not been removed from the plunger bar. We quickly removed the deck trays after the run to prevent the eluate from evaporating, sealed the DNA-containing elution tubes, and disposed of the cartridges and plungers as hazardous waste. The Maxwell instrument was run twice separately for both the FFPE and fresh-frozen samples [6].

Following that, we use a Qubit Flex fluorometer to measure the DNA. We performed broad range analysis on double-stranded DNA samples by adding stranded 1, stranded 2, and 1 μl of sample DNA before running the sample measurement. To determine the amount of DNA in each sample, we went through this procedure with each one. 10 ng/μl is the minimal acceptable result for DNA quantity. Figures S15, S16 and S17 represent of the lab work for DNA extraction.

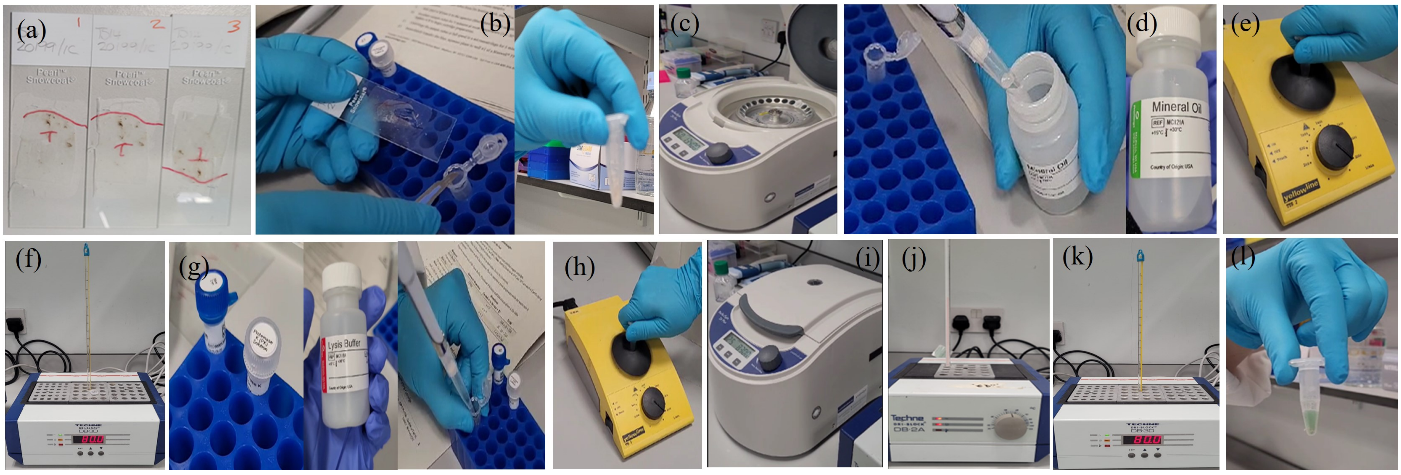

Figure S15. Representation of the lab work for DNA extraction. (a) Use 3 FFPE slides for each patient. (b, c) Scrape the FFPE sample tissue slides, put them into a microcentrifuge tube, and centrifuge for 15 seconds. (d, e) Add 300 μl of mineral oil and vortex for 10 seconds. (f) Heat the sample to 80°C for 2 minutes. (g) Make a master mix of 224 μl of Lysis Buffer, 25 μl of Proteinase K, and 1 μl of Blue Dye; add it to the sample. (h) Vortex for 5 seconds. (i) Centrifuge for 20 seconds. (j) Incubate in a heat block at 56°C for 30 minutes. (k) Heat the sample at 80°C for 4 hours. (l) Remove the samples and leave them at room temperature for 5 minutes.

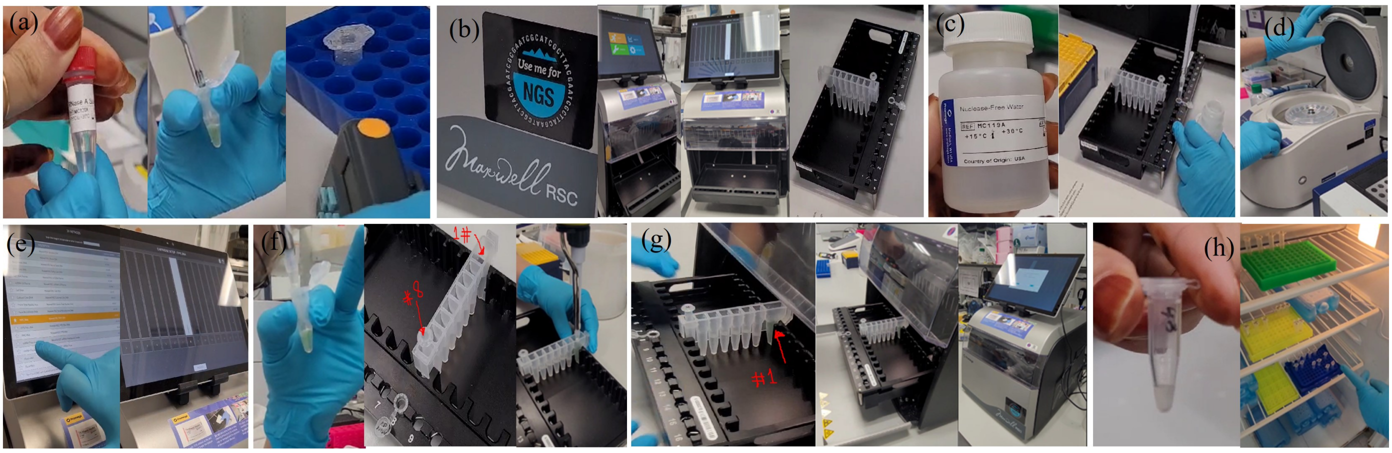

Figure S16. Representation of the lab work for DNA extraction using the Maxwell instrument. (a) Add 10 μl of RNase A solution to the sample and incubate for 5 minutes. (b) Manual preparation of the Maxwell FFPE cartridge: Open the Maxwell instrument. Place the cartridges to be used in the deck tray. For each cartridge, place a plunger into well # 8. For each cartridge in the deck tray, place an empty elution tube. (c) Add 50 μl of Nuclease-Free Water to the elution tube. (d) Centrifuge the incubated DNA for 5 minutes. (e) Select the type of tissue used for Maxwell to extract DNA (FFPE in this case). (f) Transfer the blue part of the sample containing DNA into well # 1. (g) It takes 40 minutes for the Maxwell to extract the DNA. (h) Take the samples and store them in the fridge. The water and the DNA are visible in the tube.

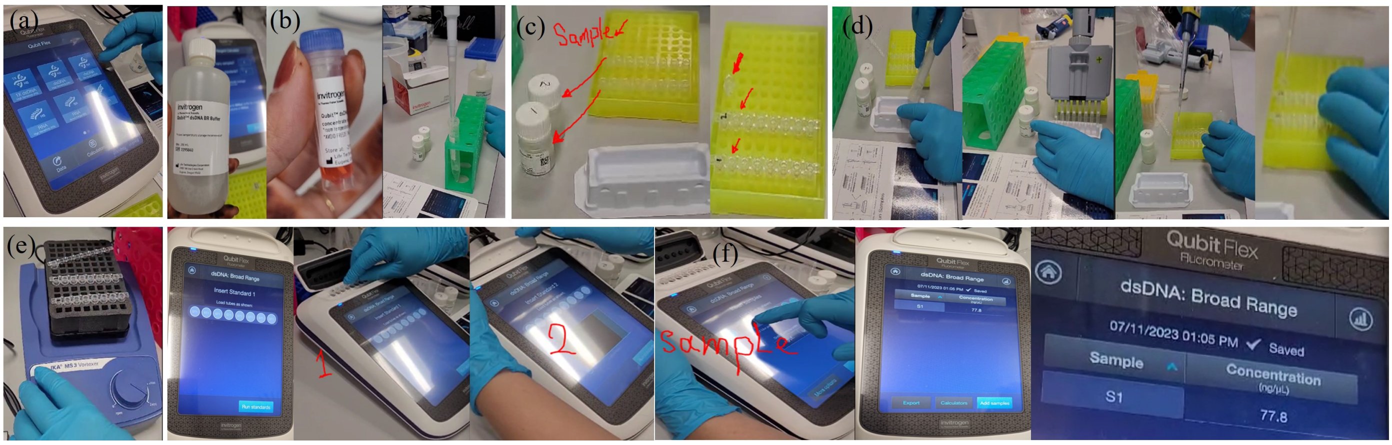

Figure S17. Representation of the lab work for DNA extraction measurement using the Qubit Flex fluorometer and preparing a working solution. (a) Select double stranded DNA and a broad range option. (b) Add 20 μl of dye and 3980 μl of buffer to a tube. (c) Two standards, type 8 and type 1, and one sample are needed. (d) Add the prepared solution to the white plate. For the standards preparation, put 120 μl of the prepared solution in each tube, then add 10 μl of each standard (standard 1 and standard 2) to the tubes. The last empty tube is for the sample; add 199 μl of the prepared solution and 1 μl of the sample to that last tube. (e) Vortex for 3 seconds, then let it sit for two minutes. (f) To measure the DNA, add Standard 1 first, then Standard 2, and finally the sample of 1 μl. The measurement for this sample gave 77.8 ng/μl. The minimum accepted result is 10 ng/μl.

1.5. *BeadChip DNA Assay*

BeadChips rapidly and accurately examines up to one million single nucleotide polymorphism (SNP) sites on a single BeadChip using the potent Infinium Assay [1]. ~850K SNPs with enriched coverage for 3262 dosage-sensitive genes are included in the Infinium CytoSNP-850K v1.4 BeadChip method. 50mer (nucleotides long) SNP probes are used for high target specificity, which improves low-level mosaic identification and provides precise breakpoint estimation for copy number variations and absence of heterozygosity (AOH) events [7].

With as few as 10 consecutive probes, the BeadChip can reliably identify CNV and AOH calls because to its high 15× bead redundancy for a high signal-to-noise ratio. With 848,902 total markers and a call rate of > 98%, the BeadChip takes 200 ng of DNA input refer to Table S18.

The Infinium cytosnp-850k v1.4 beadchip datasheet also provides marker information, which includes the total number of markers (848,902), RefSeq genes (467,422), Absorption, Distribution, Metabolism, and Excretion (ADME) genes (15,153), and more refer to Table S19 [7]. This process further involves following steps as mentioned in Infinium CytoSNP-850K BeadChip Reference Guide [8].

Table S18. Infinium CytoSNP-850K BeadChip product information.

**Feature**	**Description**
Species	Human
No. of samples per BeadChip	8
DNA input requirement	200 ng
Assay chemistry	Infinium HD Super
SNP replicates	15×
No. of SNPs to call CNV	10
**Instrument support**	**iScan System**
Total no. of markers	848,902
Sample throughput per week	960
Scan time per sample	5 min
**Data performance**	**iScan System**
Call rate	99.89%
Reproducibility	99.99%
Log R deviation	0.0929

Table S19. Infinium CytoSNP-850K v1.4 BeadChip marker information.

**Marker categories**	**No. of markers (iScan System)**
Total no. of markers	848,902
RefSeq genes	467,422
RefSeq +/- 10 kb	541,515
ADME genes	15,153
ADME +/- 10 kb	18,590
COSMIC genes	418,131
HLA markers	5145
HLA genes	276
GO genes	137,873
Exonic regions	68,801
Promoter regions	26,814 cont..
X chromosome markers	29,894
Y chormosome marker	1197
PAR/homologous markers	728

1.6. *DNA Amplification*

The genomic DNA (gDNA) samples are added to the MSA1 plate in this stage. To provide enough input for the assay, the samples are first denatured and neutralised in the plate, and then they are amplified during the course of an overnight incubation [8].

Materials:

- 0.1 N NaOH
- DNA samples (50 ng/μl)
- MA1
- MA2
- MSM
- 96-well 0.8 ml midi plate

Methods:

For the procedure of post-amplification, first, we made sure all of the consumables were ready according to Table S20, and heated the Illumina Hybridisation Oven to 37°C. Next, using the given figure as a guide, we prepared the MSA1 plate by adding 20 ng/μl of MA1 to particular wells and designating column 1 for samples for a single BeadChip. Next, we moved 4 μl of the DNA sample (50 ng/μl) from the tubes or DNA plate to the appropriate locations in the MSA1 plate. We filled each DNA sample well with 4 μl of 0.1 N NaOH to aid in hybridisation. A 96-well cap mat was used to seal the MSA1 plate, and it was vortexed for one minute at 1600 rpm.

After that, it was centrifuged at 280 x g. We incubated the sealing mat at room temperature for ten minutes, and then we carefully removed it and placed it aside upside down. Next, we added 75 μl of MSM and 68 μl of MA2 to each sample well. We performed pulse centrifugation and vortexing after resealing the MSA1 plate in the original position. As the last step in our lab work, the sealed plate was placed in the Illumina Hybridisation Oven that had been prepared for 20 to 24 hours. This marked the preparation of the material for additional analysis. Refer to Figure S18 for the procedure of DNA amplification.

Table S20. Preparation of consumables for DNA amplification.

Item	Storage	Instructions
DNA	-25 C to -15 C	Thaw at room temperature
MA1	-25 C to -15 C	Thaw at room temperature, invert 10 times to mix, and then pulse centrifuge
MA2	-25 C to -15 C	Thaw at room temperature, invert 10 times to mix, and then pulse centrifuge
MSM	-25 C to -15 C	Thaw at room temperature, invert 10 times to mix, and then pulse centrifuge

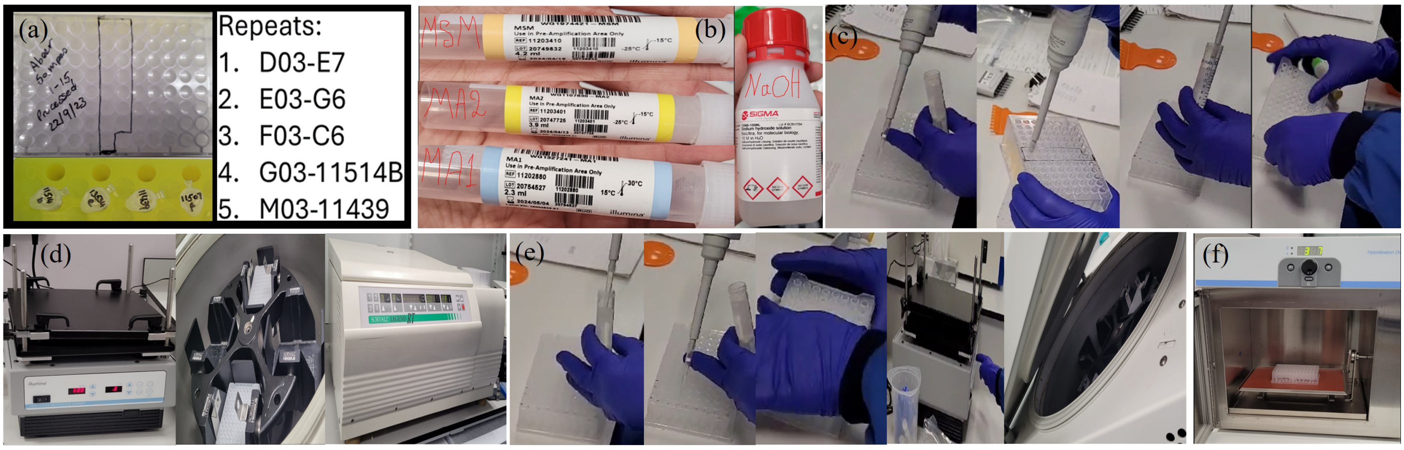

Figure S18. Representation of the lab work for DNA amplification: (a) 19 samples (FFPE and fresh tissue) with 5 repeated samples used in the study. (b) MSM, MA2, MA1, and NaOH are materials used for DNA amplification. (c) Add 20 µl of MA1 to the midi plate wells to prepare MSA1. Add 4 μl of DNA samples to the corresponding wells of the MSA1 plate. Add 4 μl of NaOH to the wells of the MSA1 plate and seal the plate. (d) Vortex for 1 minute, then centrifuge and incubate at room temperature for 10 minutes. (e) Remove the sealing, then add 68 μl of MA2 to each sample well. Add 75 μl of MSM to each sample well, then reseal, vortex, and centrifuge. (f) Incubate in the oven for 24 hours.

1.7. *DNA Fragmentation*

In order to prevent over-fragmentation, endpoint fragmentation is used in this enzymatic DNA fragmentation stage [8].

Materials:

- FMS (1 tube/ can hold 96 samples(we have 19 samples))

Methods:

We ensured that the consumables were ready as indicated in Table S21 and preheated the heat block with the midi plate insert to 37°C.

Table S21. Preparation of consumables for DNA amplification.

Item	Storage	Instructions
FMS	-25 C to -15 C	Thaw at room temperature, invert 10 times to mix, and then pulse centrifuge.

Following the hybridisation process, we took the MSA1 plate out of the oven and pulse centrifuged it at 280 x g. After carefully removing the sealing mat, it was put in a safe place upside down. After that, we filled each sample well in the MSA1 plate with 50 μl of FMS and replaced the cap mat in its original position. The plate was pulse centrifuged at 280 x g after being vortexed for one minute at 1600 rpm. As part of our lab protocol, we then incubated the sealed plate on the heated heat block for an hour. Refer to Figure S19 for DNA fragmentation and precipitation.

1.8. *DNA Precipitation*

In this stage, the DNA is precipitated using PM1 and 100% 2-propanol [8].

Materials:

- 100% 2-propanol
- PM1

Methods:

Until all of the preparations were finished, we left the MSA1 plate on the heat block. After thawing the frozen plates at room temperature, we pulse centrifuged them at 280 x g. In accordance with Table S22, we made sure that all of the necessary consumables were available and heated the heat block to 37°C.

Table S22. Preparation of consumables for DNA amplification.

Item	Storage	Instructions
PM1	-25 C to -15 C	Thaw at room temperature, invert 10 times to mix.

After that, we vortexed each sample well for one minute at 1600 rpm after adding 100 μl of PM1 and resealing the plate with the cap mat in its original position. After five minutes of incubation on the heated heat block, the sealed plate was centrifuged at 280 times the grain size. We set the centrifuge at 4°C in order to get ready for the next centrifuge step. Next, we filled each sample well with 300 μl of 100% 2-propanol and covered it with a fresh, dry cap mat. We were careful not to move the plate until the cap mat was firmly in position. After flipping the plate ten times to make sure everything was well mixed, we let it sit for fifteen minutes at 4°C in the refrigerator.

The plate was then centrifuged for 20 minutes at 3000 x g. We removed the plate and threw away the cap mat after positioning the plate next to another plate of similar weight in the 4°C centrifuge. We promptly inverted the plate to extract the supernatant and held it over an absorbent pad to treat the samples further. We didn't want any liquid on the pad, so we let it drip onto the absorbent pad and then carefully banged the plate down. We vigorously tapped the plate while it was inverted until all of the wells were empty, which took about a minute. Ultimately, we let the pellet air dry for an hour at room temperature by setting the inverted, uncovered plate on a tube rack. Refer to Figure S19 for DNA fragmentation and precipitation.

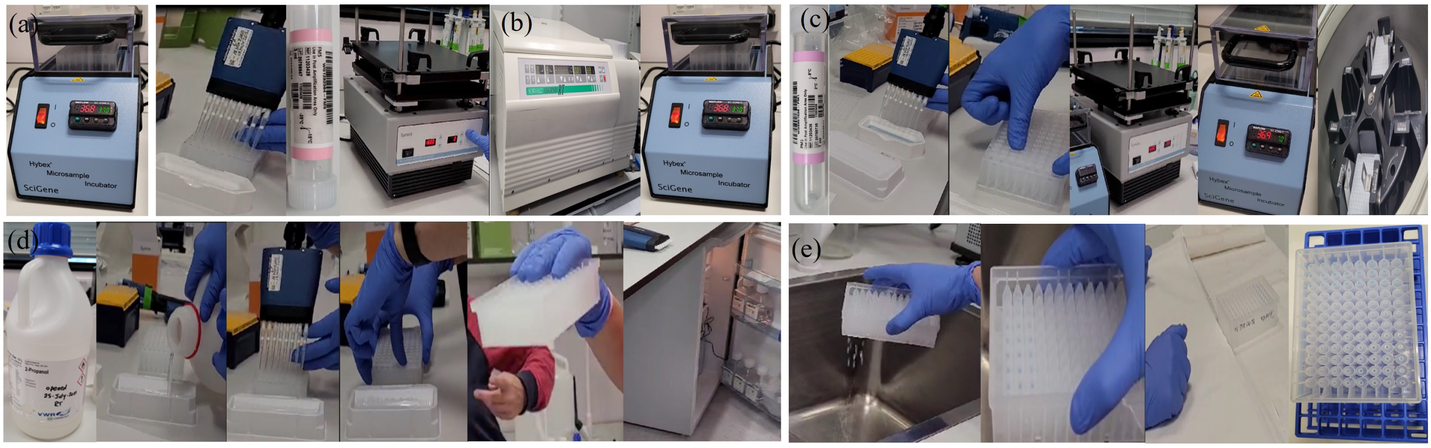

Figure S19. Representation of the lab work for DNA fragmentation and precipitation: (a) Preheat the block plate at 37°C. (b) Add 50 μl FMS to each sample well, vortex, centrifuge then heat the block for one hour. (c) Add 100 μl of PM1 to the samples, votex, incubate for 5 min and centrifuge at 4°C. (d) Add 300 μl 100% 2-propanol to each sample well, invert 10 times to mix then incubate in fridge for 30 min. (e) Drain the plate and place it in absorbent then leave it one hour to dry.

1.9. *DNA Resuspension*

The precipitated DNA is resuspended in this step by using RA1 [8].

Materials:

- RA1

Methods:

Prior to use, we made sure the heat sealer was at least 10 minutes heated, and we first set the Illumina Hybridisation Oven to 48°C. We repeatedly flipped the thawed RA1 vial to dissolve its contents in order to prepare the RA1 solution. To each pellet well in the MSA1 plate, we inserted 46 μl of RA1. We covered the plate with a foil heat seal, dull side down. We held the heat sealer sealing block down steadily and uniformly for five seconds to make sure the seal was correct. Once all the wells indentations were visible through the foil, we firmly rolled the rubber plate sealer over the plate. We resealed the plate if any of the wells were not clearly defined. Refer to Figure S20 for DNA resuspension.

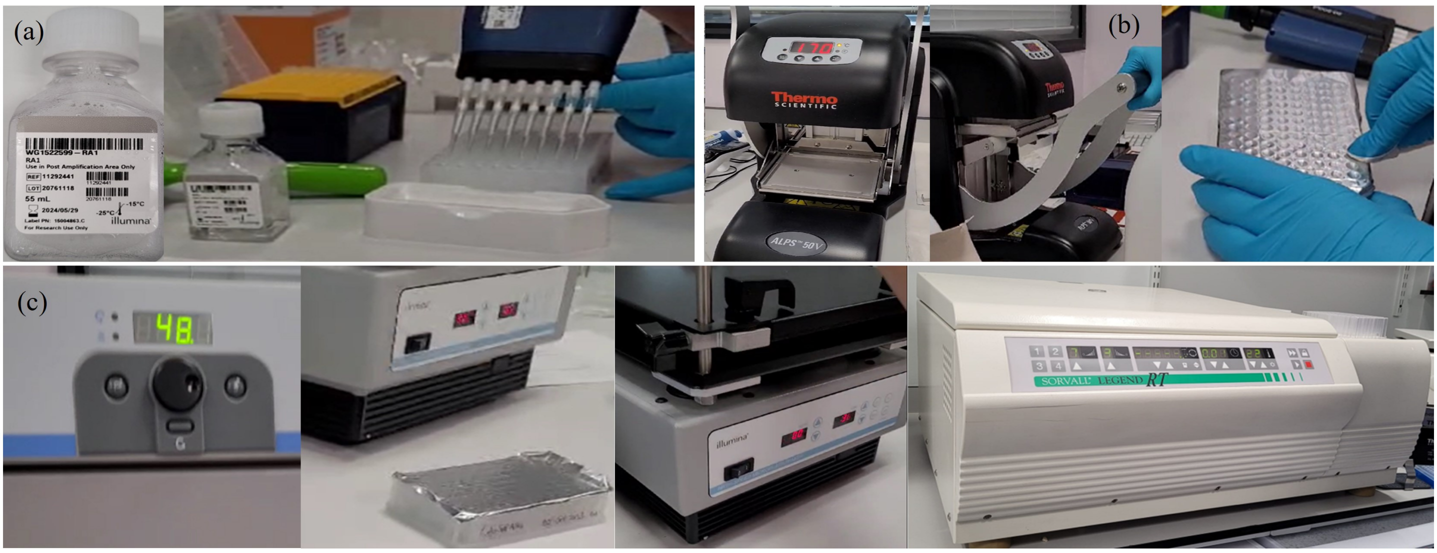

Figure S20. Representation of the lab work for DNA resuspension: (a) Add 46 μl to the wells of the MSA1 plate. (b) Seal the plate with foil. (c) Incubate the plate at 48°C in the oven, vortex, and then centrifuge.

1.10. *DNA Hybridisation to BeadChip*

The resuspended, fragmented DNA is dispensed onto BeadChips in this stage. Each DNA sample is then hybridised to a portion of the BeadChip by incubation [8].

Preparation:

The Illumina Hybridisation Oven was preheated to 48°C, and the heat block was prepared to 95°C. We placed the MSA1 plate on the heated heat block and let it incubate for 20 minutes in order to denature the DNA. We proceeded with the assembling procedures concurrently with this denaturation. All of the hybridisation chamber inserts, gaskets, and chambers were set up on the benchtop. To ensure correct alignment, each gasket was placed onto the chamber and firmly pressed into place.

In order to finish the assembly, we filled each BeadChip's top and bottom reservoirs with 200 μl of PB2. Without having to lock it, we quickly covered the chamber with the lid to stop evaporation. After that, the sealed chambers were placed on the benchtop and allowed to sit at room temperature for about an hour so that the DNA could load into the BeadChips. After the MSA1 plate had been incubated for 20 minutes, we moved it from the heat block to the benchtop and let it cool for 30 minutes at room temperature.

Method:

1.10.1. **Load DNA onto BeadChips:**

The MSA1 plate was first pulse centrifuged at 280 x g. Next, we handled the BeadChips by their ends and gently took them out of the package, keeping them away from the sample

inlets. To ensure that the barcode ends aligned correctly, each BeadChip was inserted into an insert. The foil seal on the MSA1 plate was then taken off. Next, we moved 26 μl of each sample from the MSA1 plate to the appropriate BeadChip sections. We closely monitored the loading port for any extra liquid as we let the DNA spread uniformly across the whole surface.

To make a bolus around the loading port, the area where there was no surplus liquid, we added any remaining sample from the amplification plate. These procedures guaranteed that the BeadChips were loaded precisely and carefully for our lab work.

1.10.2. **Set Up of BeadChips for Hybridisation:**

To ensure correct alignment, we put the inserts containing BeadChips into the hybridisation chamber. To keep the inserts in place, we inserted the back of the lid into the chamber and then gradually lowered the front. We made sure the lid sat squarely on the base without any gaps by firmly closing all four clamps. With the top logo facing us, the ready chamber was put into the Illumina Hybridisation Oven that had been preheated. We let it sit at 48°C for somewhere between 16 and 24 hours, depending on what our experiment needed. In order to preserve RA1 for use the next day, we stored it at 2°C to 8°C. It's crucial to remember that the MSA1 plate was disposed of safely. For the process of DNA hybridisation to BeadChip refer to Figure S21.

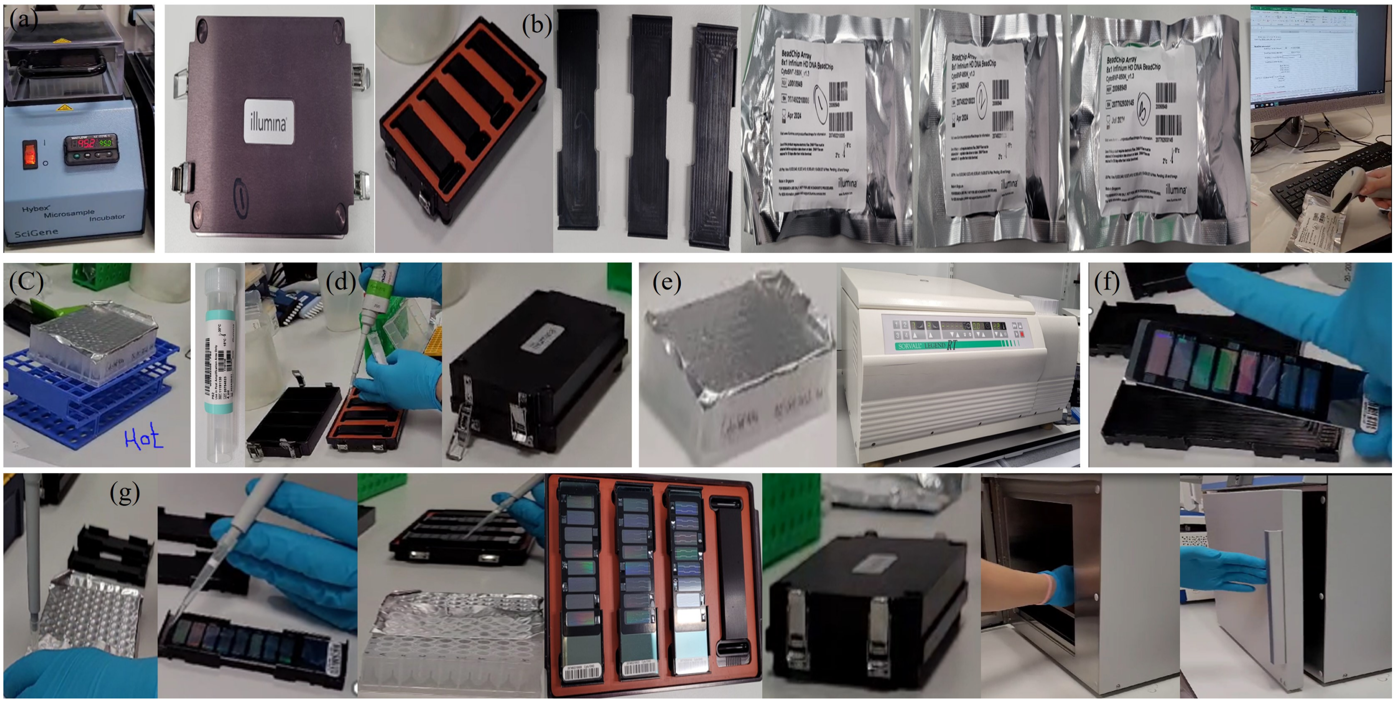

Figure S21. Representation of the lab work for BeadChip DNA Hybridisation: (a) Incubate the MSA1 plate in the oven at 95°C for 20 minutes to denature the DNA. (b) Assemble the hybridisation chambers. (c) Allow the plate to cool down. (d) Add 200 μl of buffer (PB2) to the top and bottom of the BeadChip reservoirs, then cover it. (e) Centrifuge the MSA1 plate. (f) Place the three BeadChips into their inserts. (g) Transfer 26 μl from the MSA1 plate to each section of the BeadChip, close the lid, and incubate at 48°C overnight.

1.11. ***Resuspend XC4***

To get ready for the Extend and Stain BeadChip phase, resuspend XC4 by filling the vial with 330 ml of fresh 100% EtOH.

Preparation:

Before continuing, we took each hybridisation chamber out of the hybridisation oven and gave it a chance to cool for 25 minutes. Next, we prepared two wash dishes, filled them with 200 ml of PB1, and labelled them so that they could be quickly identified. At the same time, we measured exactly 150 ml of PB1 using a graduated cylinder and filled the Multi-Sample BeadChip Alignment Fixture. We also took out of storage the parts that we needed for the Te-Flow flow-through chamber, which included the spacers, clamps, clean glass back plates, and black frames. This meticulous approach made sure we had everything we needed and were prepared for the next phases in our lab technique, which involved washing.

Method:

Place the wash rack with its wire handle attached into one of the wash dishes that holds 200 ml of PB1.

*1.12. **Perform Single-Base Extension***

We incubated 150 μl of RA1 five times, each time for 30 seconds. Then, 450 μl of XC1 was added and incubated for ten minutes. This was followed by 450 μl of XC2 and an identical incubation period. 200 μl of TEM was added after that, and we incubated for 15 minutes. After that, we added 450 μl of a 95% formamide/1 mM EDTA solution and incubated for 1 minute. We then repeated this process once and incubated for an additional 5 minutes. In order to match the STM tube temperature, we also altered the temperature of the chamber rack. Finally, to complete our exact and thorough washing procedure in the lab, 450 μl of XC3 was added and incubated for 1 minute, once again. Refer to Figure S22 for XC4 resuspension and the Single-Base Extension.

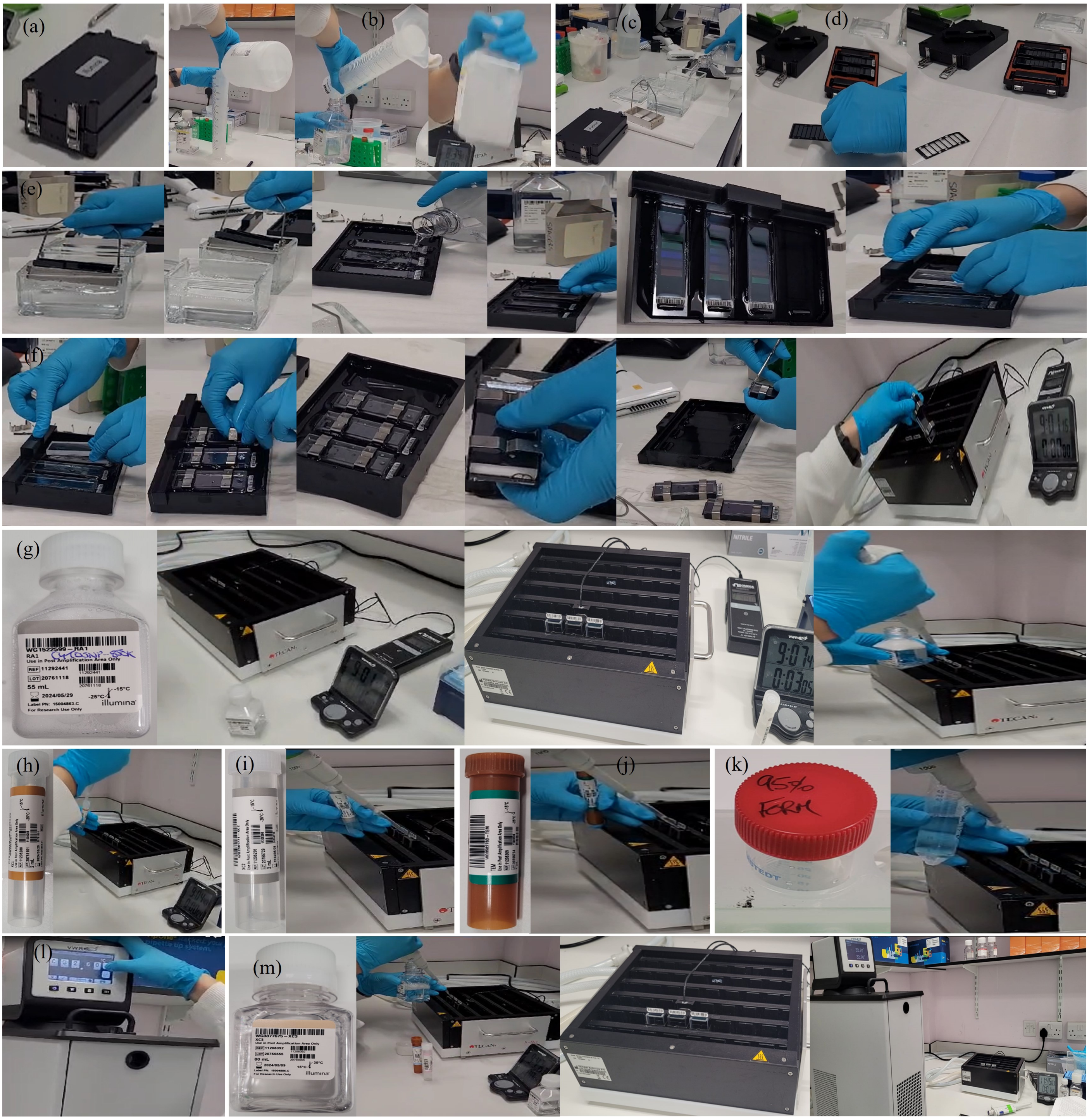

Figure S22. Representation of the lab work done for XC4 resuspension and the performance of the single-base extension: (a) Let the chamber cool for 10 min. (b) Add 330 ml of EtOH to the XC4 bottle, then shake well. (c) Add 200 ml of PB1 to two wash-dishes. (d,e,f) From the chamber component, remove the black frame from the BeadChip. Insert the BeadChip, into the wash-dish containing Buffer (PB1). Return the three washed BeadChips to the chamber and add buffer (PB1) make sure they are covered. Add the thin then add the glass and fix with clips. Cut the extra spacer and add the BeadChips to the chamber rack. (g) Adjust the chamber rack to be at a temperature of 44°C. Add 15 μl of RA1 and incubate for 30 seconds; repeat this 5 times. (h) Add 450 μl of XC1, then incubate for 10 minutes. (i) Add 450 μl of XC2, then incubate for 10 minutes. (j) Add 200 μl of TEM and incubate for 15 minutes. (k) Add 450 μl of formamide, then incubate for 1 minute and repeat this 1 time. Incubate for 5 minutes. (l) Change the temperature to 32°C on the STM tube. (m) Add 450 μl of XC3, then incubate for 1 min. Repeat this once.

1.13. *Stain BeadChips*

First, we added 250 μl of STM to the reservoir in each chamber and incubated for ten minutes. After that, we added 450 μl of XC3 and once again incubated for one minute. Following a 5-minute pause, we added 250 μl of ATM and incubated for 10 minutes. Next, we added 450 μl of XC3 and waited for an additional 5-minutes before continuing with the incubation for an additional minute. Every flow-through chamber underwent this cycle once more. The flow-through chambers were then promptly taken out of the chamber rack and set aside in alignment fixtures that were sitting on a lab bench, submerged in PB1 at room temperature.

1.14. *Wash and Coat BeadChips*

In order to remove BeadChips, we set up a rack to fit inside a vacuum desiccator and placed a clean tube rack on absorbent material. We filled two top-loading wash dishes, designated PB1 and XC4, with 310 ml of water, marked the water level, and then emptied it. Following that, we filled the PB1 wash dish with 310 ml of PB1. We immersed the staining rack in the PB1 wash dish, facing the locking arms and tab in our direction, in order to disassemble the flow-through chambers. We took off the two metal clamps and raised the glass back plate straight up for a deeper clean using a disassembly tool.

We took the BeadChip out of the black frame, being careful to hold it by the edges or barcode end, and carefully removed the spacer, being careful not to come into contact with the BeadChip stripes. Each flow-through chamber underwent this same procedure, wherein the BeadChips were positioned in the submerged staining rack with their arms locked and barcode facing away from us. We quickly raised and seated each BeadChip within the staining rack, lifting it up and down ten times to break the surface, in order to guarantee enough coverage and prevent BeadChip contact. We put 310 ml to the XC4 wash dish after letting the XC4 bottle shake to resuspend its contents, if necessary, after a 5-minute soak. After that, we moved the staining rack from the PB1 wash dish to the XC4 wash dish, soaking for an extra five minutes and doing the up-and-down motion ten times.

The staining rack was then taken out, set on the ready-made tube rack, making sure it was centred for even coating, and the staining rack handle was taken off. We took each BeadChip and held it by the barcode end with self-locking tweezers, then set it on a tube rack with the barcode facing up and towards us to dry. We arranged the BeadChips top to bottom and dried them for 50–55 minutes, making sure they didn't come into contact with one another or settle on the tube rack edge. Refer to Figure S23 for Staining, washing and Coating the BeadChip.

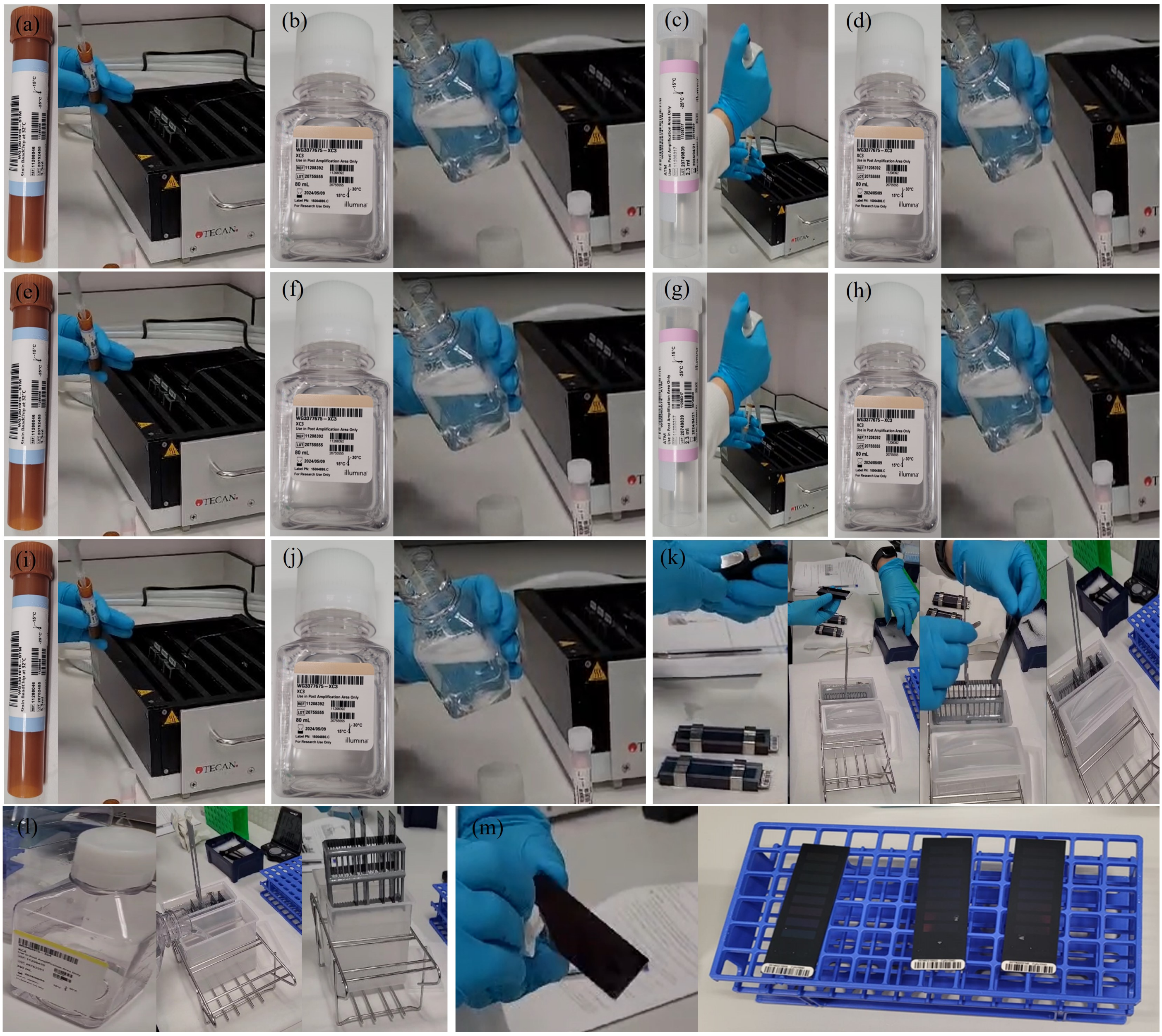

Figure S23. Representation of the lab work done for BeadChip staining, washing, and coating: (a) Add 250 μl of STM, then incubate for 10 minutes. (b) Add 450 μl of XC3, then incubate for 1 minute. Repeat this one time, then wait for 5 minutes. (c) Add 250 μl of ATM, and incubate for 10 minutes. (d) Add 450 μl of XC3, then incubate for 1 minute. Repeat this one time, then wait for 5 minutes. (e) Add 250 μl of STM, then incubate for 10 minutes. (f) Add 450 μl of XC3, then incubate for 1 minute. Repeat this one time, then wait for 5 minutes. (g) Add 250 μl of ATM, and incubate for 10 minutes. (h) Add 450 μl of XC3, then incubate for 1 minute. Repeat this one time, then wait for 5 minutes. (i) Add 250 μl of STM, then incubate for 10 minutes. (j) Add 450 μl of XC3, then incubate for 1 minute. Repeat this one time, then wait for 5 minutes. (k) Remove the flow-through chamber from the chamber rack and put the BeadChip in PB1. Move it up and down 10 times, then leave it for 5 minutes. (l) Put the BeadChip in XC4, move it up and down 10 times, then leave it for 5 minutes. (m) Clean it with ethanol and leave the BeadChip to dry for one hour.

1.15. *Scan and Analyse BeadChips*

The BeadChips must then be scanned using either the iScan or NextSeg 550 systems. During the scan, output files are created and saved in the designated output folder [9]. Refer to Figure S24 for BeadChip scanning.

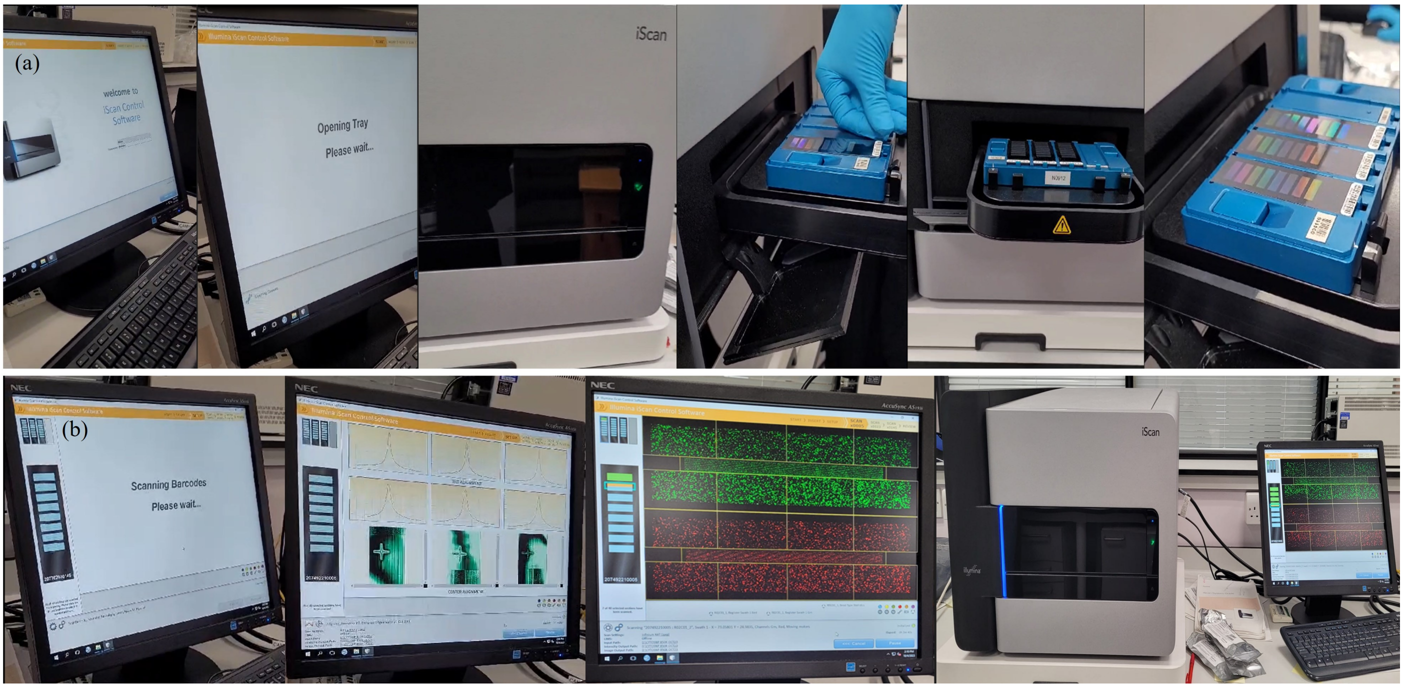

Figure S24. Representation of BeadChip scan and analysis: (a) Insert the BeadChips into the Illumina iScan machine. (b) The system scans the BeadChips, and the output files are created.

1.16. *SNP-Based Microarrays: A Simple Summary for Non-Specialists*

Genotyping using SNP-based microarrays for copy number variation involves identifying not only SNPs but also larger variations in the genome, called copy number variations. CNVs are large sections of the genome that can be duplicated or deleted in some individuals. These variations can affect gene function and sometimes influence traits or lead to diseases. SNP-based microarrays can detect CNVs by following a series of steps. First, a DNA tissue sample, is collected from the patient, just like in regular SNP genotyping. This DNA is then applied to a microarray chip that contains thousands or even millions of spots called probes. These probes are specifically designed to detect both SNPs, which represent small changes (like a single nucleotide difference), and CNVs, which involve larger genomic changes, such as sections of DNA being duplicated or deleted.

During hybridisation, DNA binds to the probes on the chip if there is a match in nucleotide sequences. For SNPs, the process detects which nucleotide is present, while for CNVs, the intensity of the hybridisation signal indicates whether extra copies or deletions of DNA segments are present. A strong signal may suggest additional copies, while a weak signal can imply a deletion. After hybridisation, the chip is scanned, and the results are processed to identify SNPs and assess CNVs. This combined data provides valuable insight into how genetic variations influence diseases, traits, and drug responses.

SNP arrays focus on specific SNP locations as they target known and important genetic variations. It provides a pre-selected target sites. SNP arrays are designed to look at the most informative spots in the genome, where variations SNPs are most likely to have an impact on traits or diseases. By focusing on these SNPs, the array can efficiently detect meaningful genetic differences without scanning the entire genome. Using SNP-based microarrays for CNV detection is advantageous for several reasons. It provides dual detection of both SNPs and CNVs in a single experiment, giving a more complete picture of genetic variation. This method is also quick and cost-effective, allowing for the study of large numbers of SNPs and CNVs simultaneously rather than requiring separate procedures. Additionally, CNVs are particularly important in disease studies, as they can play a significant role in conditions like cancer, autism, and developmental disorders,

where large sections of DNA may be duplicated or deleted. SNP-based microarrays for CNV genotyping allow researchers to examine both small and large genetic changes in an individual's DNA, offering a comprehensive view of genetic variation.

2. Random Forest Algorithm

Random Forest (RF) is a machine leaning algorithm that works by constructing several decision points during training. Its primary use is in classification and regression tasks, however, it can be adapted for other tasks such as feature selection and anomaly detection.

It utilises the concept of *decision trees* by splitting data into subsets based on the most significant feature at each node, creating branches that lead to decisions. Its goal is to increase the accuracy by lowering variance. Random Forest is an *ensemble learning* algorithm which combines multiple decision trees to produce a single more accurate model. It uses the principle that the combination of many weak models/trees will lead to a strong model [10].

Random Forest also use a technique called *Bagging/Bootstrap Aggregation* to create multiple subsets of the training data, with each subset being used to train a separate decision tree. These trees are thereafter combined i.e averaged in regression or majority vote in classification tasks to make the final prediction. In each tree, the model randomly selects a subset of features at each node thereby adding an element of *randomness* that helps in reducing over-fitting [11].

In regression tasks, a continuous prediction is made and the mean squared error (MSE) is used to determine how different the prediction is from the actual values. The average prediction is the average predictions across all trees. The formula (2) calculates the final prediction of a random forest regression model [11,12].

$$H(x) = \frac{1}{T}\sum_{t=i}^{T} h_t(x) \tag{1}$$

In classification tasks the final prediction for the classification is determined by majority voting. Refer to formula (3) [11,12].

$$H(x) = \text{mode}\{h_t(x) : t = 1, 2, \ldots, T\} \tag{2}$$

where,

- $H(x)$ Final predicted value returned by the model for input x;
- $h_t(x)$ Predicted value by a single tree t for input x;
- T Number of trees in the model.
- $\sum_{n=i}^{T} h_t(x)$ Sum of the predictions from the individual models $h_t(x)$, from the first model $index = i$ up to the last model $index = T$.
- $\frac{1}{T}$ To get the average prediction, the sum of the predictions is divided by the total number of models T.
- Mode {..} Function selects the most frequently occurring class among the predictions made by the individual models $h_t(x)$.

Random Forest has got several hyper-parameters which can be fine-tuned to optimise model performance. For instance performance usually increases with the increase in the number of trees, *n_estimators*, however, this increases computation time. Likewise, the maximum depth, *max_depth* determines the level of over-fitting or under-fitting in the model, shallower trees (less depth) reduce over-fitting but increase under-fitting. Over-fitting occurs when a model learns the underlying patterns in the training data, noise and random fluctuations. This results in a model that performs very well on the training data but poorly on test (unseen) data because it's too closely tied to the specifics of the

training set. Deep decision trees tend to over-fit because they can grow to very high depths, capturing every detail —including noise— in the training set. A deep tree has many decision nodes, which can allow it to create very specific decision boundaries, even for small variations in the data. While, under-fitting occurs when a model is too simple to capture the underlying patterns in the data. This means it performs poorly on both the training data and testing (unseen) data because it cannot represent the true complexity of the relationships in the data set. Shallow decision trees are more likely to under-fit because they don't have enough depth (decision nodes) to adequately capture the complexity of the data set. With fewer decision nodes, the model makes rough or simplistic predictions, missing important patterns.

The goal is to find the right balance between over-fitting and under-fitting (bias-variance trade-off) as shallow trees have high bias (they make broad, generalised predictions, often under-fitting the data) and low variance (they are stable and consistent across different training sets). Deep trees have low bias (they can capture detailed patterns in the data) but high variance (they are sensitive to fluctuations in the data, often over-fitting). In practice, the control of the depth of decision trees using hyper-parameters like max_depth in libraries such as scikit-learn is essential. A moderate tree depth can help balance the trade-off between bias and variance.

The number of features, *max_features*, helps determine how many features are considered when deciding the best split at each node. A smaller value (fewer features to consider at each node) introduces more randomness and reduces correlation among trees in an ensemble. This increased randomness can lead to better generalisation and reduced over-fitting, as individual trees are less likely to make the same errors. As a result, the ensemble performs better overall. However, there is a balance to strike—if too much randomness is introduced (i.e., *max_features* is too small), it can cause under-fitting, as the trees may not capture meaningful patterns in the data. Minimum sample split, *min_samples_split*, refers to the the number of samples required to split a node. Increasing the value prevent the model from learning overly specific pattern. *Bootstrap*, hyper-parameter specifies weather all trees will be build (trained) on the same original data (bootstrap=True) or not (bootstrap=False) [10,13].

Random forest offers a variety of advantages such as;

- *Reduction of Over-fitting*: It averages/maximum voting on multiple decision trees, thereby reducing the risk of over-fitting that can occur with individual decision trees. This makes RF generalisable on unseen data.
- *Robust to Noise/Outliers*: Random Forest is less sensitive to noise and outliers in the data because errors from individual trees are averaged out.
- *Robust for Large Data Set*: Random Forest is effective on large data set with high dimensional data as it build trees using random subsets of the features.
- *Feature Importance*: It provides feature importance scores which are useful in determining the most important features.

References

1. Illumina. DNA Copy Number and Loss of Heterozygosity Analysis Algorithms. https://www.illumina.com/documents/products/technotes/technote_cnv_algorithms.pdf accessed (18 July 2024), 2024.
2. Illumina. Microarray General Reference Materials. https://knowledge.illumina.com/microarray/general/microarray-general-reference_material-list/000002766 accessed (21 March 2024), 2024.
3. Tayside Biorepository. https://www.tissuebank.dundee.ac.uk accessed (27 April 2022), 2022.
4. Gupta, N. DNA extraction and polymerase chain reaction. *Journal of cytology* **2019**, *36*, 116–117.
5. Corporation, P. Maxwell® RSC DNA FFPE Kit Technical Manual. https://www.promega.co.uk/resources/protocols/technical-manuals/101/maxwell-rsc-dna-ffpe-kit-protocol/ accessed (27 April 2023), 2022.
6. Corporation, P. Maxwell® RSC Genomic DNA Kit Technical Manual. https://www.promega.co.uk/resources/protocols/technical-manuals/500/maxwell-rsc-genomic-dna-kit-protocol/ accessed (27 April 2023), 2022.

7. Illumina. InfiniumTM CytoSNP-850K v1.4 BeadChip Data Sheet. https://support.illumina.com/content/dam/illumina/gcs/assembled-assets/marketing-literature/infinium-cytosnp850k-data-sheet-m-gl-01507/infinium-cytosnp850k-data-sheet-m-gl-01507.pdf accessed (27 April 2023), 2023.
8. Illumina. Infinium CytoSNP-850K BeadChip Assay Reference Guide. https://support.illumina.com/ko-kr/downloads/infinium-cytosnp-850k-reference-guide-15046990.html accessed (27 April 2023), 2023.
9. Illumina. iScan System Guide. https://support-docs.illumina.com/ARR/iScan/Content/ARR/FrontPages/iscan.htm accessed (27 April 2023), 2023.
10. Breiman, L. Random forests. *Machine learning* **2001**, *45*, 5–32.
11. Louppe, G. Understanding random forests: From theory to practice. *arXiv preprint arXiv:1407.7502* **2014**.
12. Biau, G.; Scornet, E. A random forest guided tour. *Test* **2016**, *25*, 197–227.
13. Qi, Y. Random forest for bioinformatics. *Ensemble machine learning: Methods and applications* **2012**, pp. 307–323.

Disclaimer/Publisher's Note: The statements, opinions and data contained in all publications are solely those of the individual author(s) and contributor(s) and not of MDPI and/or the editor(s). MDPI and/or the editor(s) disclaim responsibility for any injury to people or property resulting from any ideas, methods, instructions or products referred to in the content.
